# Chemical Constituents from the Flowers of *Carthamus tinctorius* L. and Their Lung Protective Activity

**DOI:** 10.3390/molecules27113573

**Published:** 2022-06-02

**Authors:** Yanling Liu, Mengna Wang, Yangang Cao, Mengnan Zeng, Qinqin Zhang, Yingjie Ren, Xu Chen, Chen He, Xiling Fan, Xiaoke Zheng, Weisheng Feng

**Affiliations:** 1 School of Pharmacy, Henan University of Chinese Medicine, Zhengzhou 450046, China; liuyl9696@163.com (Y.L.); 15738125719@163.com (M.W.); caoyangang1987@126.com (Y.C.); 17320138484@163.com (M.Z.); zhangqq2020123@163.com (Q.Z.); renyingjie6666@163.com (Y.R.); 18638197038@163.com (X.C.); hhehechen@126.com (C.H.); fxl2020002061@163.com (X.F.); 2 The Engineering and Technology Center for Chinese Medicine, Development of Henan Province China, Zhengzhou 450046, China

**Keywords:** Asteraceae, *Carthamus tinctorius* L., flavonoids, sesquiterpenes, lung protective activity

## Abstract

A new flavonoid, saffloflavanside (**1**), a new sesquiterpene, safflomegastigside (**2**), and a new amide, saffloamide (**3**), together with twenty-two known compounds (**4**–**25**), were isolated from the flowers of *Carthamus tinctorius* L. Their structures were determined based on interpretation of their spectroscopic data and comparison with those reported in the literature. The protective effects against lipopolysaccharide (LPS)-stimulated damage on human normal lung epithelial (BEAS-2B) cells of the compounds were evaluated using MTT assay and cellular immunofluorescence assay. The results showed that compounds **2**–**3**, **8**–**11**, and **15**–**19** exhibited protective effects against LPS-induced damage to BEAS-2B cells. Moreover, compounds **2**–**3**, **8**–**11**, and **15**–**19** can significantly downregulate the level of nuclear translocation of NF-κB p-p65. In summary, this study revealed chemical constituents with lung protective activity from *C. tinctorius*, which may be developed as a drug for the treatment of lung injury.

## 1. Introduction

*Carthamus tinctorius* L., widely accepted as Safflower, belongs to the family of Asteraceae, mainly distributed in China, India, Iran, Egypt, and other countries [1]. It is an annual or biennial herbal plant mainly cultivated for its seeds, meals, and flowers, which primarily are rich in the orange-red dye (carthamin) and quality oil of polyunsaturated fatty acids [2]. Therefore, this plant is used for natural dyestuff, culinary, and textile purposes. More to the point, the dried flower of *C. tinctorius* is also clinically used to alleviate pain, increase circulation, and reduce blood-stasis syndrome with dysmenorrhoeal, amenorrhoea, trauma, and joint pain [3]. Pharmacological investigations have demonstrated that this plant possessed certain biological properties such as anti-inflammatory [4], cardioprotective [5], antitumor [6], anti-osteoporosis [7], and hepatoprotective effects [8]. Moreover, *C. tinctorius* shows effective outcomes in myocardial ischemia, coagulation, and thrombosis [9]. Concerning the phytochemistry of this plant, certain bioactive constituents have been isolated, such as flavonoids, phenylethanoid glycosides, coumarins, and polysaccharides [2].

Acute lung injury (ALI) is a continuum of pulmonary changes caused by various lung insults. The main pathological features of ALI are increased pulmonary vascular permeability, exudation of protein-rich fluid in the alveolar cavity, pulmonary edema, and hyaline membrane formation [10]. Lipopolysaccharide (LPS) can induce the apoptosis of lung epithelial cells and rapid influx of polymorphonuclear leukocytes (PMNs), causing the releases of proinflammatory cytokines, reactive oxygen species, and chemotactic factors [11]. The previous study demonstrated that flavonoids isolated from *C*. *tinctorius* could alleviate acute lung injury induced by LPS [12], which attracted our interest to search for more natural products with lung-protective activity from this plant.

## 2. Results and Discussion

### 2.1. Structure Characterization

**Figure 1:**
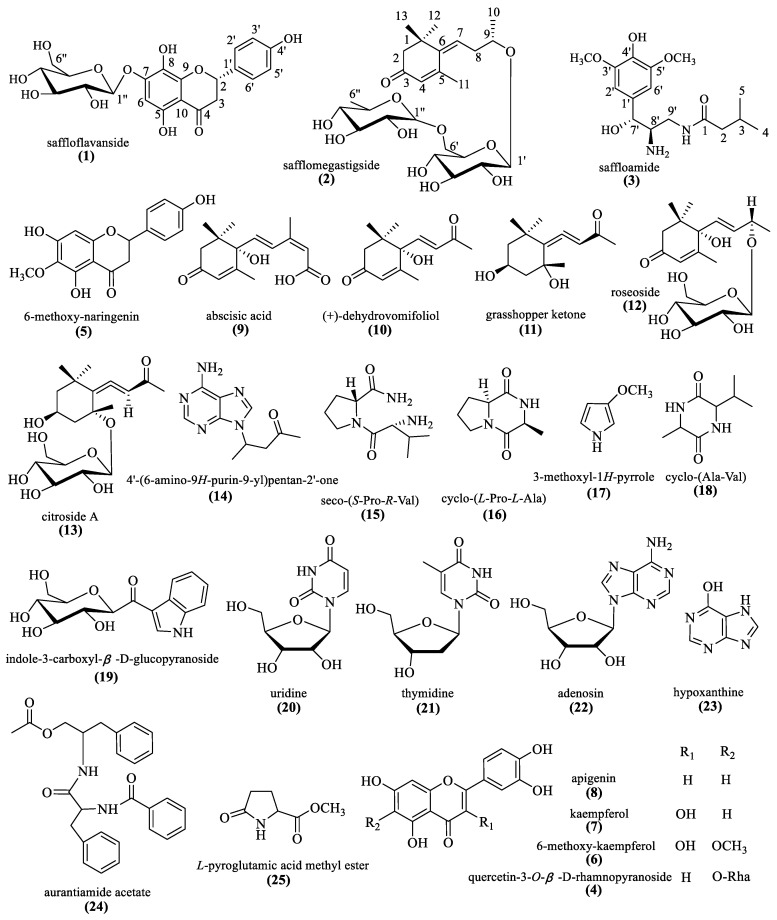
The chemical structures of compounds **1**–**25**.

The present chemical investigations on the extract of the flowers of *C*. *tinctorius* led to the characterization of three new compounds (**1**–**3**), as well as twenty-two known compounds (**4**–**25**) (Figure 1). Furthermore, their protective effects against LPS-induced BEAS-2B cells injury were evaluated.

Compound **1** was isolated as a green, amorphous solid and had a [M+H]^+^ ion peak at *m*/*z* 451.1260 in its HRESIMS, corresponding to the molecular formula C_21_H_23_O_11_. The ^1^H NMR data (Table 1) of **1** displayed the presence of a 1,4-disubstituted aromatic ring [*δ*_H_ 7.31 (2H, d, *J* = 8.4 Hz, H-2′, 6′), 6.80 (2H, d, *J* = 8.4 Hz, H-3′, 5′)], a 1,2,3,4,5-pentasubstituted aromatic ring [*δ*_H_ 6.36 (1H, s, H-6)], an oxymethine [*δ*_H_ 5.30 (1H, dd, *J* = 13.2, 3.1 Hz, H-2)], and an anomeric proton [*δ*_H_ 4.93 (1H, d, *J* = 7.4 Hz)]. The ^13^C NMR data (Table 1) and HSQC data revealed 21 carbon signals, which consisted of a carbonyl carbon [*δ*_C_ 199.5 (C-4)], twelve aromatic carbons [*δ*_C_ 159.1 (C-4′), 157.0 (C-5), 154.7 (C-7), 150.8 (C-9), 131.1 (C-1′), 129.1 (C-2′, 6′), 129.0 (C-8), 116.3 (C-3′, 5′), 105.0 (C-10), 96.1 (C-6)], an oxygenated methyine carbon [*δ*_C_ 80.9 (C-2)], a methylene carbon [*δ*_C_ 44.5 (C-3)], an anomeric carbon [*δ*_C_ 102.3 (C-1″)], and additional sugar signals [*δ*_C_ 78.4 (C-5″), 77.4 (C-3″), 74.6 (C-2″), 71.2 (C-4″), 62.3 (C-6″)], suggesting the presence of a hexose moiety. The hexose moiety was identified as D-glucose by chiral-phase HPLC analysis of the acid hydrolysate of **1**, which was attached to C-7 confirmed by the HMBC correlation of H-1″ (*δ*_H_ 4.93)/C-7 (*δ*_C_ 154.7). The *β*-configuration of the D-glucose was determined by the *J*-value (7.4 Hz) of the anomeric proton. These spectroscopic features suggested that the structure of **1** was very similar to that of 3′,4′,5,8-tetrahydroxyflavanone-7-*O*-*β*-D-glucopyranoside [13]. The obvious difference was the absence of the hydroxyl group at C-3′ in **1**. The (2*S*) absolute configuration was determined by the negative Cotton effect at 285 nm observed in its circular dichroic (CD) spectrum (Figure S11). Thus, the structure of compound **1** was determined as (2*S*)-4′,5,8-trihydroxyflavanone-7-*O*-*β*-D-glucopyranoside and named as saffloflavanside.

Compound **2** was obtained as a colorless amorphous powder and assigned a molecular formula of C_25_H_40_O_11_, as deduced from the ^13^C NMR and HRESIMS data. The ^1^H NMR data (Table 1) of **2** revealed the signals for two olefinic protons [*δ*_H_ 5.96 (1H, t, *J* = 6.8 Hz, H-7), 5.88 (1H, s, H-4)], an oxymethine proton [*δ*_H_ 3.93 (1H, m, H-9)], and five methyl groups [*δ*_H_ 2.26 (3H, s, H-13), 1.25 (3H, d, *J* = 6.2 Hz, H-6″), 1.23 (3H, d, *J* = 6.2 Hz, H-10), 1.18 (3H, s, H-12), 1.17 (3H, s, H-11)]. Besides, two anomeric proton resonances were observed at *δ*_H_ 4.73 (1H, brs, H-1″) and *δ*_H_ 4.32 (1H, d, *J* = 7.8 Hz, H-1′), while additional sugar signals appeared at *δ*_H_ 3.13–3.79, indicating that there were two glycosyl units in **2**. The ^13^C NMR data (Table 1) and HSQC data showed twenty-five carbon signals attributable to one carbonyl carbon [*δ*_C_ 202.0 (C-3)], four olefinic carbons [(*δ*_C_ 159.8 (C-5), 144.5 (C-6), 130.3 (C-7), 129.0 (C-4)], a quaternary carbon [*δ*_C_ 41.9 (C-1)], an oxymethine carbon [*δ*_C_ 76.0 (C-9)], two methylene carbons [*δ*_C_ 53.7 (C-2), 38.7 (C-8)], four methyl carbons [(*δ*_C_ 28.4 (C-11), 28.3 (C-12), 25.1 (C-13), 20.0 (C-10)], and twelve carbons of the hexosyl moieties [ *δ*_C_ 102.6 (C-1′), 78.1 (C-3′), 77.0 (C-5′), 75.1 (C-2′), 72.4 (C-4′), 68.8 (C-6′), *δ*_C_ 102.4 (C-1″), 74.0 (C-4″), 72.2 (C-3″), 72.0 (C-2″), 69.9 (C-5″), 18.1 (C-6″)]. Acid hydrolysis of **2** yielded D-glucose and L-rhamnose, which were determined by chiral-phase HPLC analysis. The relative configuration of the D-glucose was identified as the *β*-configuration, based on the coupling constant value of *δ*_H_ 4.32 (1H, d, *J* = 7.8 Hz, H-1′). Its 1D NMR data revealed similarities with those of the known compound (6*R*,9*R*)-eriojaposide B [14], except for the presence of an olefinic bond at C_6_-C_7_ and absence of an olefinic bond at C_7_-C_8_. In order to determine the absolute configuration of C-9, calculations of the gauge independent atomic orbital (GIAO) 1D NMR data for **2** were performed at the mPW1PW91/6-31G (d) level using CH_3_OH as the solvent, and the data were compared with the experimental values [15]. As a result, the (9*S*) absolute configuration was predicted as the correct structure with a DP4+ probability of 100.0% (Figure S74). Therefore, the structure of compound **2** was defined as (9*S*)-4,6-megastigmadien-3-one-9-*O*-[*α*-L-rhamnopyranoside-(1″→6′)-*β*-D-glucopyranoside] and named safflomegastigside.

**Figure 2:**
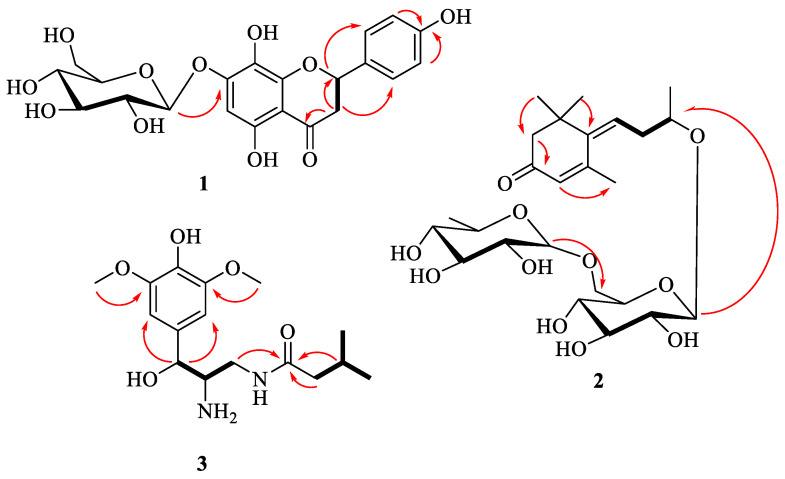
The key HMBC (arrow line) and ^1^H-^1^H COSY (bold line) correlations of compounds **1**–**3**.

Compound **3** was obtained as a colorless amorphous powder. The molecular formula of C_16_H_26_N_2_O_5_ was confirmed by the HRESIMS and NMR data. The ^1^H-NMR data of compound **3** exhibited a 1,3,4,5-tetrasubstituted aromatic ring [*δ*_H_ 6.70 (2H, s, H-2′, 6′)], an oxymethine [*δ*_H_ 4.94 (1H, d, *J* = 8.2 Hz, H-7′)], two methoxy groups [*δ*_H_ 3.86 (6H, s, 3′, 5′-OCH_3_)], and two methyl groups [*δ*_H_ 0.90 (3H, d, *J* = 3.5 Hz, H-4), 0.88 (3H, d, *J* = 3.5 Hz, C-5)]. Its ^13^C NMR and HSQC data (Table 1) indicated the presence of a carbonyl carbon [*δ*_C_ 174.4 (C-1)], six aromatic carbons [*δ*_C_ 149.4 (C-3′, 5′), 136.5 (C-4′), 133.4 (C-1′), 104.9 (C-2′, 6′)], an oxygenated methyine [*δ*_C_ 85.2 (C-7′)], a nitrogenated methyine [51.5 (C-8′)], a nitrogenated methylene [*δ*_C_ 64.1 (C-9′)], two methoxy groups [*δ*_C_ 56.9 (3′, 5′-OCH_3_)], a methyine carbon [*δ*_C_ 26.7 (C-3)], a methylene carbon [44.2 (C-2)], and two methyl groups [*δ*_C_ 22.7 (C-4, 5)]. The structure of the C_2_–C_5_ moiety in **3** was corroborated by the ^1^H-^1^H COSY correlations of H-3 (*δ*_H_ 1.94) with H-2 (*δ*_H_ 2.09), H-4 (*δ*_H_ 0.90), and H-5 (*δ*_H_ 0.88). The ^1^H-^1^H COSY crosspeaks from H-8′ (*δ*_H_ 2.51) to H-7′ (*δ*_H_ 4.94) and H-9′ (*δ*_H_ 64.1), as well as the HMBC correlations from H-7′ (*δ*_H_ 4.94) to C-2′ (*δ*_C_ 38.8) and C-6′ (*δ*_C_ 28.8) (Figure 2), disclosed that the C_7′_–C_9′_ moiety is attached to C-1′. The C_2_–C_5_ moiety and C_7′_–C_9′_ moiety are both attached to C-1, which was determined by the HMBC crosspeaks from H-2 to C-1 and from H-9′ to C-1. The coupling constant for the pair H-7′/H-8′ was employed to define the rotamer along the C-7′ to C-8′ bond. The corresponding coupling constant equals 8.2 Hz in **3**, indicating the dihedral angle (~180°) between these two protons in its model [16], which demonstrated that H-7′ and H-8′ are on different sides of the molecule.

Twenty-two known compounds were identified as quercetin-3-*O*-*β*-D-rhamnopyranoside (**4**) [17], 6-methoxy-naringenin (**5**) [18], 6-methoxy-kaempferol (**6**) [19], kaempferol (**7**) [20], apigenin (**8**) [21], abscisic acid (**9**) [22], (+)-dehydrovomifoliol (**10**) [23], grasshopper ketone (**11**) [24], roseoside (**12**) [25], citroside A (**13**) [23], 4′-(6-amino-9*H*-purin-9-yl)pentan-2′-one (**14**) [26], seco-(*S*-Pro-*R*-Val) (1**5**) [27], cyclo-(*L*-Pro-*L*-Ala) (**16**) [28], 3-methoxyl-1*H*-pyrrole (**17**) [29], cyclo-(Ala-Val) (**18**) [30], indole-3-carboxyl-*β*-D-glucopyranoside (**19**) [31], uridine (**20**) [32], thymidine (**21**) [33], adenosine (**22**) [34], hypoxanthine (**23**) [35], aurantiamide acetate (**24**) [36], and *L*-pyroglutamic acid methyl ester (**25**) [37].

### 2.2. Biological Activity

**Figure 3:**
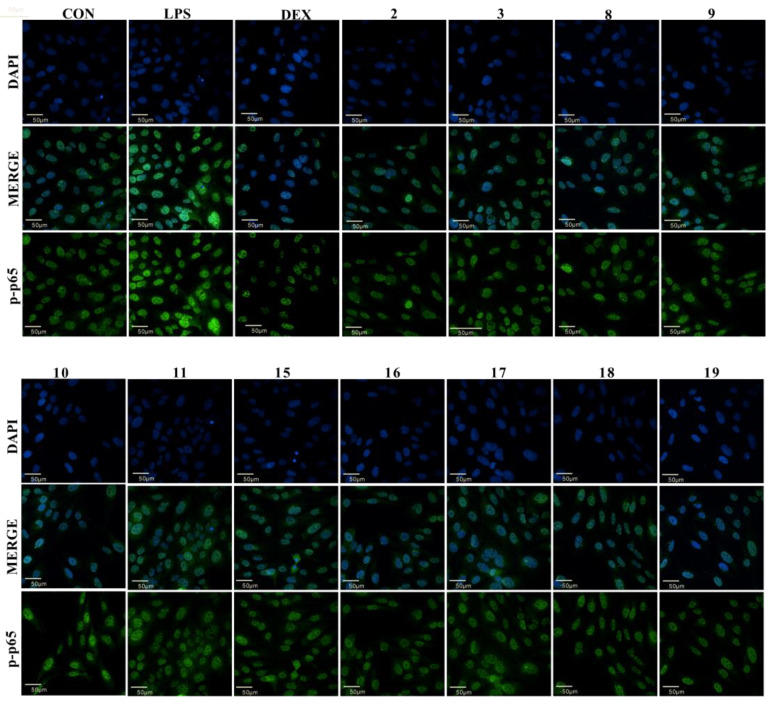
The effect of compounds **2**–**3**, **8**–**11**, and **15**–**19** on NF-KB p-p65 protein expression level of BEAS-2B. (Scale bars: 50 μm. Three independent experiments were performed, and the data were expressed as the mean ± SD.).

Dexamethasone (DEX) is regarded as an effective drug to relieve the level of pneumonia, while it can trigger side effects, such as neuromuscular, cardiovascular, and gastric motility disorders [38]. In preliminary in vitro bioassays, all the isolated compounds were evaluated for their protective effects against LPS-induced BEAS-2B cell injury. The results indicated that compounds **2**–**3**, **8**–**11**, and **15**–**19** exhibited a significant protective effect against LPS-induced BEAS-2B cell damage at a concentration of 10 μM with the DEX as the positive control drug (Table 2). Hence, compounds **2**–**3**, **8**–**11**, and **15**–**19** isolated from *C. tinctorius* may be used as potential drugs to treat lung injury. Furthermore, NF-κB is a ubiquitous nuclear transcriptional activator in the body, which is involved in the occurrence of inflammation and cellular immunity. NF-κB p-p65 is a key functional isoform, whose nuclear translocation level is proportional to the degree of NF-κB activation [39]. The nuclear translocation level of NF-κB p-p65 in cells was detected by the cell immunofluorescence technique. As shown in Figure 3, compounds **2**–**3**, **8**–**11**, **15**–**19**, and DEX can significantly downregulate the level of LPS-induced nuclear translocation compared with vehicle control, which indicated these compounds may reduce LPS-induced BEAS-2B cell damage by downregulating the nuclear translocation of NF-κB p-p65.

The previous study demonstrated that the total phenolics and flavonoids of the methanolic extracts of *Pulicaria petiolaris* (Asteraceae) decreased LPS-induced pulmonary inflammation, suggesting that *P. petiolaris* may be an important preventive strategy for the treatment of nonspecific pulmonary inflammation [40]. In addition, it reported that the sesquiterpenes of *Eupatorium lindleyanum* DC (Asteracea) significantly attenuated LPS-induced ALI [41]. This study indicates that flavonoids, sesquiterpenes, and alkaloids isolated from *C. tinctorius* (Asteracea) have a protective effect on ALI induced by LPS, indicating that plants in the Asteraceae family may have lung-protective potential, and that further research should focus on their use in pulmonary protection.

## 3. Experimental

### 3.1. General Experimental Procedures

Optical rotations were recorded by using a Rudolph AP-IV polarimeter (Rudolph, Hackettstown, NJ, USA). UV spectra were recorded on a ThermoEVO 300 spectrometer (Thermo, Waltham, MA, USA). IR spectra were recorded on a Thermo Nicolet IS 10 spectrometer (Thermo, Waltham, MA, USA). NMR spectra were acquired using a Bruker Avance III 500spectrometer (Bruker, Berlin, Germany). MS spectra were obtained using a Bruker maXis HD mass spectrometer (Bruker, Germany). Semipreparative HPLC separations were performed on a Saipuruisi LC 52 HPLC system with a UV/vis 50 detector (Saipuruisi, Beijing, China) and a YMC-Pack ODS-A column (20 × 250 mm, 5 μm; YMC, Kyoto, Japan). Monosaccharide elucidation was conducted on a Waters 2695 separation module equipped with an evaporative light scattering detector (ELSD) (Waters, Milford, MA, USA) using a CHIRALPAK AD-H column (4.6 × 250 mm) (Daicel Chiral Technologies Co., Ltd., Shanghai, China). Column chromatographies were performed using Toyopearl HW-40C, MCI gel CHP-20 (TOSOH Corp, Tokyo, Japan), Sephadex LH-20 (40–70 mm, Amersham Pharmacia Biotech AB, Uppsala, Sweden), and silica gel (100–200 mesh and 200–300 mesh, Marine Chemical Industry, Qingdao, China). The chemical reagents were supplied by the Beijing Chemical Plant (Beijing, China) and the Tianjin NO. 3 Reagent Plant (Tianjin, China).

### 3.2. Plant Material

The dried flowers of *C. tinctorius* were collected in December 2018 from Weihui city, Henan province, China, and identified by Professor Suiqing Chen of Henan University of Chinese Medicine. A voucher specimen (No. 20181212A) is deposited at the Department of Natural Medicinal Chemistry, Henan University of Chinese Medicine, Zhengzhou, China.

### 3.3. Extraction and Isolation

The dried flowers (18.0 kg) were extracted with 50% aqueous acetone twice at room temperature. The extract (5.6 kg) was suspended in water (10 L) and then successively extracted with petroleum ether (8 × 10 L), CH_2_Cl_2_ (8 × 10 L), EtOAc (8 × 10 L), n-BuOH (8 × 10 L).

The CH_2_Cl_2_ fraction (35 g) was separated by silica gel column chromatography (CC) eluted with petroleum ether-EtOAc (100:0−0:100) gradient and EtOAc-MeOH (100:0−0:100) gradient to yield seven fractions (D1−D7). Then fraction D7 (9.5 g) was chromatographed with silica gel CC (CH_2_Cl_2_-MeOH 100:1−10:1) to obtain four subfractions (D7-1−D7-4). Fraction D7-2 (2.0 g) was chromatographed with Sephadex LH-20 CC (MeOH-H_2_O 90:10) to give four subfractions (D7-2-1−D7-2-4). Then subfraction D7-2-2 (210.0 mg) was purified by semipreparative HPLC (MeOH-H_2_O 28:72) to produce compounds **9** (2.9 mg), **10** (3.2 mg), and **18** (3.3 mg). Subfraction D7-2-4 (150.3 mg) was purified by semipreparative HPLC (MeOH-H_2_O 42:58) to produce compounds **3** (17.2 mg), **15** (5.9 mg), and **16** (3.0 mg).

The EtOAc fraction (280 g) was separated by silica gel CC eluted with a CH_2_Cl_2_-MeOH (100:0−0:100) gradient system and yielded five fractions (E1−E8). Fraction E2 (20.0 g) was chromatographed with silica gel CC (CH_2_Cl_2_-MeOH 100:1−10:1) to obtain five subfractions (E2-1−E2-5). Fraction E2-2 (2.0 g) was chromatographed with Sephadex LH-20 CC (MeOH-H_2_O 70:30) to obtain five subfractions (E2-2-1−E2-2-5). Subfraction E2-2-3 (100.0 mg) was chromatographed with semipreparative HPLC (MeOH-H_2_O 10:90) to produce compounds **7** (4.1 mg) and compound **14** (7.2 mg). Then subfraction E2-2-5 (200.0 mg) was separated by Sephadex LH-20 CC (MeOH-H_2_O 70:30) and purified by semipreparative HPLC (MeOH-H_2_O 40:60) to produce compounds **5** (2.5 mg), **6** (4.2 mg), and **8** (3.1 mg). Fraction E4 (20 g) was loaded onto silica gel eluted with a CH_2_Cl_2_-MeOH (100:1−10:1) gradient system to give six subfractions (E4-1−E4-6). Fraction E4-5 (1.3 g) was chromatographed with Sephadex LH-20 CC with a MeOH-H_2_O (10:90−100:0) gradient to obtain six subfractions (E4-2-1−E4-2-6). Then subfraction E4-2-1 (110.6 mg) was purified by semipreparative HPLC (MeOH-H_2_O 30:70) to produce compounds **21** (7.3 mg) and **23** (6.0 mg). Subfraction E4-2-5 (200.0 mg) was chromatographed with semipreparative HPLC (MeOH-H_2_O 10:90) to produce compounds **24** (5.9 mg) and **25** (3.6 mg). Fraction E7 (49 g) was separated by MCI gel CHP-20 CC eluted with a MeOH-H_2_O (0:100−100:0) gradient system to obtain six subfractions (E7-1−E7-6). Then subfraction E7-1 (11.6 g) was chromatographed with silica gel CC eluted with a CH_2_Cl_2_-MeOH (50:1−5:1) gradient system to obtain five subfractions (E7-1-1−E7-1-5). Subfraction E7-1-2 (260.5 mg) was purified by semipreparative HPLC (MeOH-H_2_O 25:75) to produce compounds **15** (8.9 mg), **16** (4.0 mg), and **18** (3.0 mg). Subfraction E7-1-2 (200.0mg) was chromatographed with semipreparative HPLC (MeOH-H_2_O 25:75) to produce compounds **2** (6.0 mg), **17** (4.5 mg), and **20** (5.0 mg). Subfraction E7-2 (8.4 g) was loaded onto Sephadex LH-20 CC (MeOH-H_2_O 50:50) and yielded five subfractions (E7-2-1−E7-2-5). Subfraction E7-2-1 (600.0 mg) was separated by silica gel CC, eluted with a gradient system of CH_2_Cl_2_-MeOH (100:1−2:1) to give compounds **1** (3.2 mg), **11** (4.0 mg), **12** (3.0 mg), and **13** (7.4 mg). Subfraction E7-2-4 (380.0 mg) was applied to Toyopearl HW-40C CC (MeOH-H_2_O 70:30) and purified by semipreparative HPLC (MeOH-H_2_O 24:76) to produce compounds **4** (3.0 mg), **19** (4.3 mg), and **22** (5.0 mg).

Saffloflavanside (**1**): Yellow amorphous powder; [*α*]${}_{D}^{20}$ + 29.7 (*c* 0.02, MeOH); UV (MeOH) *λ*_max_: 202, 287, 367 nm; IR (iTR) ν_max_ 3383, 1646, 1519, 1455, 1373, 1311, 1218, 1078, 836 cm^−1^; HRESIMS *m*/*z* 451.1260 [M+H]^+^ (calcd for C_21_H_23_O_11_, 451.1234); ^1^ H and ^13^C NMR data, see Table 1.

Safflomegastigside (**2**): Colorless amorphous powder; [*α*]${}_{D}^{20}$ + 86.4 (*c* 0.01, MeOH); UV (MeOH) *λ*_max_: 202, 228, 289 nm; IR (iTR) ν_max_ 3386, 1675, 1652, 1380, 1203, 1139, 1043, 916, 838, 808, 724 cm^−1^; HRESIMS *m*/*z* 517.2656 [M+H]^+^ (calcd for C_25_H_41_O_11_, 517.2643); ^1^ H and ^13^C NMR data, see Table 1.

Saffloamide (**3**): Colorless amorphous powder; [*α*]${}_{D}^{20}$ + 192.3 (*c* 0.01, MeOH), UV (MeOH) *λ*_max_: 208, 240, 271 nm; IR (iTR) *ν*_max_: 3418, 2958, 1732, 1614, 1518, 1331, 1208, 1116, 837, 721cm^−1^; HRESIMS *m*/*z*: 327.1505 [M+H]^+^ (calcd. for C_16_H_27_N_2_O_5_, 327.1915). ^1^ H and ^13^C NMR data, see Table 3.

### 3.4. Computational Analysis

The systematic random conformational analysis of each conformer of **2** was performed in the GMMX software by using an MMFF94 force field, which afforded a few conformers each, with an energy cutoff of 5 kcal/mol to the global minima. The obtained conformers were further optimized using density functional theory (DFT) at the mPW1PW91/6-31G(d) level in CH_3_OH in the Gaussian 16W. The ^1^H and ^13^C NMR chemical shifts of the optimized stable conformers were calculated with the GIAO method at the mPW1PW91/6-31G(d) level in CH_3_OH. The calculated NMR data of these conformers were averaged according to the Boltzmann distribution theory [42].

### 3.5. Evaluation of the Protective Activities toward BEAS-2B Cells

#### 3.5.1. MTT Assay

The BEAS-2B cells were seeded in 96-well plates at 2.5 × 10^4^ cells/well for 24 h at 37 °C in a humidified atmosphere of 5 % CO_2_. Then the DMEM medium, the medium with LPS (10 μg/mL), the medium with DEX (1 μM), or the medium with test compounds (**1**–**25**) (10 μM) and LPS (10 μg/mL) were added, respectively, followed by the incubation for 24 h. Then 3-(4,5)-dimethylthiahiazo(-z-y1)-3,5-diphenytetrazoliumromide (MTT) solution (20 μL) was added to each well. After incubating for 4h, the solution was aspirated and dimethyl sulfoxide (DMSO, 150 μL) was added. The precipitate in each well was dissolved for 10 min. The optical density (OD) was determined at 490 nm using a microplate reader and calculated the cell viability [Viable cell number (%) = OD_490(treated cell culture)_/OD_490(vehicle control)_] [43]. The experiments were performed in triplicate.

#### 3.5.2. Cellular Immunofluorescence Assay

The BEAS-2B cells were distributed into 96-well plates. The final density per well was 2.5 × 10^4^ cells in 200 μL of medium. Then the cells were added to the DMEM medium, the medium with LPS (10 μg/mL), the medium with DEX (1 μM), and the medium with test compounds (**1**–**25**) (10 μM) and LPS (10 μg/mL) group, respectively. After 24 h, the supernatant was aspirated, and 4% paraformaldehyde diluted with PBS was added, and it was fixed at room temperature for 15 min. Then, 0.25% TritonX-100 was added to each well and incubated for 10 min for permeabilization. Next, cell culture plates were incubated with 1% (BSA+PBST) for 30 min at room temperature. They were then incubated with primary antibodies NF-κB p-p65 overnight. After washing, the plates were incubated with the anti-rabbit IgG for 1h. Then, they were washed with 0.2% PBST twice, and DAPI (50 μL 2 μg/mL) was added in incubation for 5 min. Anti-fluorescence quenching mounting tablets were used for mounting, and performed detection and analysis in a high-content instrument [44].

#### 3.5.3. Statistical Analysis

All data were analyzed by SPSS software version 26.0 (IBM, New York, NY, USA) and presented as the mean ± standard deviation. A one-way analysis of variance (One-Way ANOVA) was used for comparisons between groups. The differences were statistically significant when *p* < 0.05.

## 4. Conclusions

Three new compounds (**1**–**3**), together with twenty-two known compounds (**4**–**25**) were isolated from the flowers of *C. tinctorius.* Among the known compounds, **5**, **9**–**17**, **19**, **21**, and **24**–**25** were isolated from the plant for the first time. Most of the research on *C. tinctorius.* focuses on cardioprotective, antitumor, antithrombotic, anti-inflammatory, and hepatoprotective effects. In preliminary in vitro bioassays, the protective activities results showed that **2**–**3**, **8**–**11**, and **15**–**19** exhibited protective effects on BEAS-2B cell injury induced by LPS. Then, we will discover more bioactive compounds and carry out further research on the mechanism with potential compounds for the treatment of lung injury.

## Figures and Tables

**Table 1:** ^1^H NMR and ^13^C NMR data of compounds **1**–**2** (*δ* in ppm, *J* in Hz) in CD_3_OD.

Position	1		Position	2	
	*δ*_H_	*δ*_C_		*δ*_H_	*δ*_C_
2	5.30 (1H, dd, *J* = 13.2, 3.1)	80.9	1		41.9
3	3.14 (1H, dd, *J* = 17.3, 13.2) 2.72 (1H, dd, *J* = 17.3, 3.1)	44.5	2	2.28 (2H, s)	53.7
4		199.5	3		202.0
5		157.0	4	5.88 (1H, s)	129.0
6	6.36 (1H, s)	96.1	5		159.8
7		154.7	6		144.5
8		129.0	7	5.96 (1H, t, *J* = 6.8)	130.3
9		150.8	8	2.62 (1H, m) 2.52 (1H, m)	38.7
10		105.0	9	3.93 (1H, m)	76.0
1′		131.1	10	1.23 (3H, d, *J* = 6.2)	20.0
2′, 6′	7.31 (2H, d, *J* = 8.4)	129.1	11	2.26 (3H, s)	25.1
3′, 5′	6.80 (2H, d, *J* = 8.4)	116.3	12	1.18 (3H, s)	28.4
4′		159.1	13	1.17 (3H, s)	28.3
1″	4.93 (1H, d, *J* = 7.4)	102.3	1′	4.32 (1H, d, *J* = 7.8)	102.6
2″	3.51 (1H, m)	74.6	2′	3.13 (1H, m)	75.1
3″	3.46 (1H, m)	77.4	3′	3.32 (1H, m)	78.1
4″	3.38 (1H, m)	71.2	4′	3.64 (1H, m)	72.4
5″	3.43 (1H, m)	78.4	5′	3.40 (1H, m)	77.0
6″	3.86 (1H, d, *J* = 12.1) 3.67 (1H, m)	62.3	6′	3.98 (1H, dd, *J* = 11.3, 1.3) 3.54 (1H, dd, *J* = 11.3, 7.0)	68.8
			1″	4.73 (1H, s)	102.4
			2″	3.19 (1H, m)	72.0
			3″	3.79 (1H, m)	72.2
			4″	3.36 (1H, m)	74.0
			5″	3.62 (1H, m)	69.9
			6″	1.25 (3H, d, *J* = 6.2)	18.1

Data were recorded at 500 MHz for proton and at 125 MHz for carbon.

**Table 2:** The protective effect of compounds **1–25** on BEAS-2B cell injury induced by LPS.

Group	Dose	Cell Viability
CON	---	1.000 ± 0.043 **
LPS	10 μg/mL	0.879 ± 0.065
DEX	1 μM	0.959 ± 0.017 **
**1**	10 μM	0.869 ± 0.046
**2**	10 μM	0.937 ± 0.039 *
**3**	10 μM	0.963 ± 0.034 **
**4**	10 μM	0.931 ± 0.016
**5**	10 μM	0.843 ± 0.006
**6**	10 μM	0.845 ± 0.022
**7**	10 μM	0.907 ± 0.030
**8**	10 μM	0.949 ± 0.041 **
**9**	10 μM	0.949 ± 0.021 **
**10**	10 μM	1.029 ± 0.062 **
**11**	10 μM	0.964 ± 0.034 **
**12**	10 μM	0.929 ± 0.013
**13**	10 μM	0.909 ± 0.028
**14**	10 μM	0.917 ± 0.012
**15**	10 μM	0.990 ± 0.021 **
**16**	10 μM	1.014 ± 0.036 **
**17**	10 μM	0.957 ± 0.034 **
**18**	10 μM	0.954 ± 0.064 **
**19**	10 μM	0.976 ± 0.054 **
**20**	10 μM	0.912 ± 0.026
**21**	10 μM	0.845 ± 0.022
**22**	10 μM	0.843 ± 0.006
**23**	10 μM	0.882 ± 0.015
**24**	10 μM	0.914 ± 0.009
**25**	10 μM	0.907 ± 0.030

* *p* < 0.05; ** *p* < 0.01 compared with the LPS group. Three independent experiments were performed, and the data were expressed as the mean ± SD.

**Table 3:** ^1^H NMR and ^13^C NMR data of compound **3** (*δ* in ppm, *J* in Hz) in CD_3_OD.

Position	3	
	*δ*_H_	*δ*_C_
1		174.4
2	2.09 (2H, dd, *J* = 7.5, 2.8)	44.2
3	1.94 (1H, m)	26.7
4	0.90 (3H, d, *J* = 3.5)	22.7
5	0.88 (3H, d, *J* = 3.5)	22.7
1′		133.4
2′, 6′	6.70 (2H, s)	104.9
3′, 5′		149.4
4′		136.5
7′	4.94 (1H, d, *J* = 8.2)	85.2
8′	2.51 (1H, m)	51.5
9′	4.32 (1H, dd, *J* = 11.5, 4.1) 4.25 (1H, dd, *J* = 11.5, 5.2)	64.1
2′, 6′-OCH_3_	3.86 (6H, s)	56.9

Data were recorded at 500 MHz for proton and at 125 MHz for carbon.

## Data Availability

The data presented in this study are available in the Supplementary Materials.

## Supplementary Materials

The following supporting information can be downloaded at: https://www.mdpi.com/article/10.3390/molecules27113573/s1, Figures S1–S7: ^1^H-NMR, ^13^C-NMR and 2D-NMR spectra of compound **1**, Figures S8–S11: HR-ESI-MS, IR, CD and UV spectra of compound **1**; Figures S12–S17: ^1^H-NMR, ^13^C-NMR and 2D-NMR spectra of compound **2**, Figures S18–S20: HR-ESI-MS, IR and UV spectra of compound **2**; Figures S21–S26: ^1^H-NMR, ^13^C-NMR and 2D-NMR spectra of compound 3, Figures S27–S29: HR-ESI-MS, IR and UV spectra of compound **3**; Figures S30–S73: 1H-NMR, 13C-NMR spectra of compounds **4**–**25**; Figure S74: DP4+ evaluation of theoretical and experimental data of **2**.

## Sample Availability

Samples of the compounds are available from the authors.
