# Supplementary material for: Chemical Constituents from the Flowers of Carthamus tinctorius L. and Their Lung Protective Activity
Source: Molecules. 2022 Jun 2;27(11):3573. doi: 10.3390/molecules27113573 (PMC9182397; doi:10.3390/molecules27113573)
Supplement: Supplementary file 1 [file molecules-27-03573-s001.zip › molecules-1736899-supplementary.pdf]

Chemical constituents from the flowers of *Carthamus tinctorius* L. and their lung protective activity

Yan-ling Liu[1,2], Meng-na Wang[1,2], Yan-gang Cao[1,2], Meng-nan Zeng [1,2], Qin-qin Zhang [1,2], Ying-jie Ren[1,2], Xu Chen[1,2], Chen He[1,2], Xi-ling Fan[1,2], Xiao-ke Zheng[1,2*],Wei-sheng Feng[1,2*]

[1]*School of Pharmacy, Henan University of Chinese Medicine, Zhengzhou 450046, China*

[2]*The Engineering and Technology Center for Chinese Medicine Development of Henan Province China，Zhengzhou 450046, China*

*Corresponding author:

School of Pharmacy, Henan University of Chinese Medicine, Zhengzhou 450046, P.R. China.

*Corresponding author

E-mail addresses: zhengxk.2006@163.com (XK Zheng), fwsh@hactcm.edu.cn (WS Feng)

Contents

Figure S1. ^1H NMR spectrum (500MHz, CD_3OD) of **1**
Figure S2. ^{13}C NMR spectrum (125MHz, CD_3OD) of **1**
Figure S3. DEPT135 spectrum of **1**
Figure S4. ^1H-^1H COSY spectrum of **1**
Figure S5. HSQC spectrum of **1**
Figure S6. HMBC spectrum of **1**
Figure S7. NOESY spectrum of **1**
Figure S8. HR-ESI-MS spectrum of compound **1**
Figure S9. UV spectrum of **1**
Figure S10. IR spectrum of **1**
Figure S11. CD spectrum of **1**
Figure S12. ^1H NMR spectrum (500MHz, CD_3OD) of **2**
Figure S13. ^{13}C NMR spectrum (125MHz, CD_3OD) of **2**
Figure S14. ^1H-^1H COSY spectrum of **2**
Figure S15. HSQC spectrum of **2**
Figure S16. HMBC spectrum of **2**
Figure S17. NOESY spectrum of **2**
Figure S18. HR-ESI-MS spectrum of compound **2**
Figure S19. UV spectrum of **2**
Figure S20. IR spectrum of **2**
Figure S21. ^1H NMR spectrum (500MHz, CD_3OD) of **3**
Figure S22. ^{13}C NMR spectrum (125MHz, CD_3OD) of **3**
Figure S23. DEPT135 spectrum of **3**
Figure S24. ^1H-^1H COSY spectrum of **3**
Figure S25. HSQC spectrum of **3**
Figure S26. HMBC spectrum of **3**
Figure S27. HR-ESI-MS spectrum of compound **3**
Figure S28. UV spectrum of **3**
Figure S29. IR spectrum of **3**
Figure S30. ^1H NMR spectrum (500MHz, CD_3OD) of **4**
Figure S31. ^{13}C NMR spectrum (125MHz, CD_3OD) of**4**
Figure S32. ^1H NMR spectrum (500MHz, CD_3OD) of **5**
Figure S33. ^{13}C NMR spectrum (125MHz, CD_3OD) of **5**
Figure S34. ^1H NMR spectrum (500MHz, CD_3OD) of **6**
Figure S35. ^{13}C NMR spectrum (125MHz, CD_3OD) of **6**
Figure S36. ^1H NMR spectrum (500MHz, CD_3OD) of **7**
Figure S37. ^{13}C NMR spectrum (125MHz, CD_3OD) of **7**
Figure S38. ^1H NMR spectrum (500MHz, CD_3OD) of **8**
Figure S39. ^{13}C NMR spectrum (125MHz, CD_3OD) of **8**
Figure S40. ^1H NMR spectrum (500MHz, CD_3OD) of **9**
Figure S41. ^{13}C NMR spectrum (125MHz, CD_3OD) of **9**
Figure S42. ^1H NMR spectrum (500MHz, CD_3OD) of **10**

Figure S43. ^{13}C NMR spectrum (125MHz, CD_3OD) of **10**
Figure S44. ^{1}H NMR spectrum (500MHz, CD_3OD) of **11**
Figure S45. ^{13}C NMR spectrum (125MHz, CD_3OD) of **11**
Figure S46. ^{1}H NMR spectrum (500MHz, CD_3OD) of **12**
Figure S47. ^{13}C NMR spectrum (125MHz, CD_3OD) of **12**
Figure S48. ^{1}H NMR spectrum (500MHz, CD_3OD) of **13**
Figure S49. ^{13}C NMR spectrum (125MHz, CD_3OD) of **13**
Figure S50. ^{1}H NMR spectrum (500MHz, CD_3OD) of **14**
Figure S51. ^{13}C NMR spectrum (125MHz, CD_3OD) of **14**
Figure S52. ^{1}H NMR spectrum (500MHz, CD_3OD) of **15**
Figure S53. ^{13}C NMR spectrum (125MHz, CD_3OD) of **15**
Figure S54. ^{1}H NMR spectrum (500MHz, CD_3OD) of **16**
Figure S55. ^{13}C NMR spectrum (125MHz, CD_3OD) of **16**
Figure S56. ^{1}H NMR spectrum (500MHz, CD_3OD) of **17**
Figure S57. ^{13}C NMR spectrum (125MHz, CD_3OD) of **17**
Figure S58. ^{1}H NMR spectrum (500MHz, CD_3OD) of **18**
Figure S59. ^{13}C NMR spectrum (125MHz, CD_3OD) of **18**
Figure S60. ^{1}H NMR spectrum (500MHz, CD_3OD) of **19**
Figure S61. ^{13}C NMR spectrum (125MHz, CD_3OD) of **19**
Figure S62. ^{1}H NMR spectrum (500MHz, CD_3OD) of **20**
Figure S63. ^{13}C NMR spectrum (125MHz, CD_3OD) of **20**
Figure S64. ^{1}H NMR spectrum (500MHz, CD_3OD) of **21**
Figure S65. ^{13}C NMR spectrum (125MHz, CD_3OD) of **21**
Figure S66. ^{1}H NMR spectrum (500MHz, CD_3OD) of **22**
Figure S67. ^{13}C NMR spectrum (125MHz, CD_3OD) of **22**
Figure S68. ^{1}H NMR spectrum (500MHz, CD_3OD) of **23**
Figure S69. ^{13}C NMR spectrum (125MHz, CD_3OD) of **23**
Figure S70. ^{1}H NMR spectrum (500MHz, CD_3OD) of **24**
Figure S71. ^{13}C NMR spectrum (125MHz, CD_3OD) of **24**
Figure S72. ^{1}H NMR spectrum (500MHz, CD_3OD) of **25**
Figure S73. ^{13}C NMR spectrum (125MHz, CD_3OD) of **25**
Figure S74. DP4+ evaluation of theoretical and experimental data of **2**

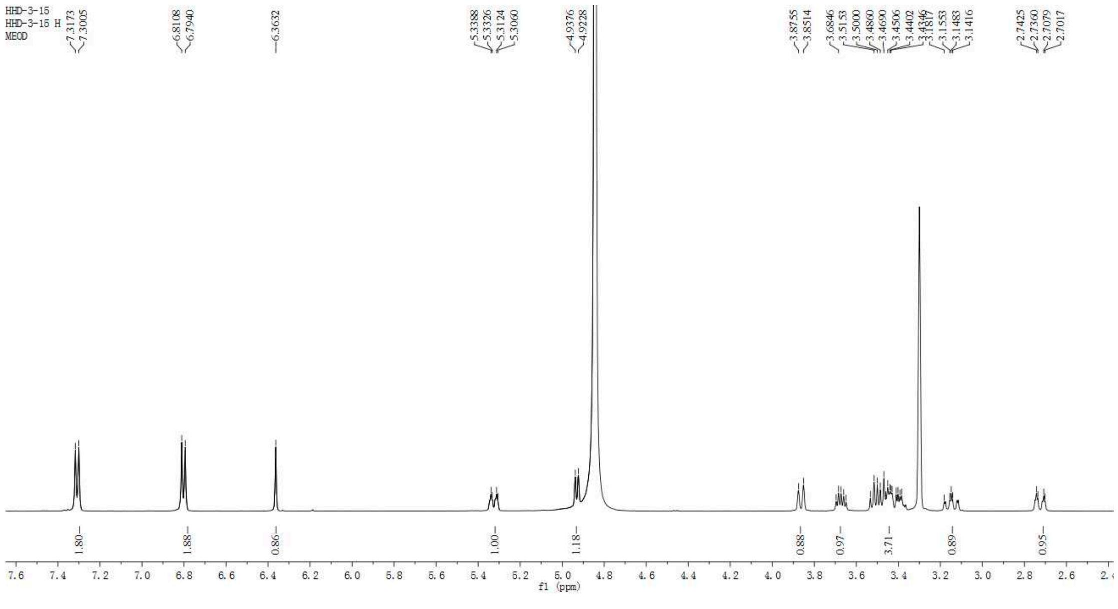

Figure S1. ^{1}H NMR spectrum (500MHz, CD_3OD) of **1**

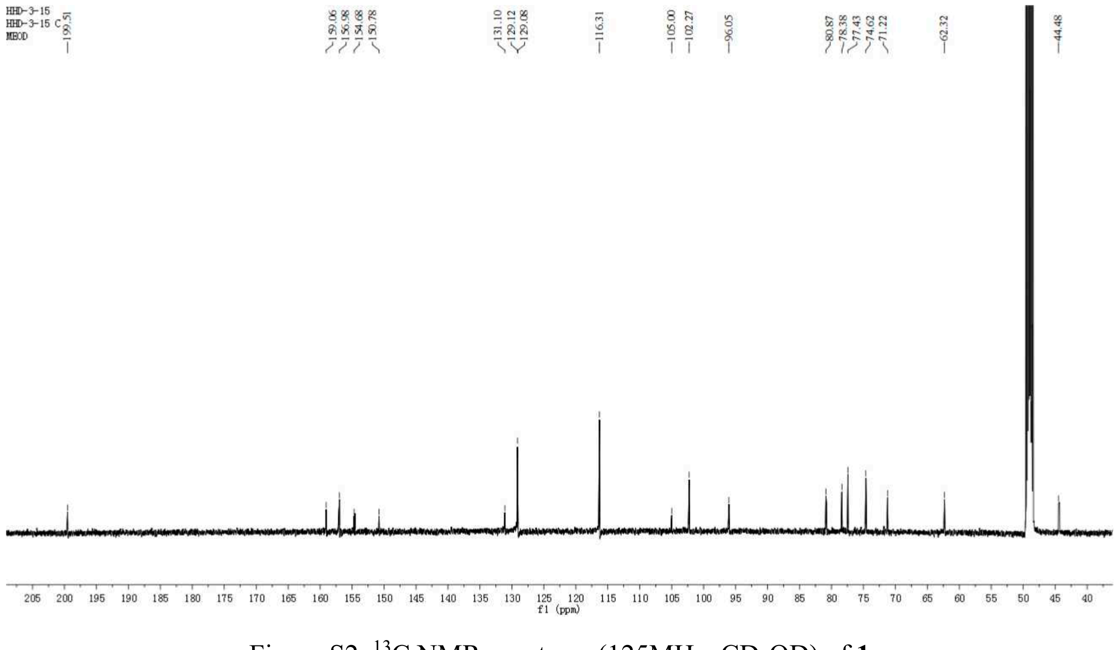

Figure S2. ^{13}C NMR spectrum (125MHz, CD_3OD) of **1**

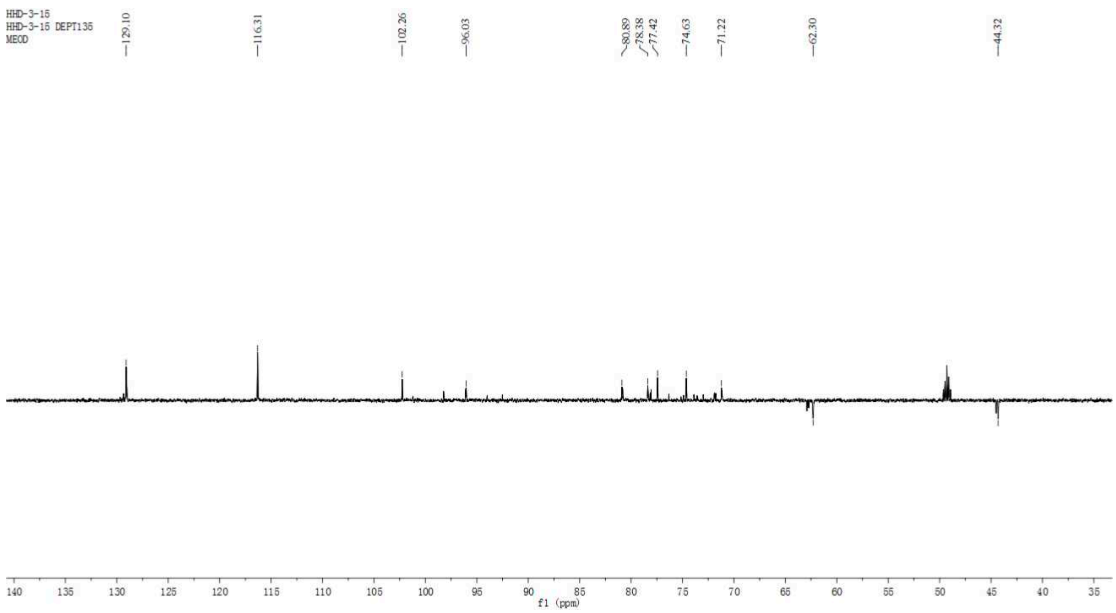

Figure S3. DEPT135 spectrum of **1**

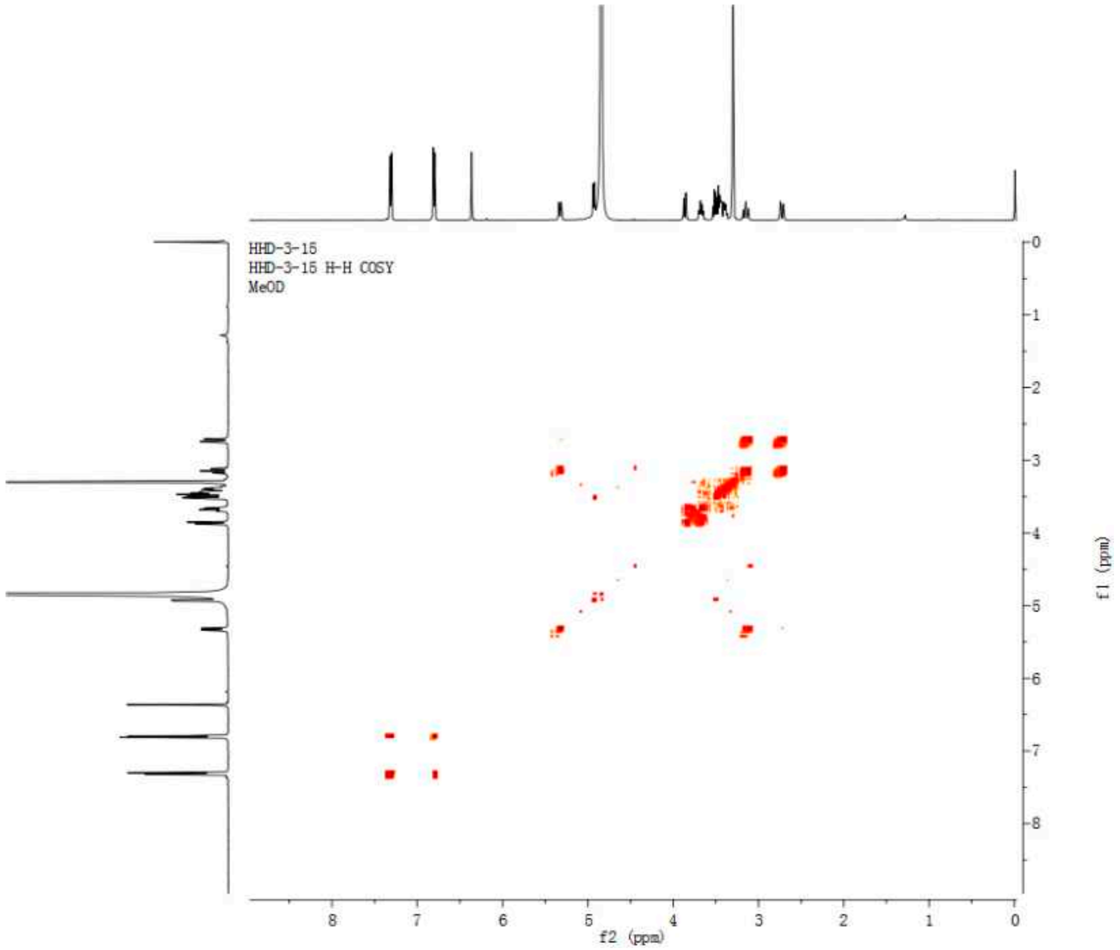

Figure S4. ^{1}H-^{1}H COSY spectrum of **1**

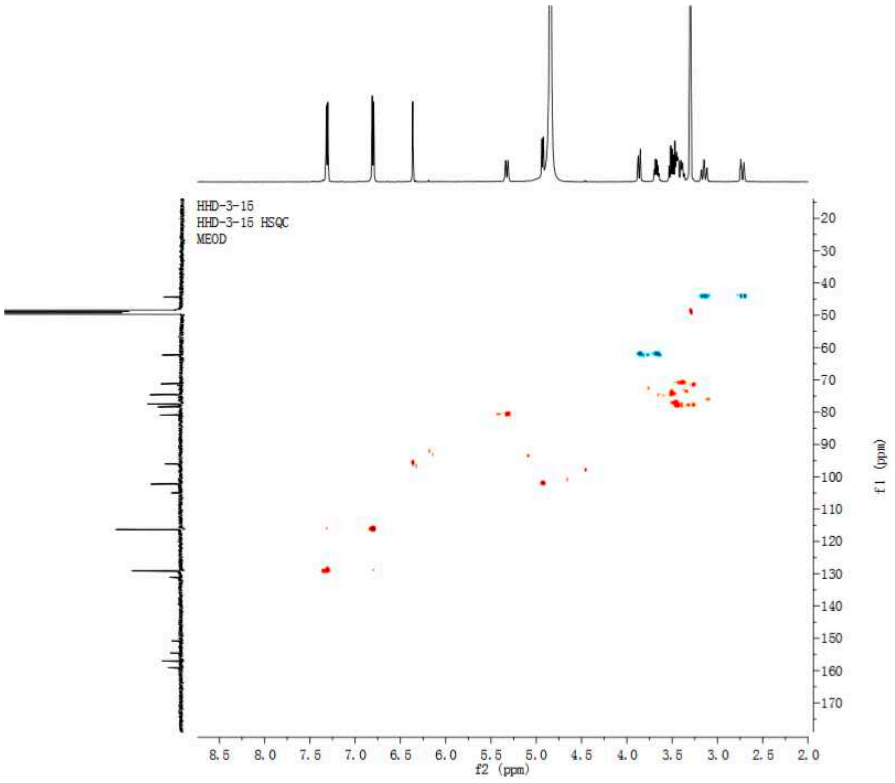

Figure S5. HSQC spectrum of **1**

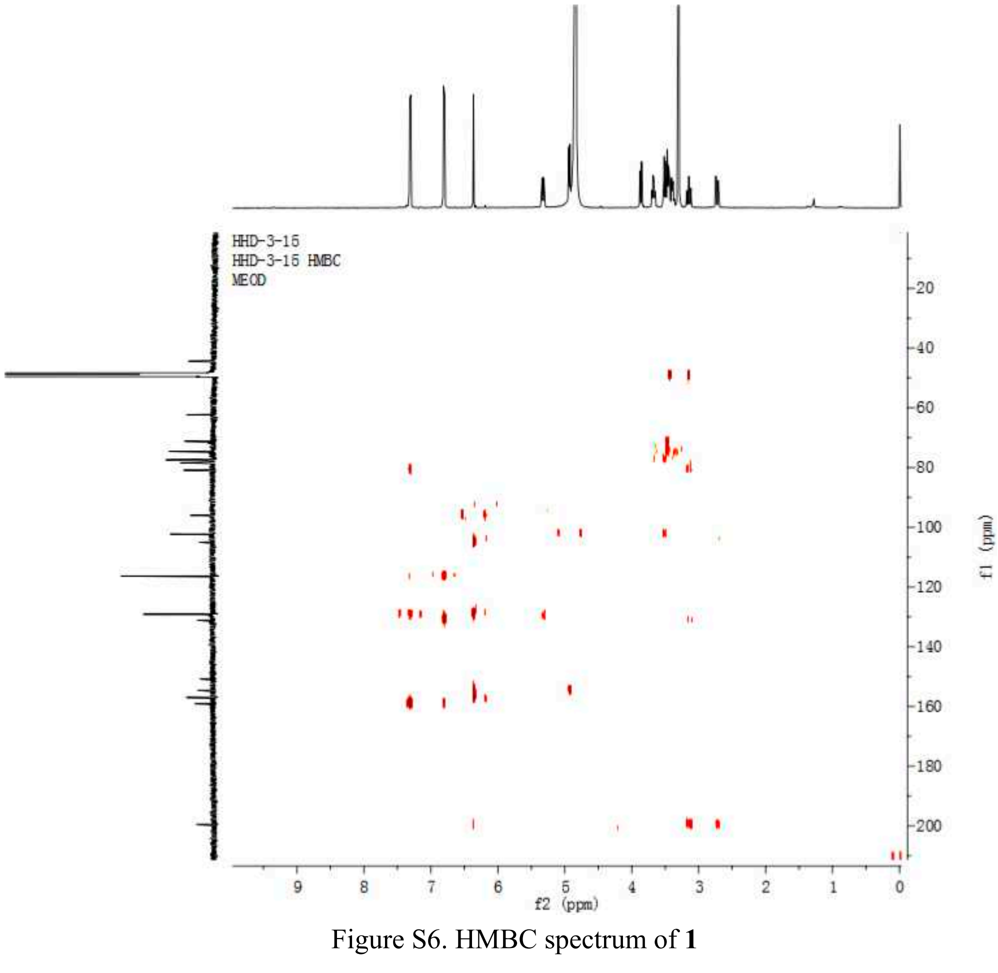

Figure S6. HMBC spectrum of **1**

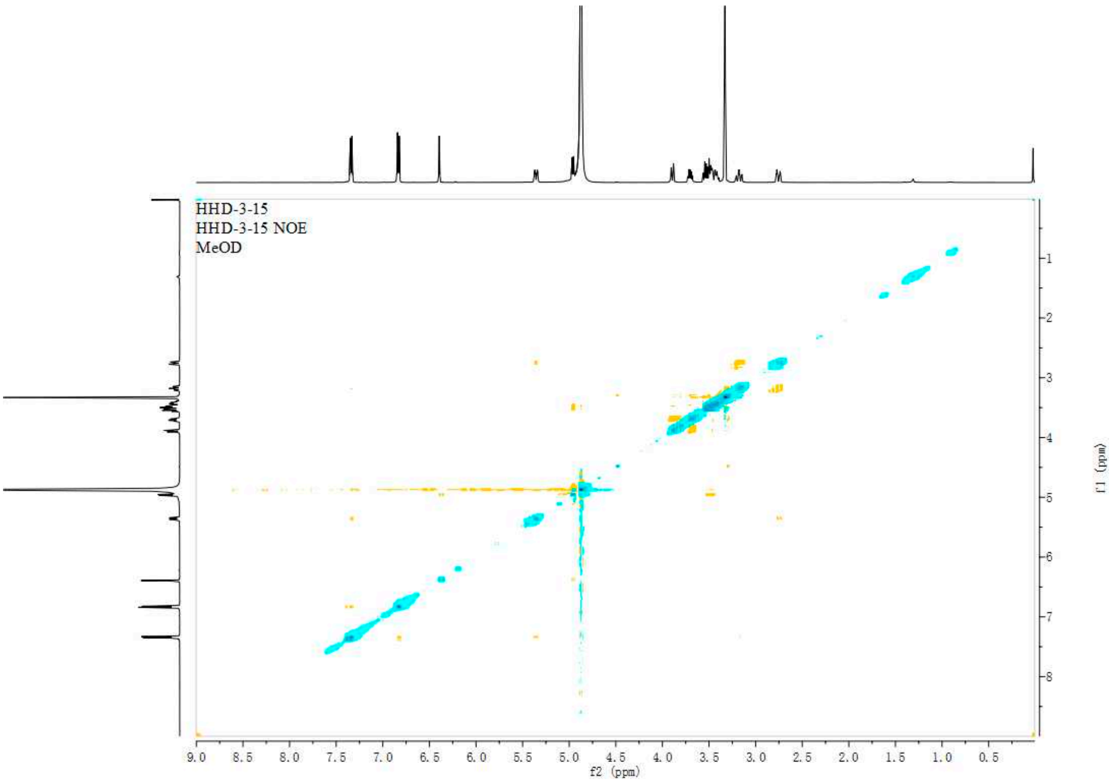

Figure S7. NOESY spectrum of **1**

Display Report

Analysis Info

Analysis Name	\\ESI-PC\data-D\Data\GJH\WMN\20201126\HHD3-15.d	Acquisition Date	2020/11/27 21:44:51
Method	tune_pos_standard_20141031.m	Operator	Demo User
Sample Name	HHD3-15	Instrument	maXis HD 1820881.21303
Comment			

Acquisition Parameter

Source Type	ESI	Ion Polarity	Positive	Set Nebulizer	0.3 Bar
Focus	Active	Set Capillary	3500 V	Set Dry Heater	200 ¡ãC
Scan Begin	50 m/z	Set End Plate Offset	-500 V	Set Dry Gas	4.0 l/min
Scan End	3000 m/z	Set Charging Voltage	2000 V	Set Divert Valve	Waste
		Set Corona	0 nA	Set APCI Heater	0 ¡ãC

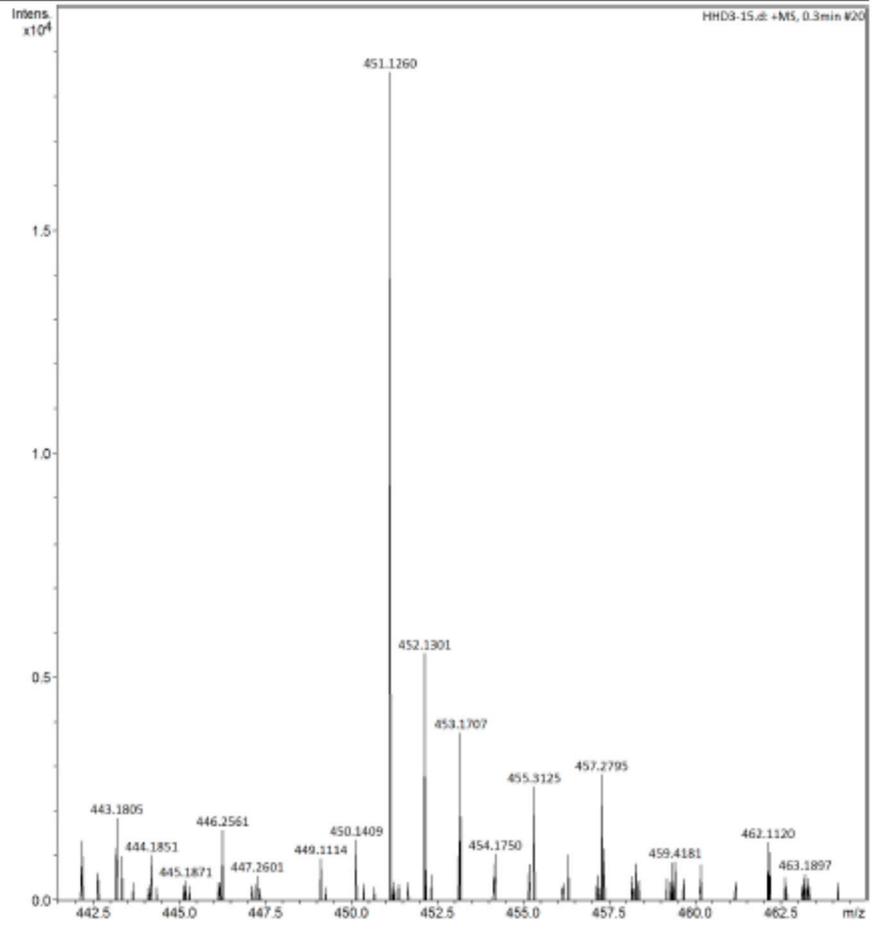

HHD3-15.d

Bruker Compass DataAnalysis 4.2 printed: 2021/1/25 20:50:42 by: ZMH Page 1 of 1

Figure S8. HR-ESI-MS spectrum of compound **1**

Thermo Scientific ~ VISIONpro SOFTWARE V4.41

Operator Name	(None Entered)	Date of Report	2021/1/22
Department	(None Entered)	Time of Report	21:29:52下午
Organization	(None Entered)		
Information	(None Entered)		

Scan Graph

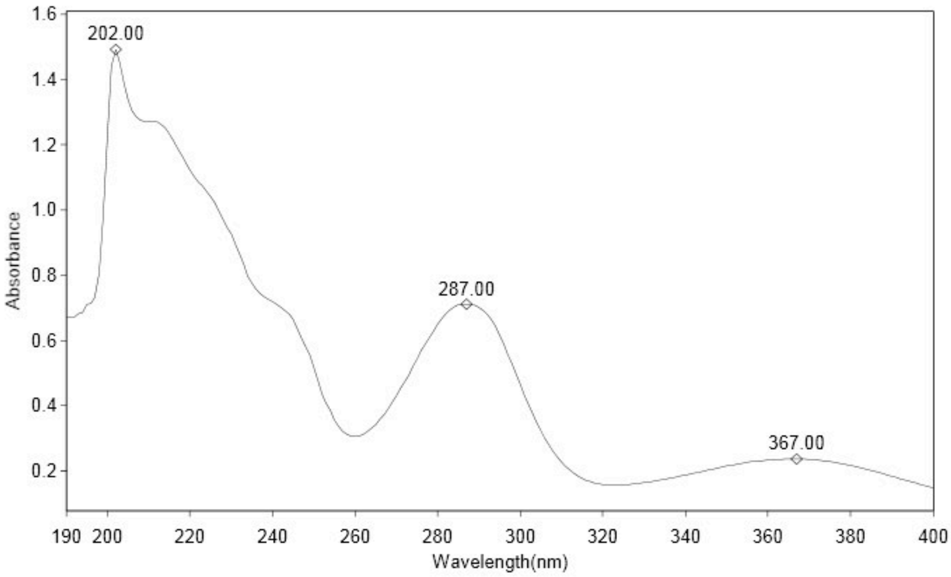

Results Table - scan006,HHD-3-15,Cycle01

nm	A	**Peak Pick Method**
202.00	1.492	Find 8 Peaks Above -3.0000 A
287.00	.712	Start Wavelength190.00 nm
367.00	.238	Stop Wavelength400.00 nm
		Sort By Wavelength
Sensitivity	Medium	

Page 1, Scan Graph

Figure S9. UV spectrum of **1**

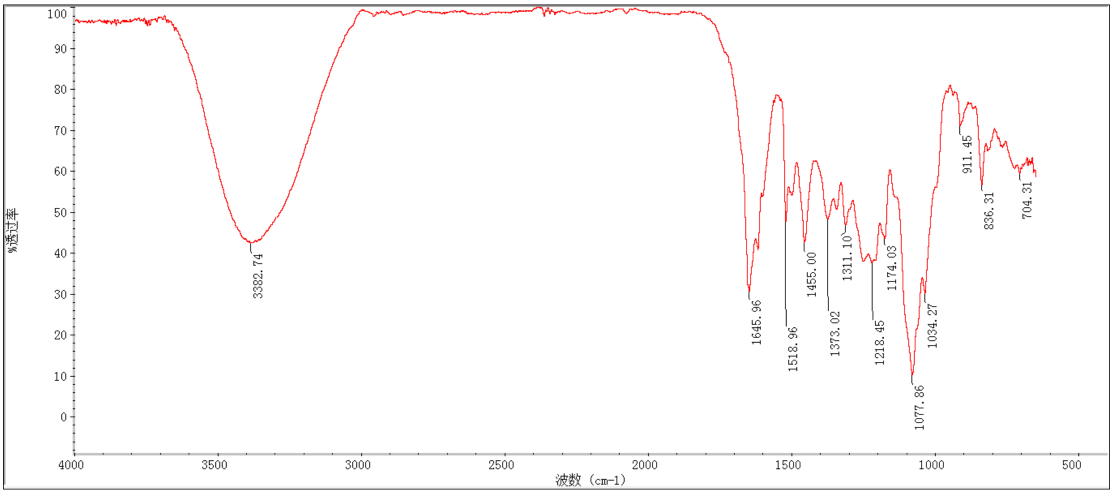

Figure S10. IR spectrum of **1**

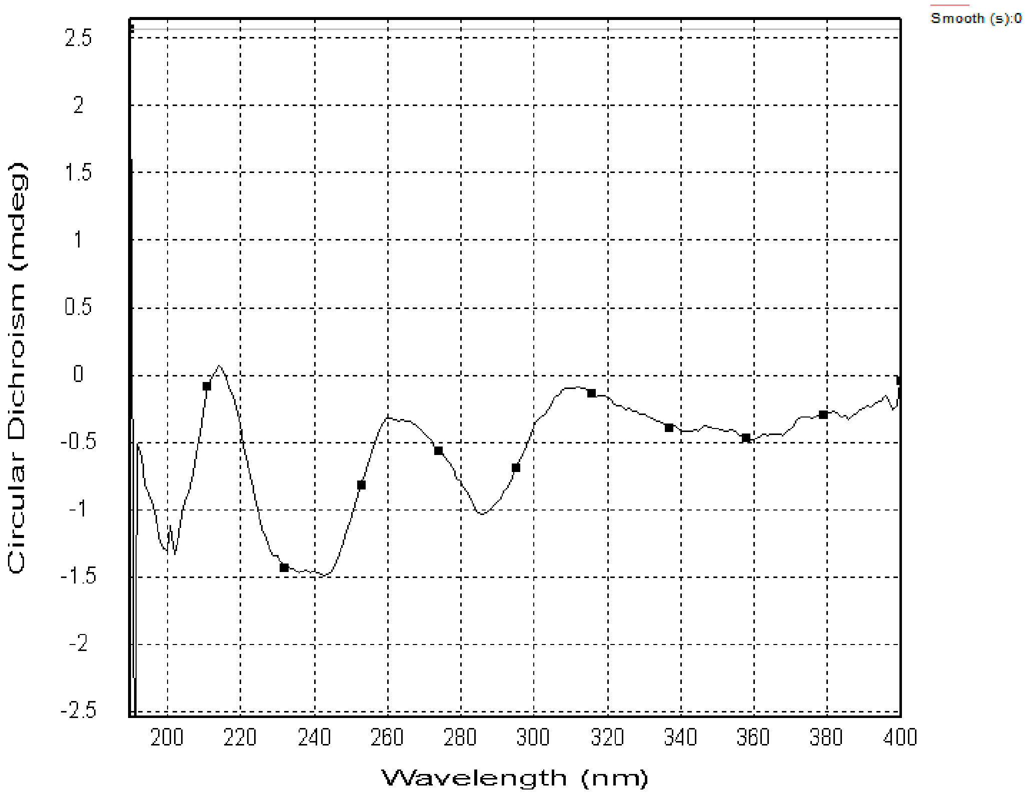

Figure S11. CD spectrum of **1**

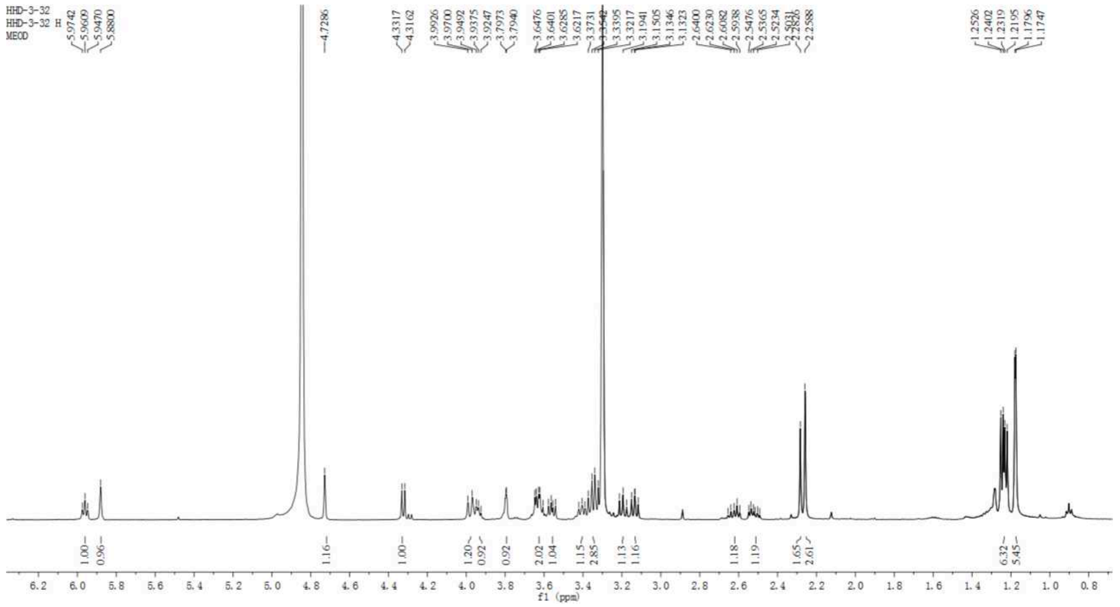

Figure S12. ^{1}H NMR spectrum (500MHz, CD_3OD) of **2**

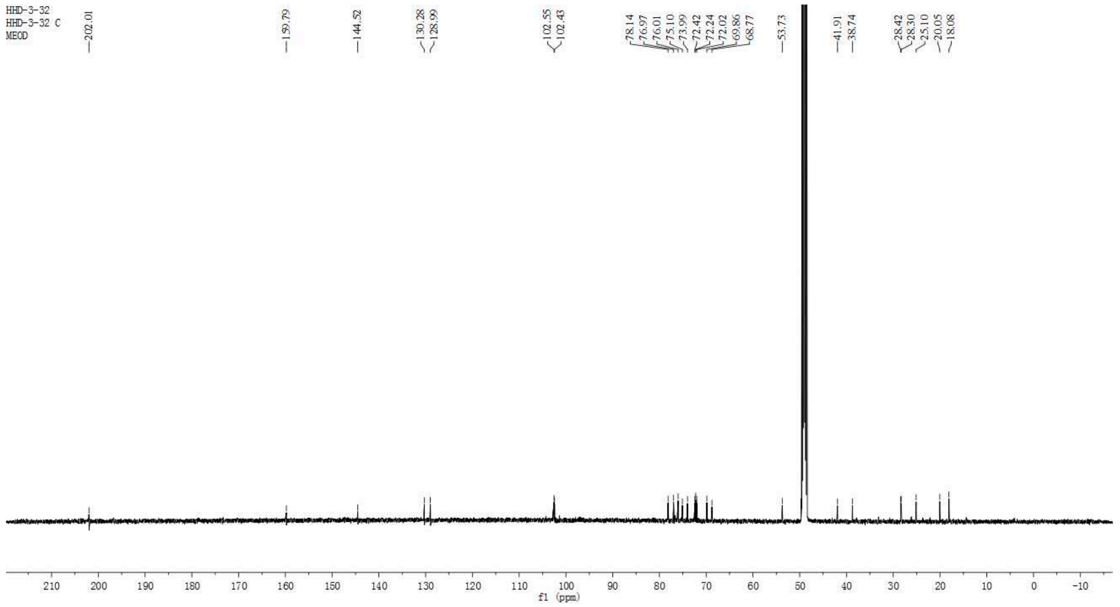

Figure S13. ^{13}C NMR spectrum (125MHz, CD_3OD) of **2**

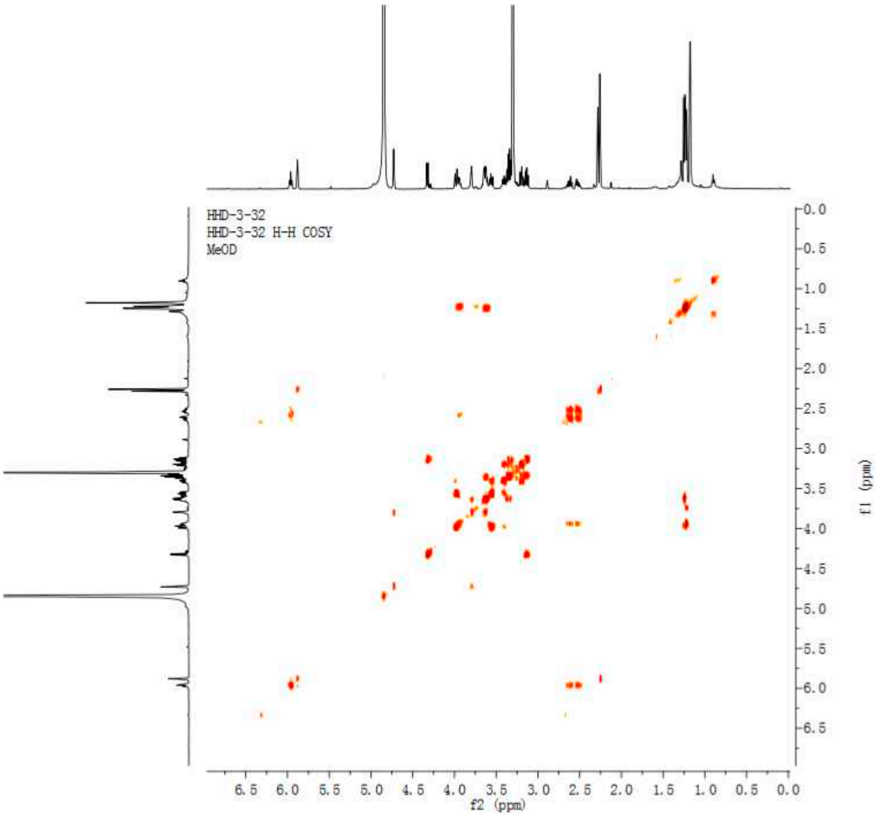

Figure S14. ^{1}H-^{1}H COSY spectrum of **2**

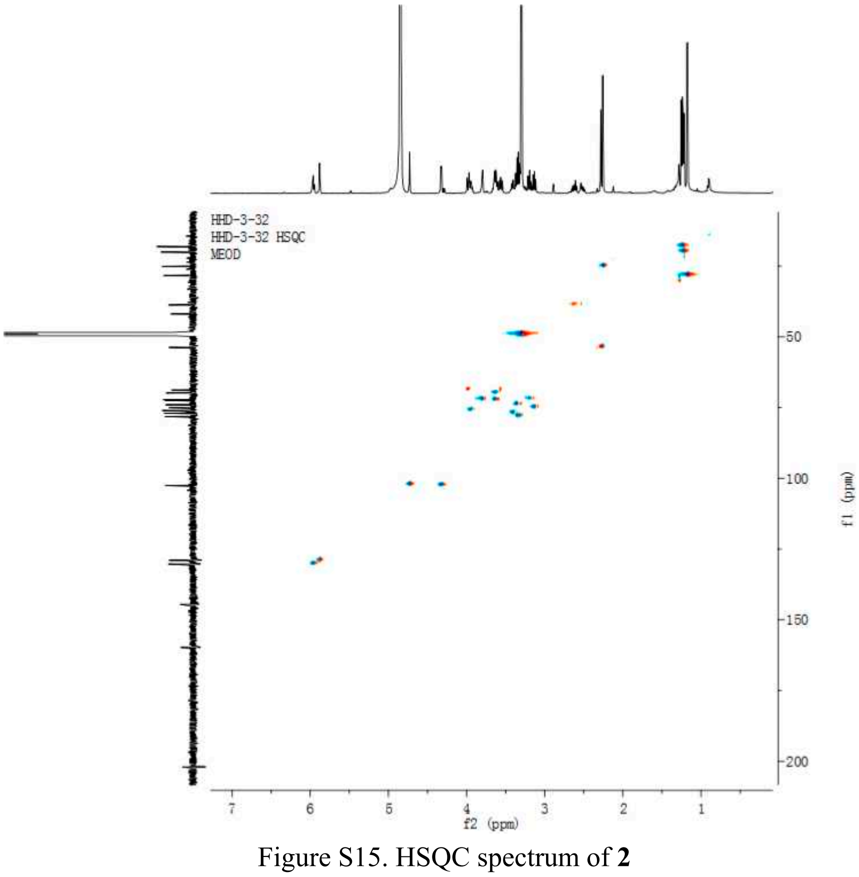

Figure S15. HSQC spectrum of **2**

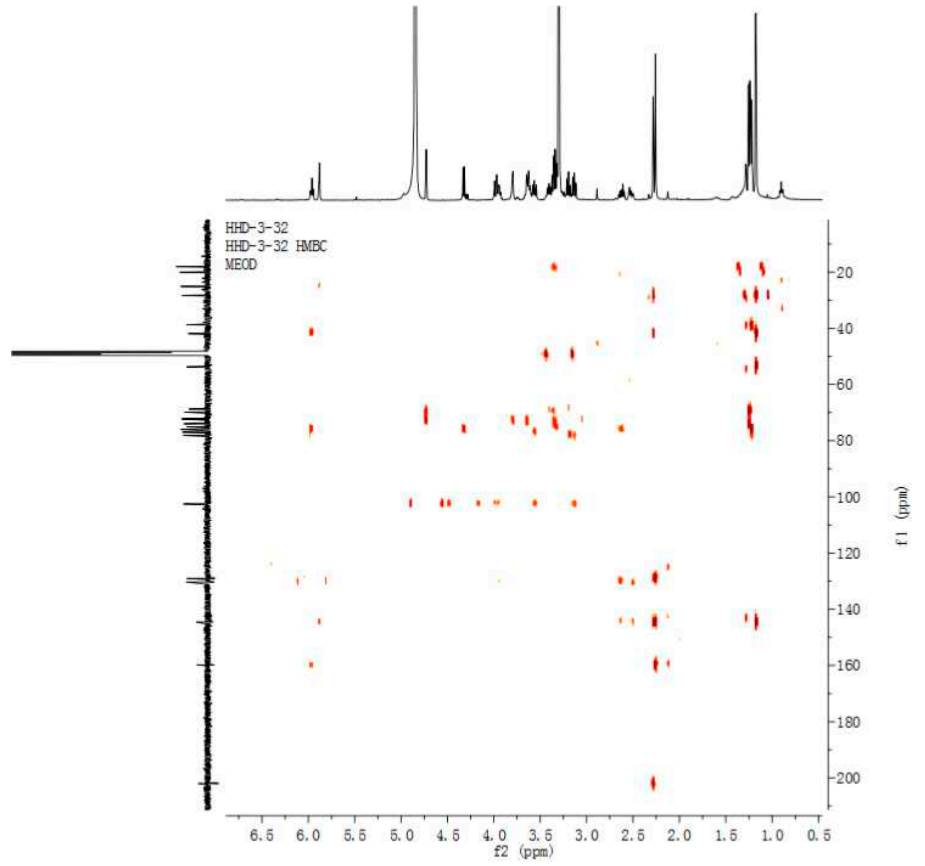

Figure S16. HMBC spectrum of **2**

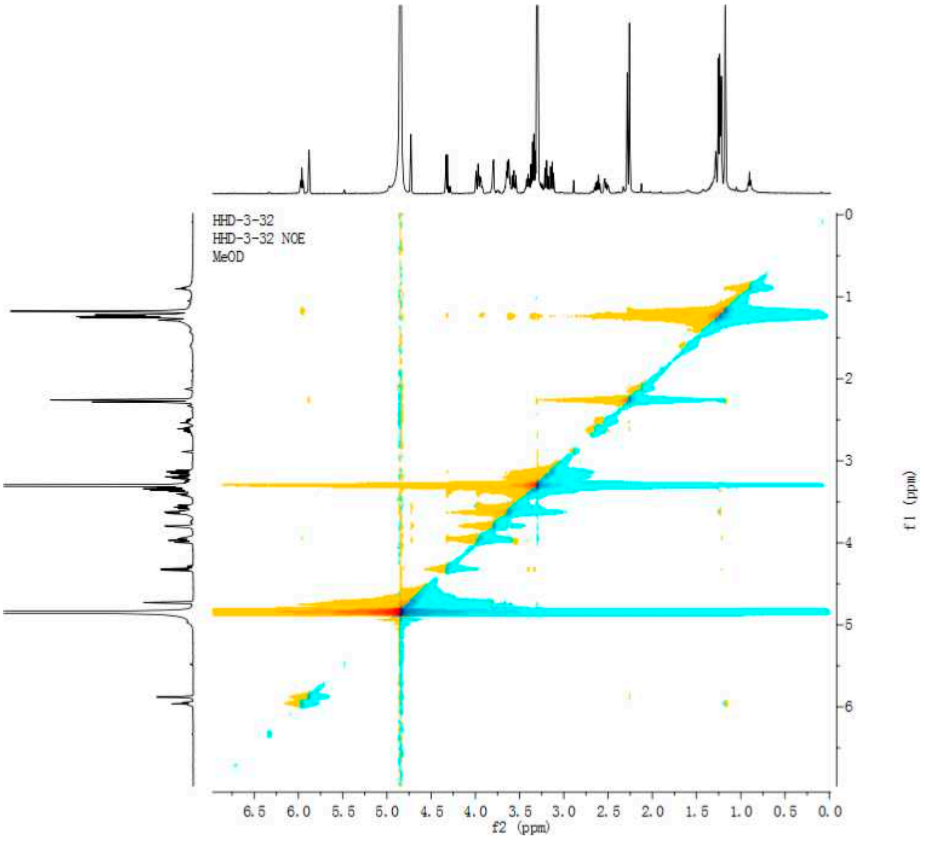

Figure S17.NOESY spectrum of **2**

Display Report

Analysis Info

Analysis Name	\\ESI-PC\data-D\Data\GJH\WMN\20201126\HHD-3-32.d	Acquisition Date	2020/11/27 21:20:37
Method	tune_pos_standard_20141031.m	Operator	Demo User
Sample Name	HHD-3-32	Instrument	maXis HD 1820881.21303
Comment			

Acquisition Parameter

Source Type	ESI	Ion Polarity	Positive	Set Nebulizer	0.3 Bar
Focus	Active	Set Capillary	3500 V	Set Dry Heater	200 ¡ãC
Scan Begin	50 m/z	Set End Plate Offset	-500 V	Set Dry Gas	4.0 l/min
Scan End	3000 m/z	Set Charging Voltage	2000 V	Set Divert Valve	Waste
		Set Corona	0 nA	Set APCI Heater	0 ¡ãC

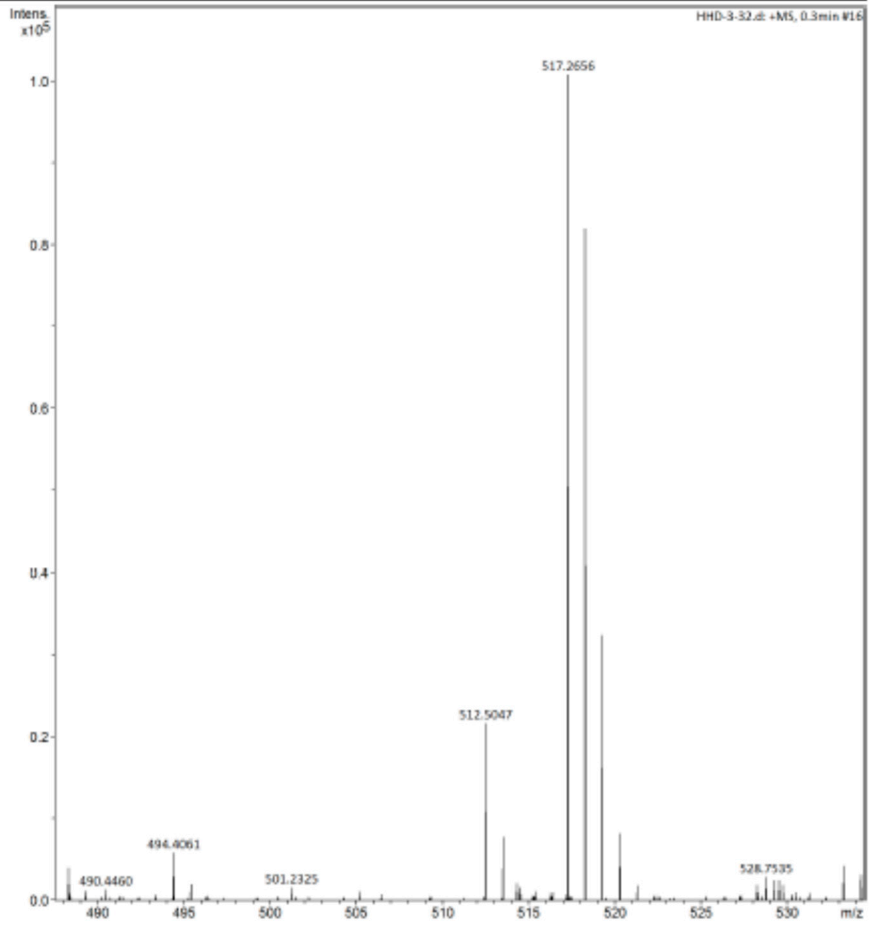

Figure S18. HR-ESI-MS spectrum of compound **2**

Thermo Scientific ~ VISIONpro SOFTWARE V4.41

Operator Name	(None Entered)	Date of Report	2021/1/22
Department	(None Entered)	Time of Report	21:49:37下午
Organization	(None Entered)		
Information	(None Entered)		

Scan Graph

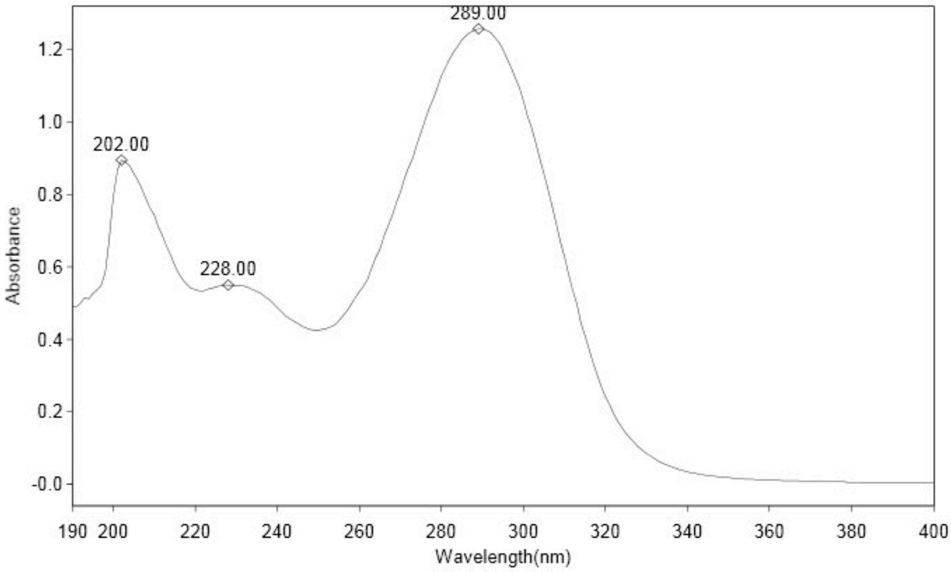

Results Table - HHD-3-32.sre,HHD-3-32,Cycle01

nm	A	**Peak Pick Method**
202.00	.894	Find 8 Peaks Above -3.0000 A
228.00	.550	Start Wavelength190.00 nm
289.00	1.257	Stop Wavelength400.00 nm
		Sort By Wavelength
Sensitivity	Very Low	

Page 1, Scan Graph

Figure S19. UV spectrum of **2**

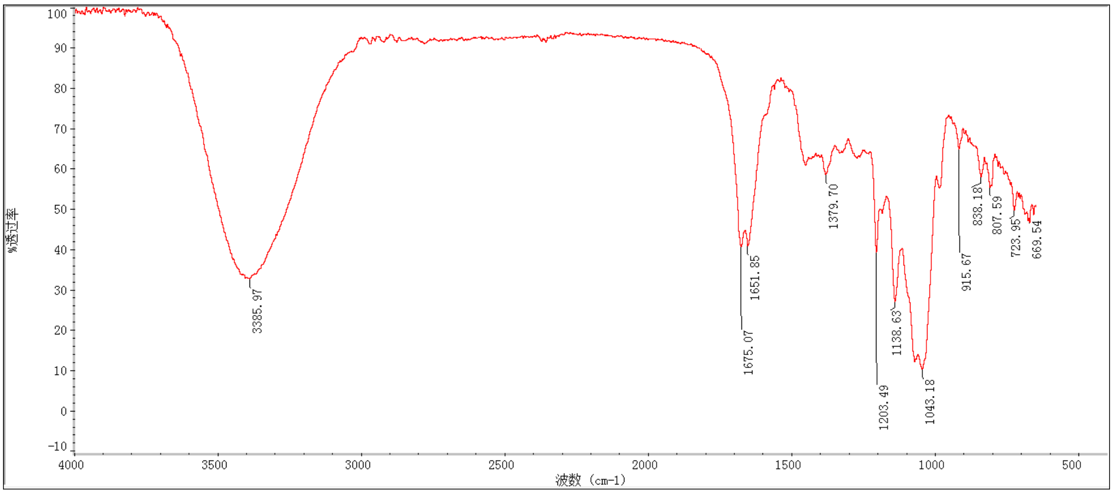
Figure S20. IR spectrum of **2**

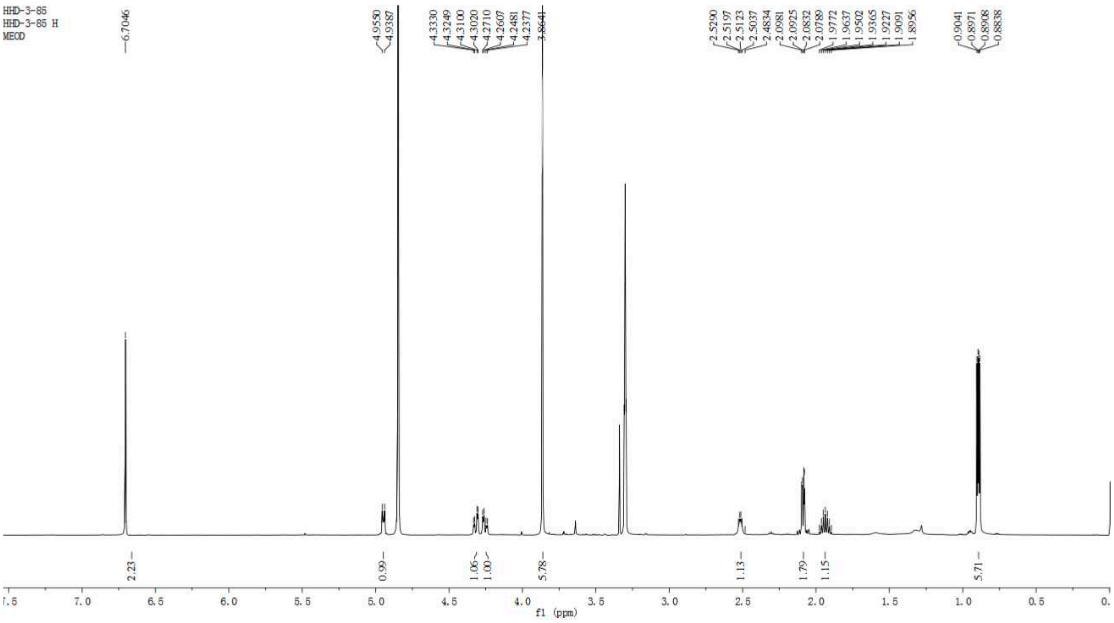
Figure S21. ^{1}H NMR spectrum (500MHz, CD_3OD) of **3**

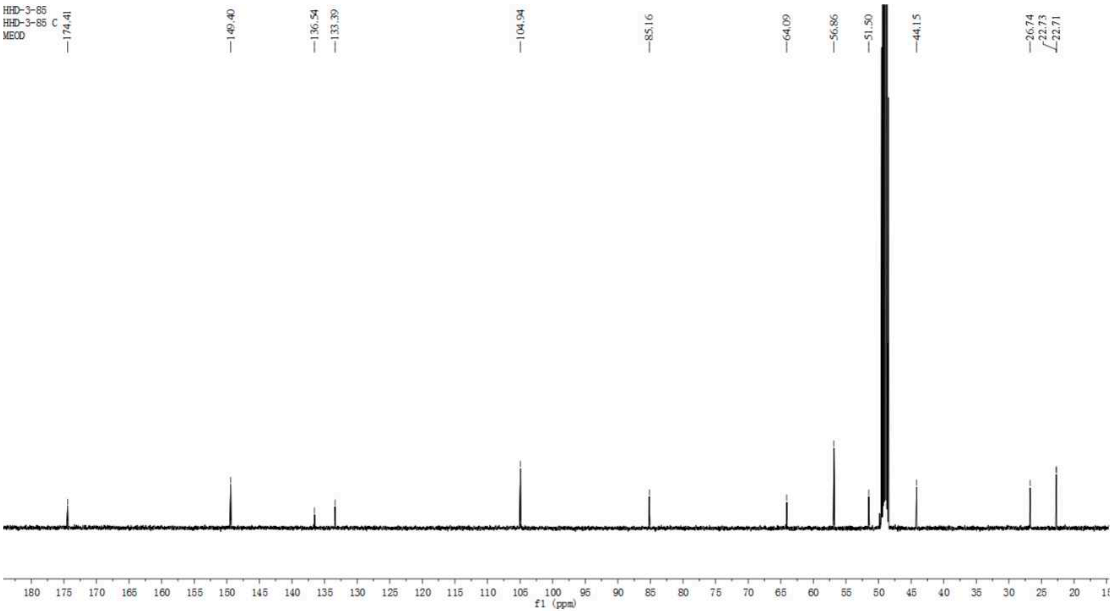

Figure S22. ^{13}C NMR spectrum (125MHz, CD_3OD) of **3**

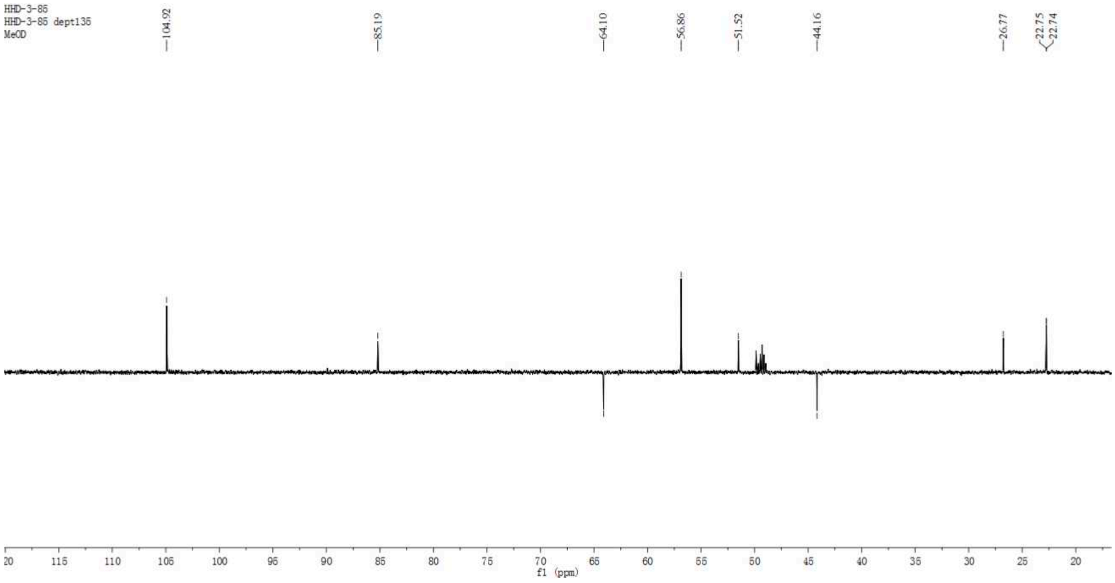

Figure S23. DEPT135 spectrum of **3**

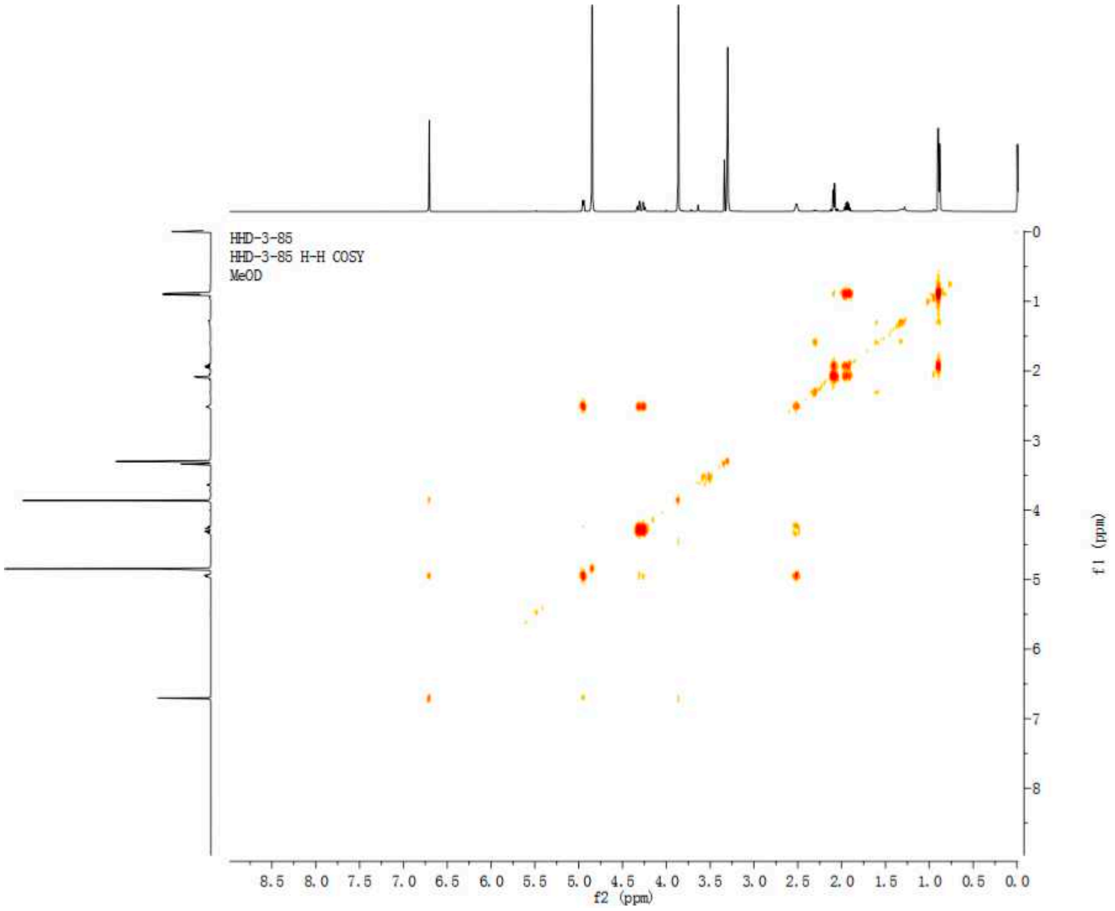

Figure S24. ^1H-^1H COSY spectrum of **3**

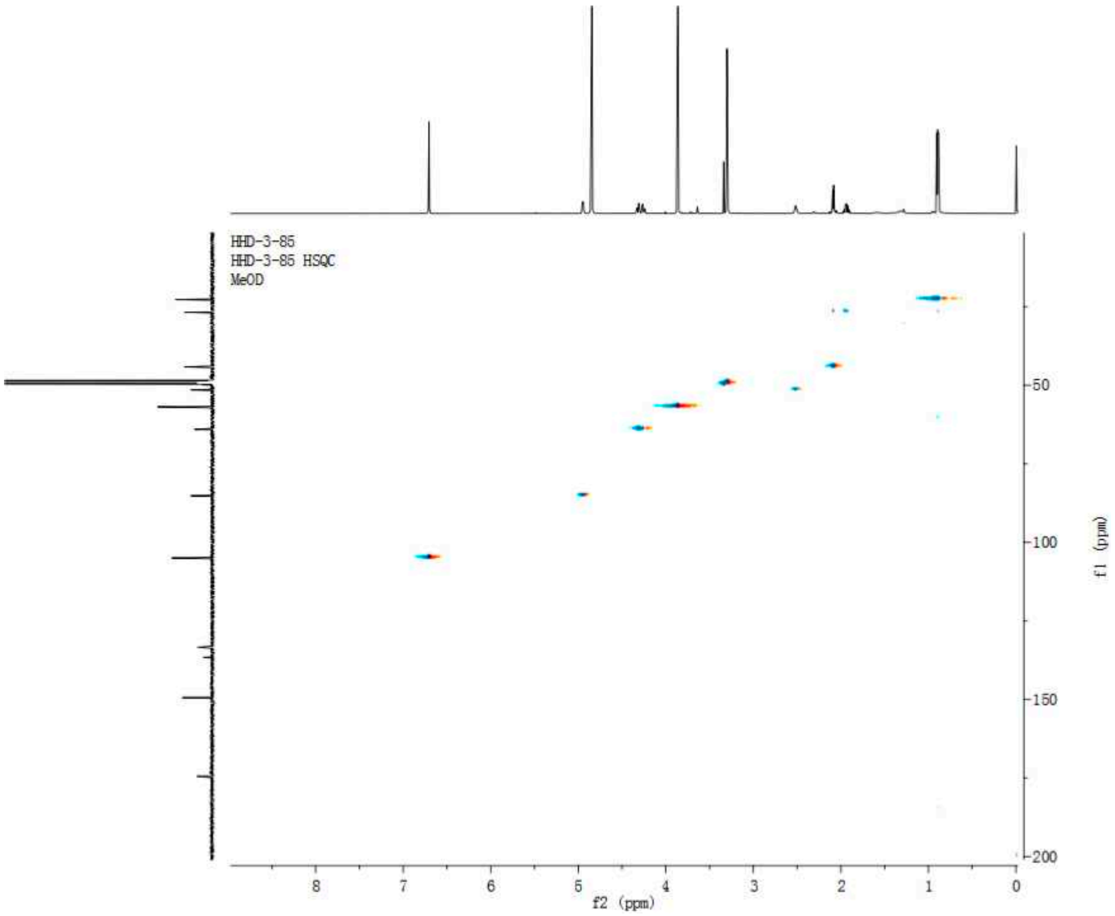

Figure S25. HSQC spectrum of **2**

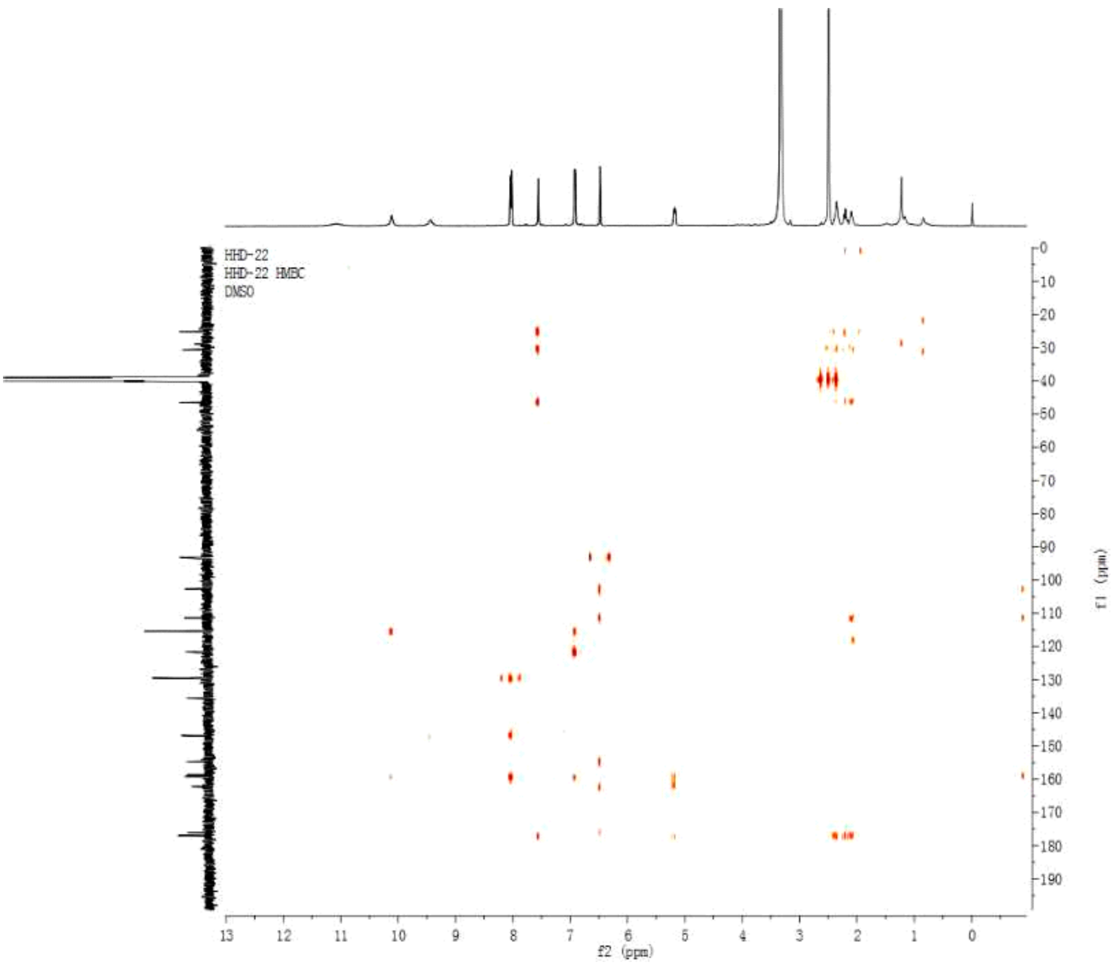

Figure S26. HMBC spectrum of **3**

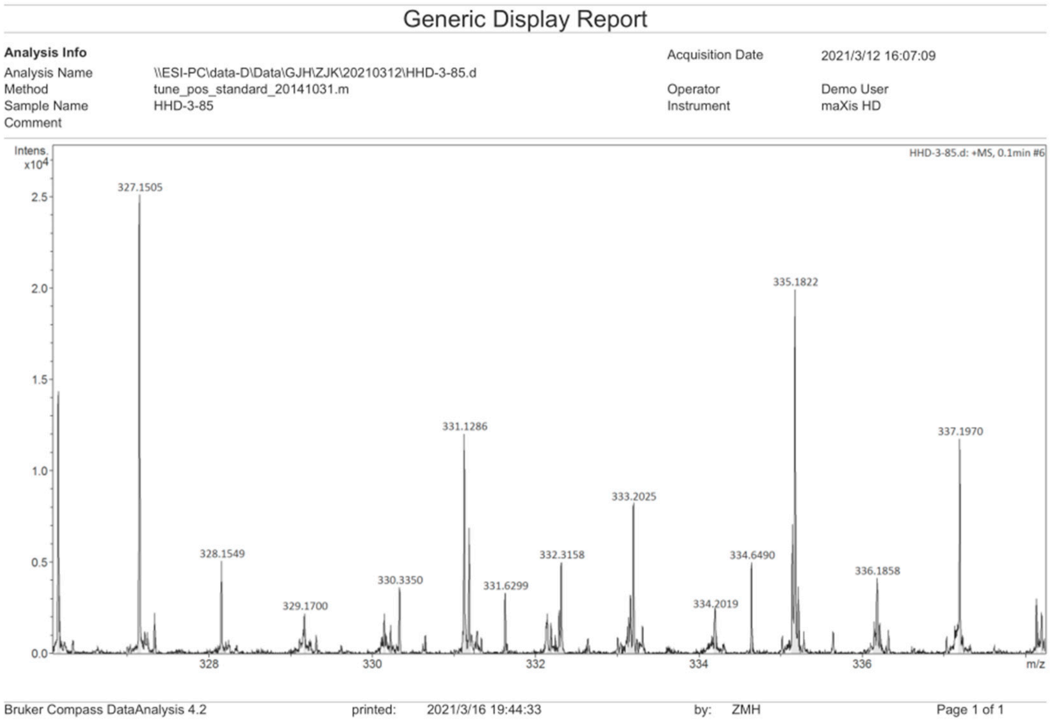

Figure S27. HR-ESI-MS spectrum of compound **3**

Thermo Scientific ~ VISIONpro SOFTWARE V4.41

Operator Name	(None Entered)	Date of Report	2021/1/22
Department	(None Entered)	Time of Report	21:43:03下午
Organization	(None Entered)		
Information	(None Entered)		

Scan Graph

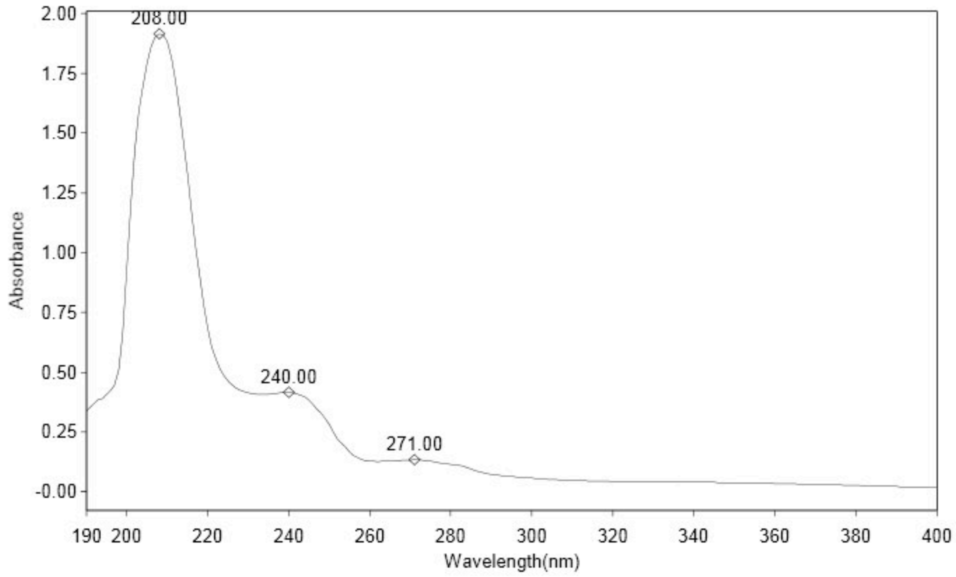

Results Table - scan010,HHD-3-85,Cycle01

nm	A	**Peak Pick Method**
208.00	1.916	Find 8 Peaks Above -3.0000 A
240.00	.417	Start Wavelength190.00 nm
271.00	.134	Stop Wavelength400.00 nm
		Sort By Wavelength
Sensitivity	High	

Figure S28. UV spectrum of **3**

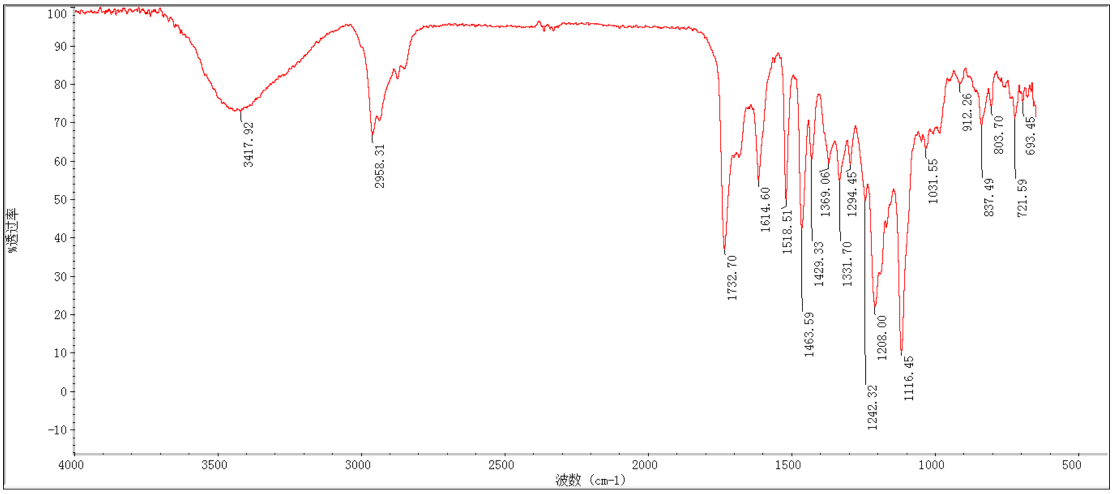

Figure S29. IR spectrum of **3**

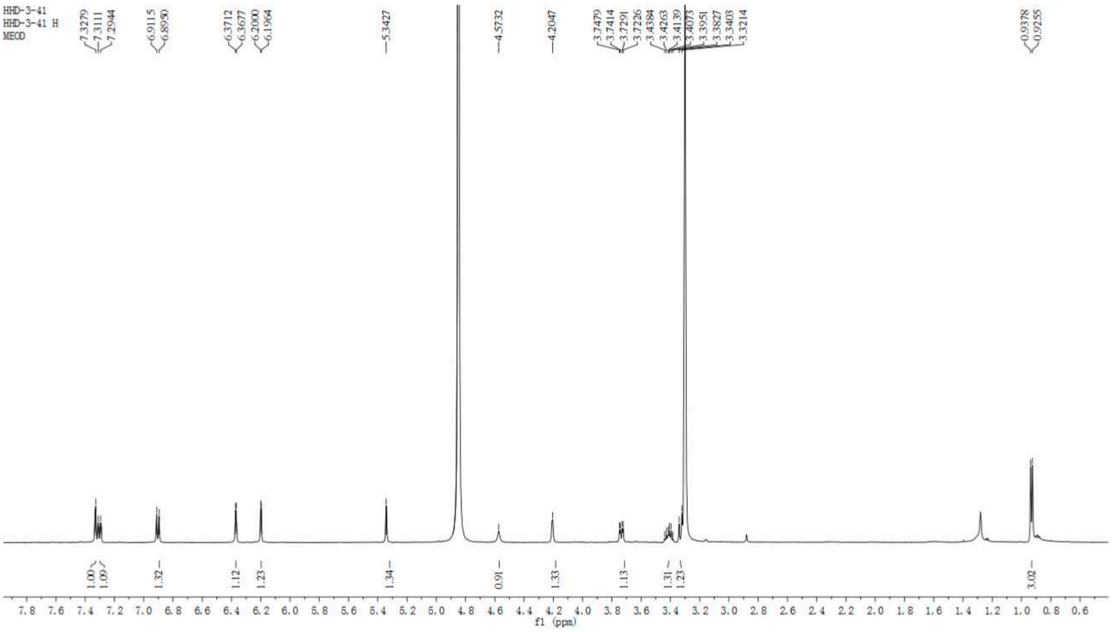

Figure S30. ^{1}H NMR spectrum (500MHz, CD_3OD) of **4**

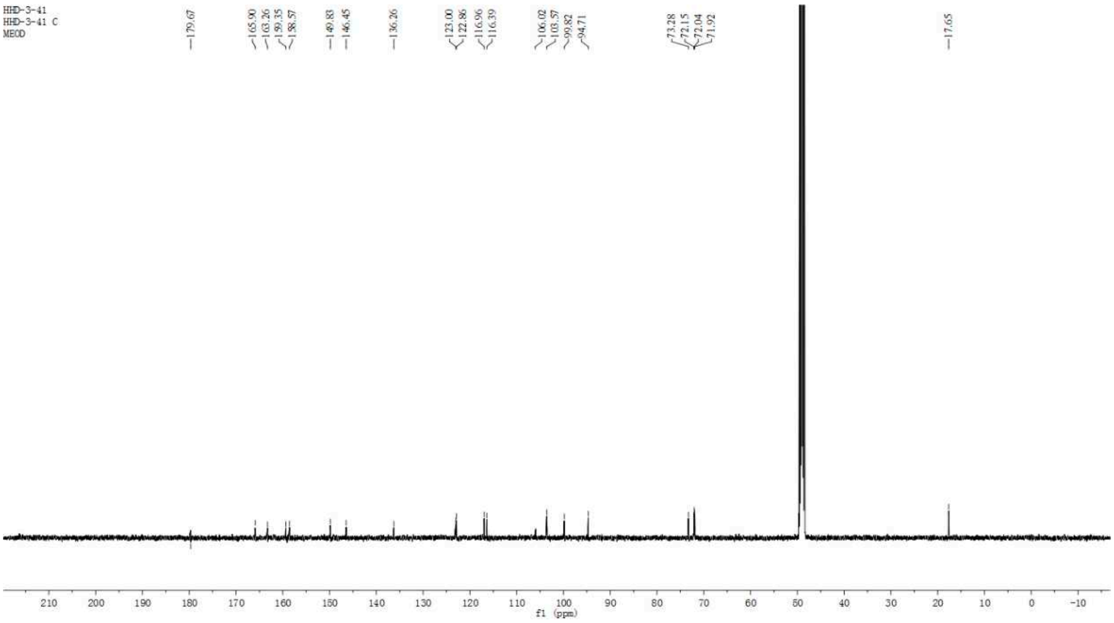

Figure S31. ^{13}C NMR spectrum (125MHz, CD_3OD) of **4**

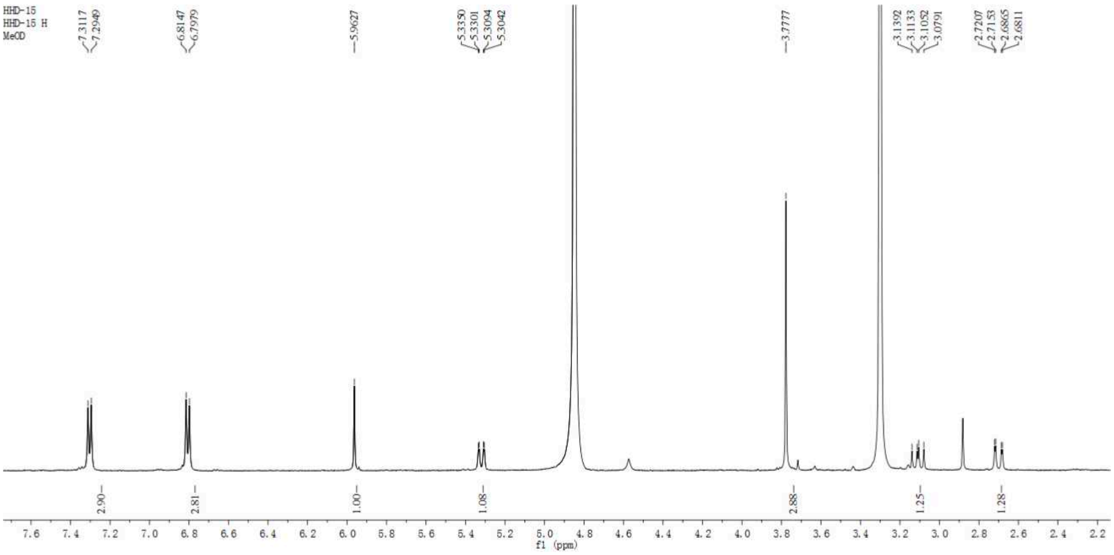

Figure S32. ^{1}H NMR spectrum (500MHz, CD_3OD) of **5**

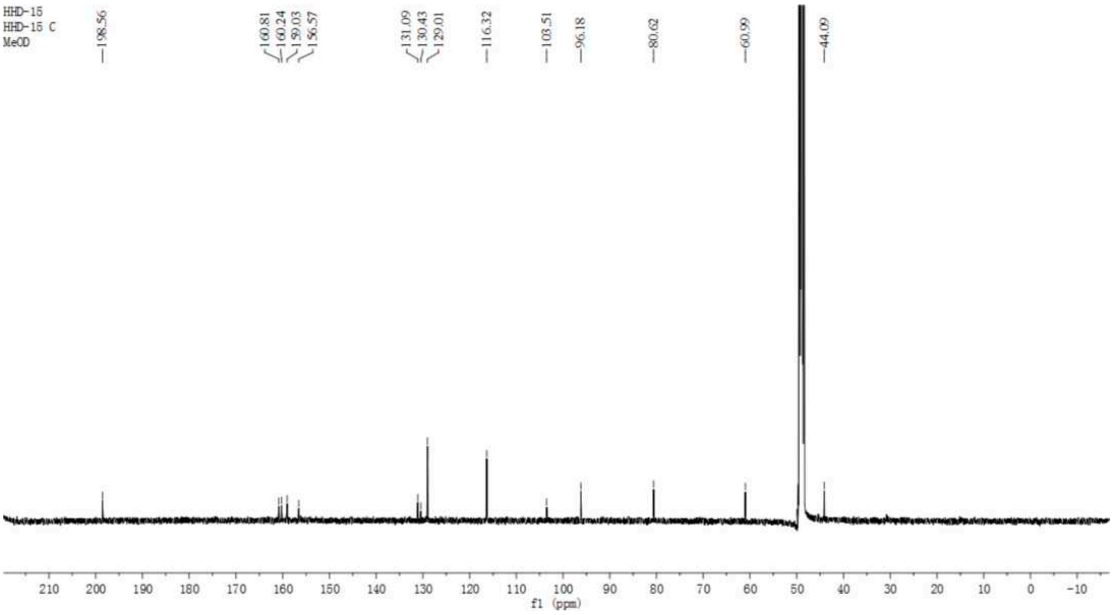

Figure S33. ^{13}C NMR spectrum (125MHz, CD_3OD) of **5**

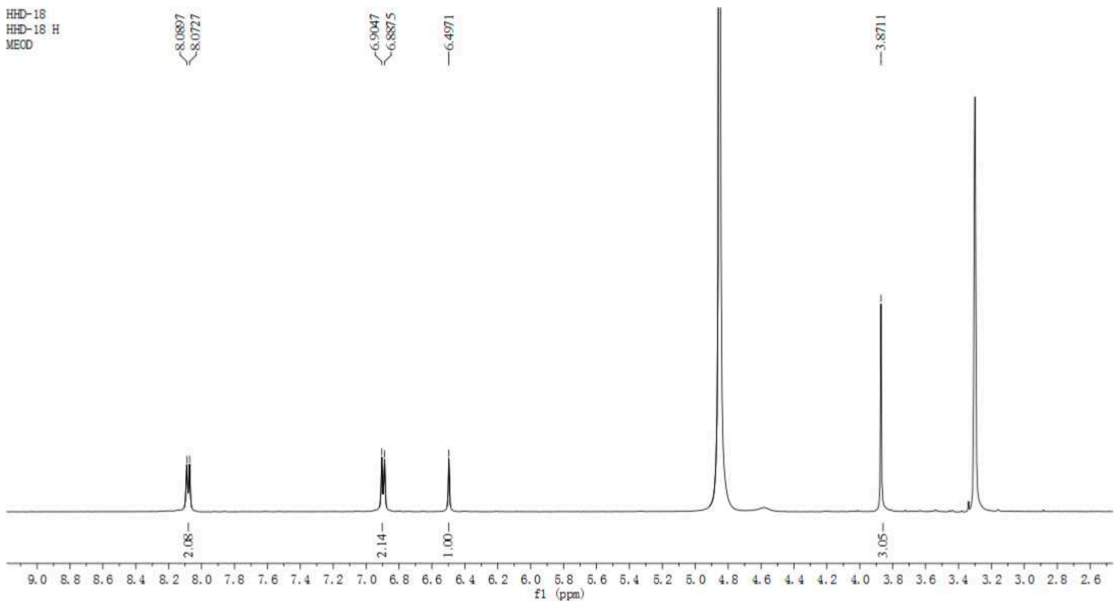

Figure S34. ^{1}H NMR spectrum (500MHz, CD_3OD) of **6**

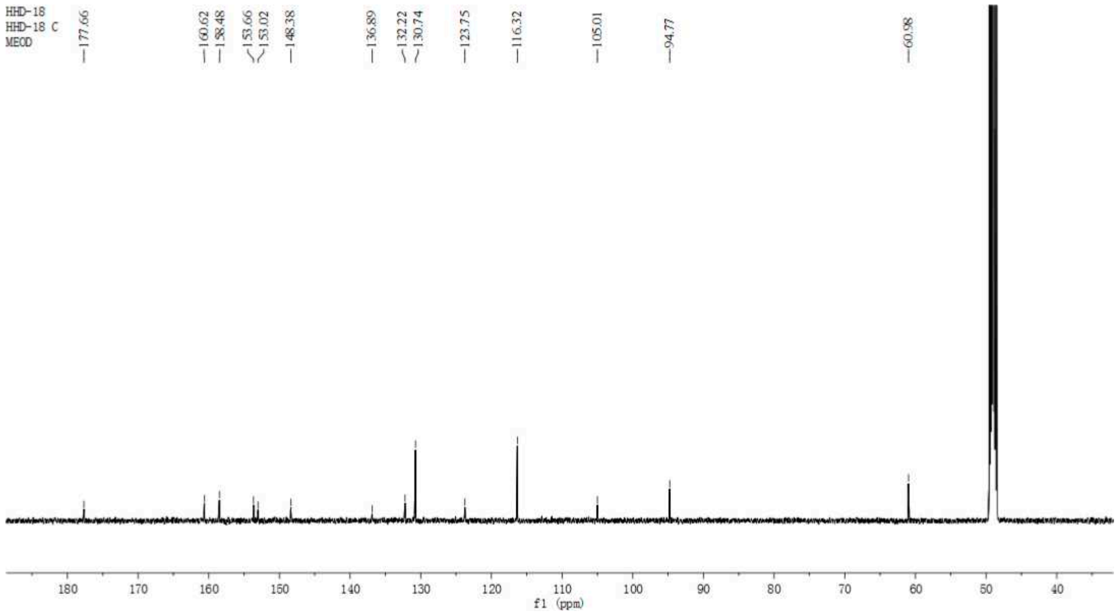

Figure S35. ^{13}C NMR spectrum (125MHz, CD_3OD) of **6**

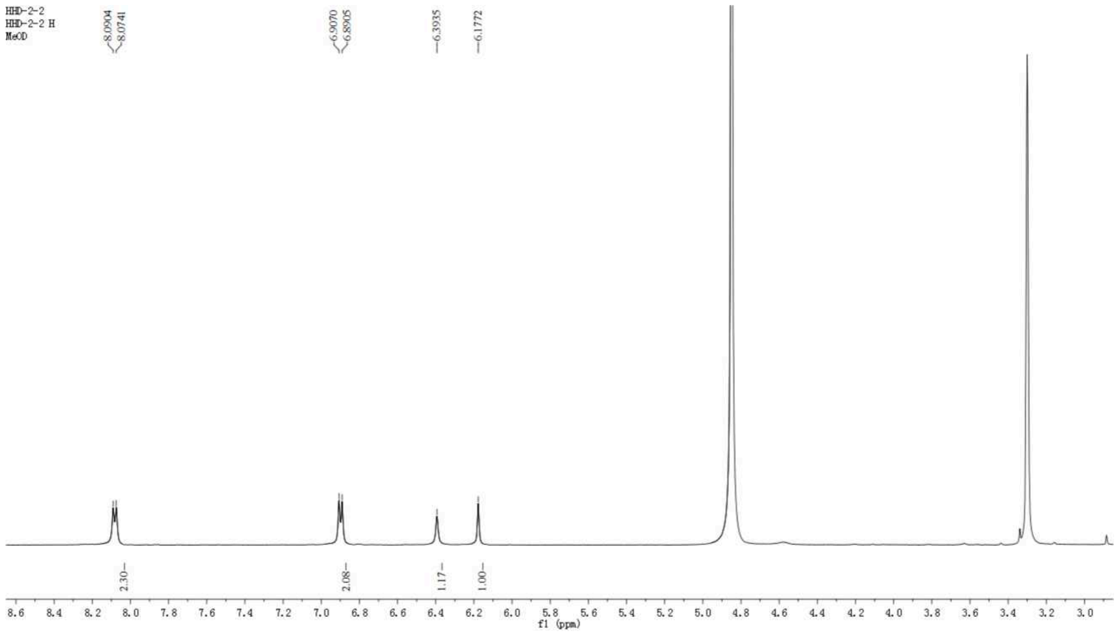

Figure S36. ^{1}H NMR spectrum (500MHz, CD_3OD) of **7**

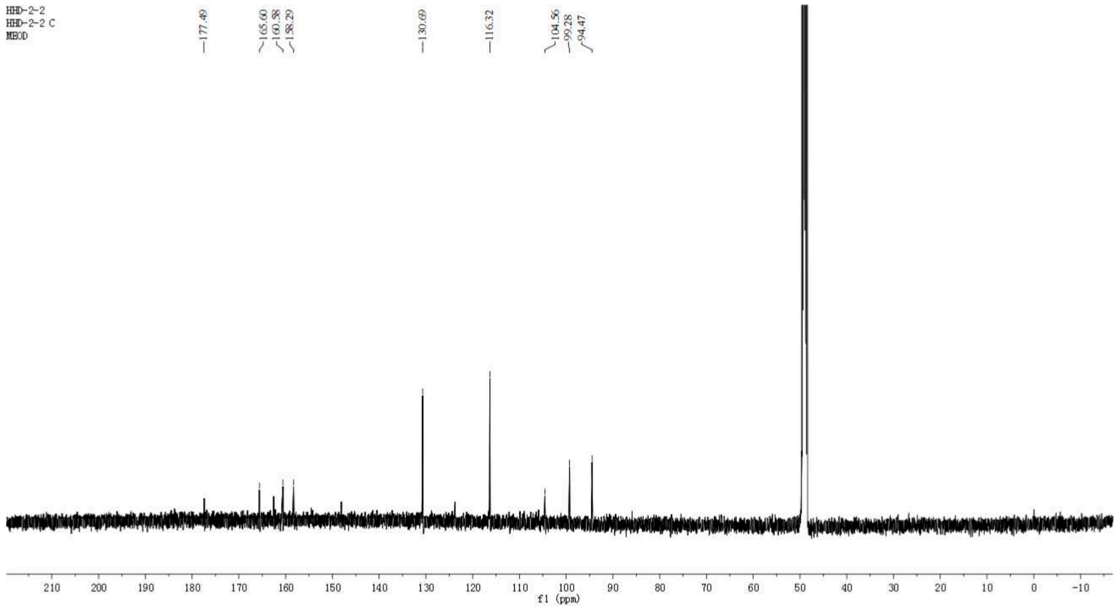

Figure S37. ^{13}C NMR spectrum (125MHz, CD_3OD) of **7**

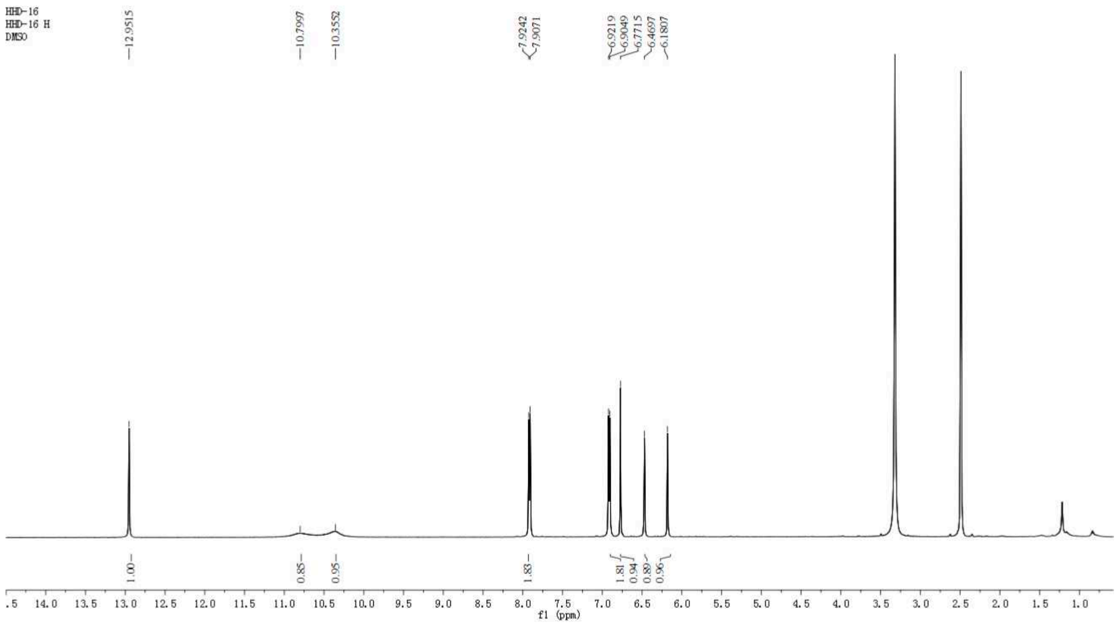

Figure S38. ^{1}H NMR spectrum (500MHz, DMSO) of **8**

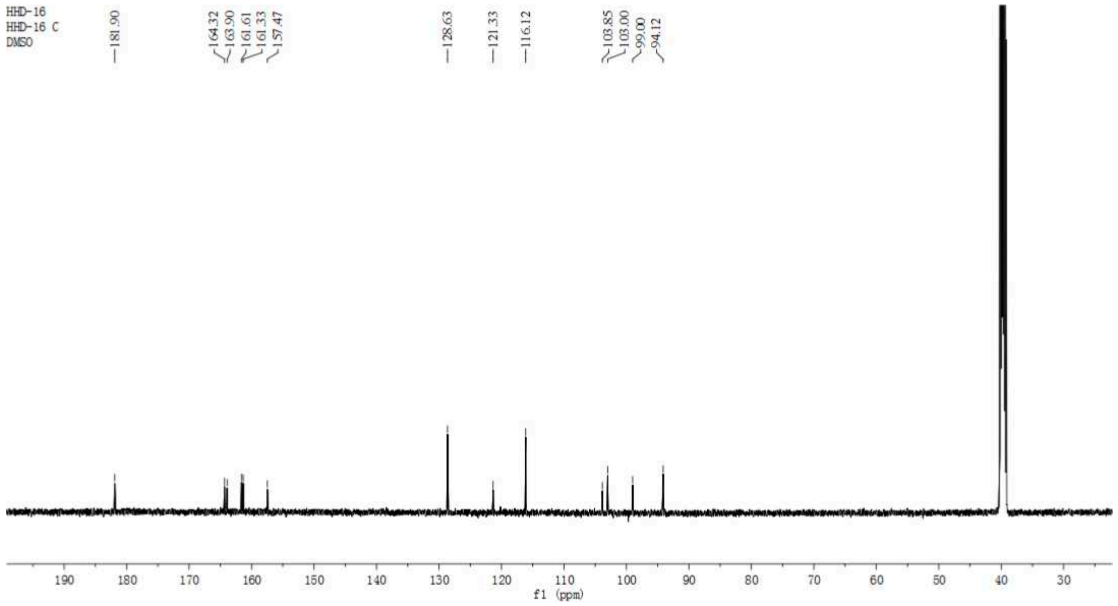

Figure S39. ^{13}C NMR spectrum (125MHz, DMSO) of **8**

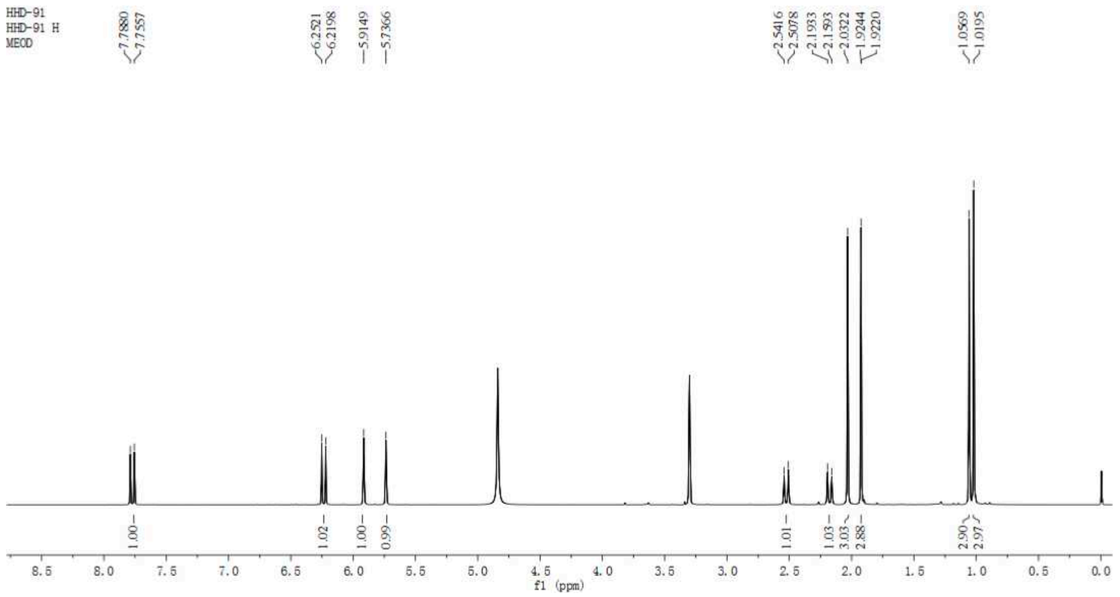

Figure S40. ^{1}H NMR spectrum (500MHz, CD_3OD) of **9**

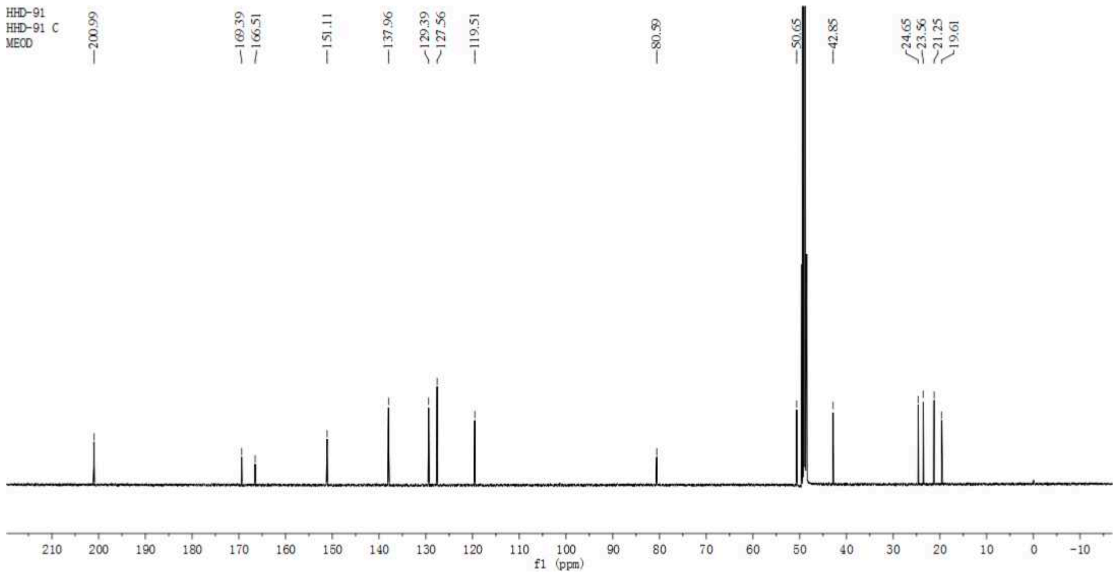

Figure S41. ^{13}C NMR spectrum (125MHz, CD_3OD) of **9**

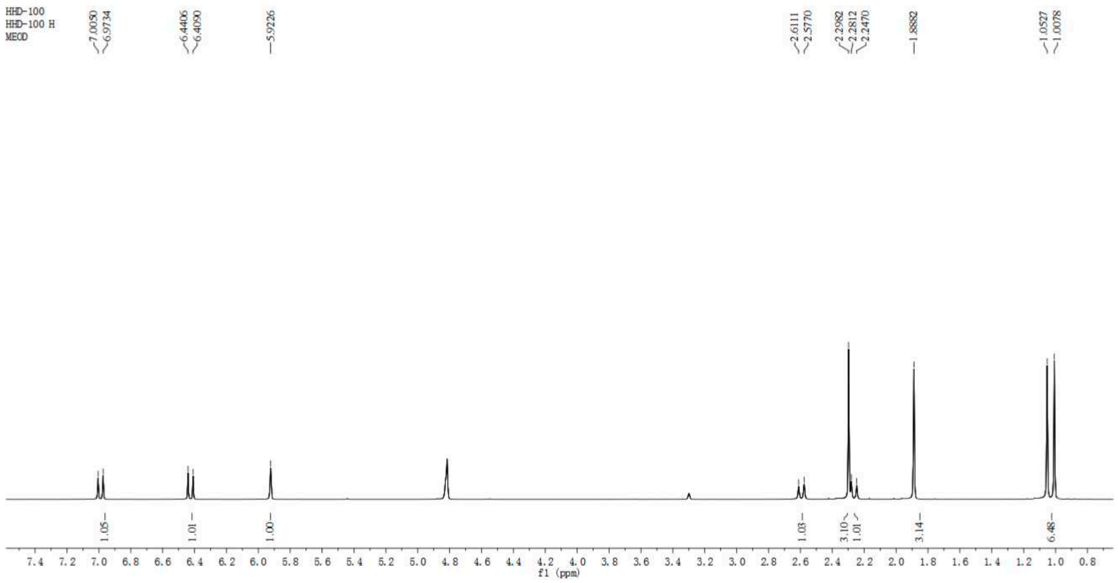

Figure S42. ^{1}H NMR spectrum (500MHz, CD_3OD) of **10**

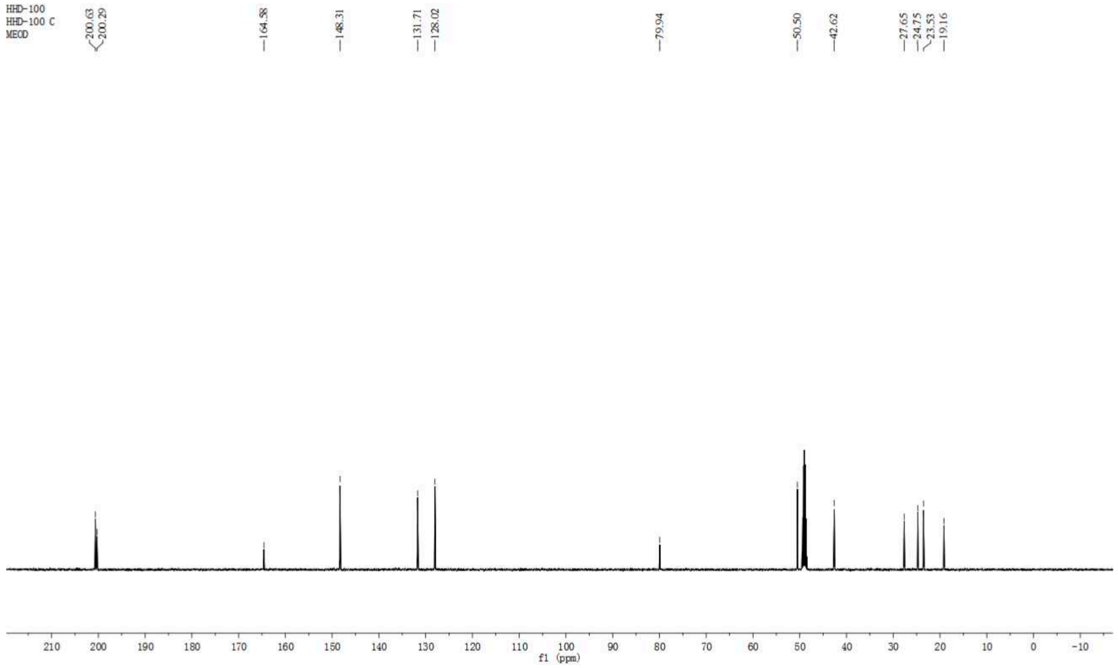

Figure S43. ^{13}C NMR spectrum (125MHz, CD_3OD) of **10**

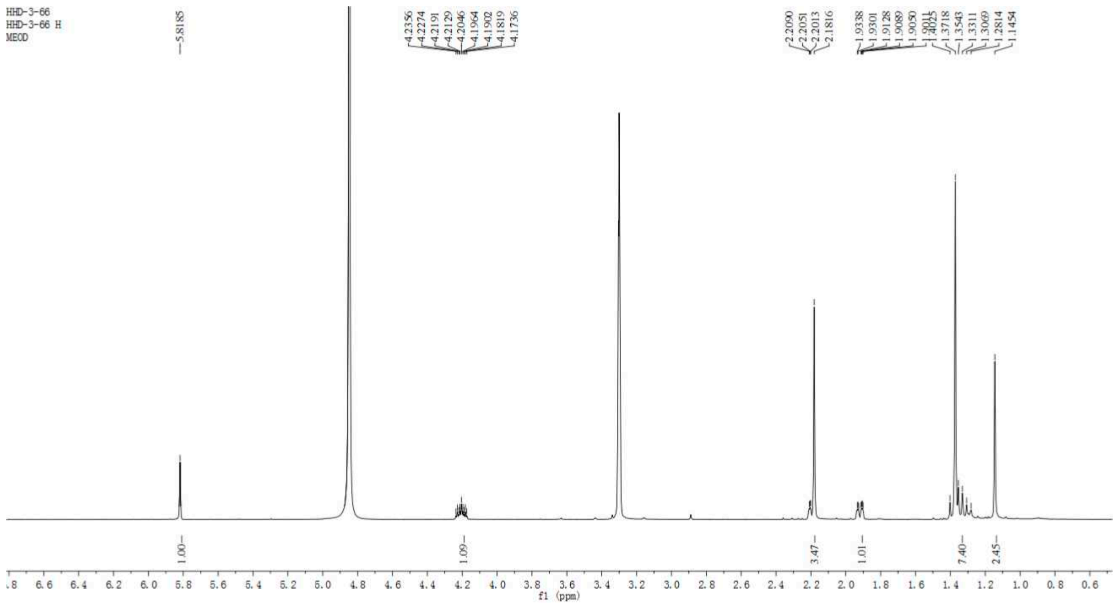

Figure S44. ^{1}H NMR spectrum (500MHz, CD_3OD) of **11**

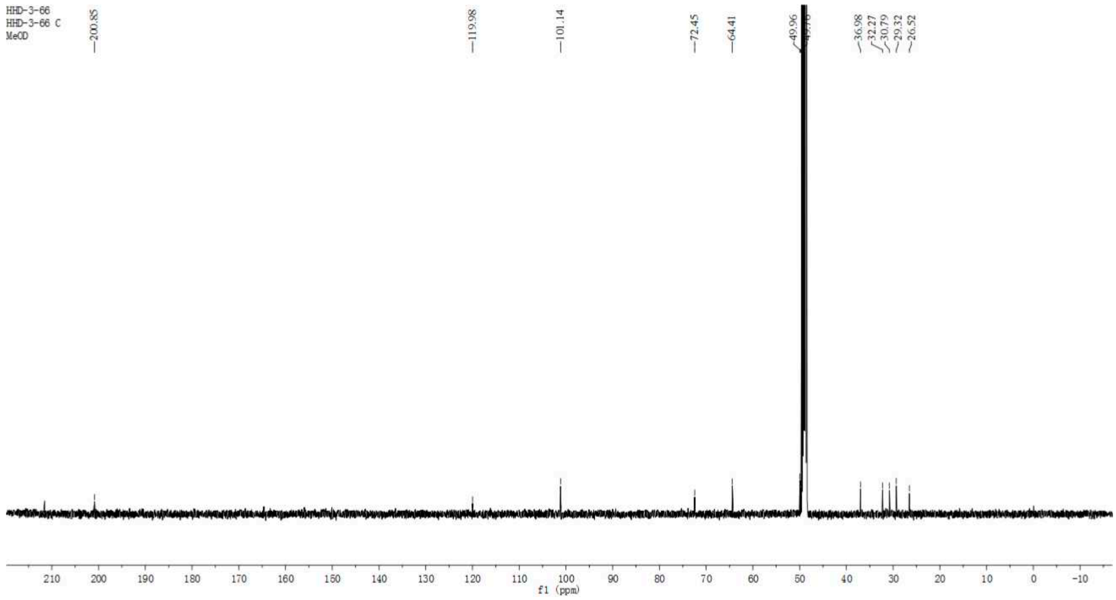

Figure S45. ^{13}C NMR spectrum (125MHz, CD_3OD) of **11**

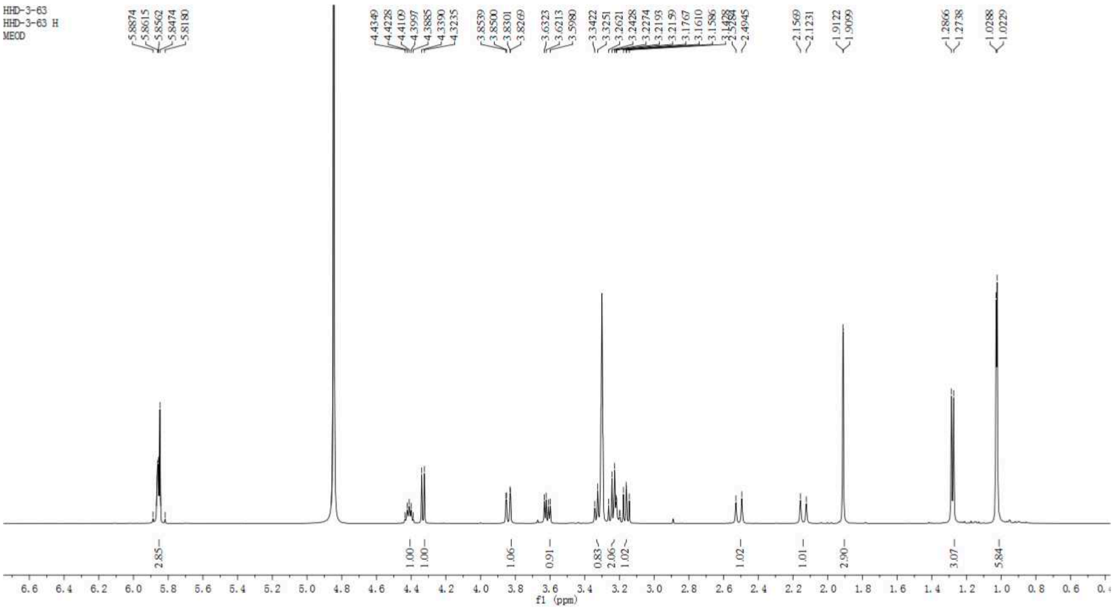

Figure S46. ^1H NMR spectrum (500MHz, CD_3OD) of **12**

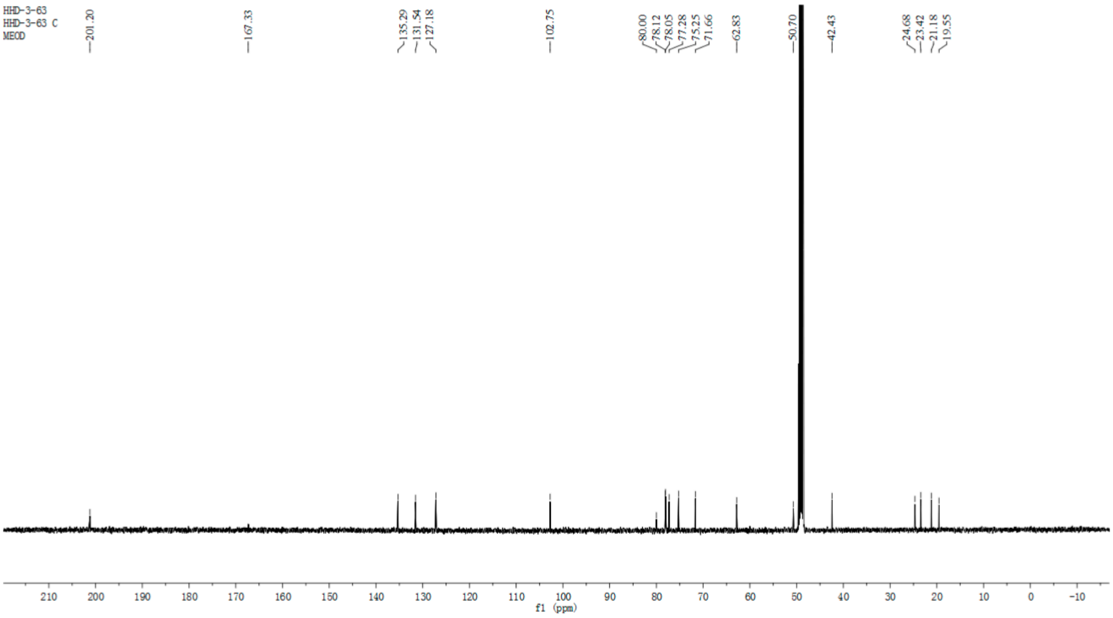

Figure S47. ^{13}C NMR spectrum (125MHz, CD_3OD) of **12**

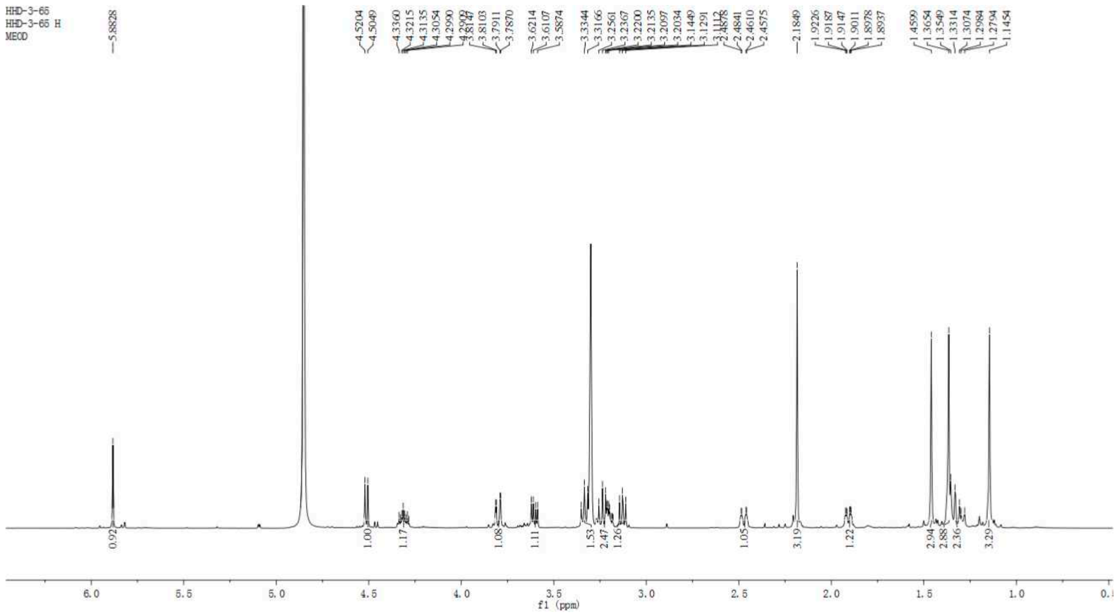

Figure S48. ^{1}H NMR spectrum (500MHz, CD_3OD) of **13**

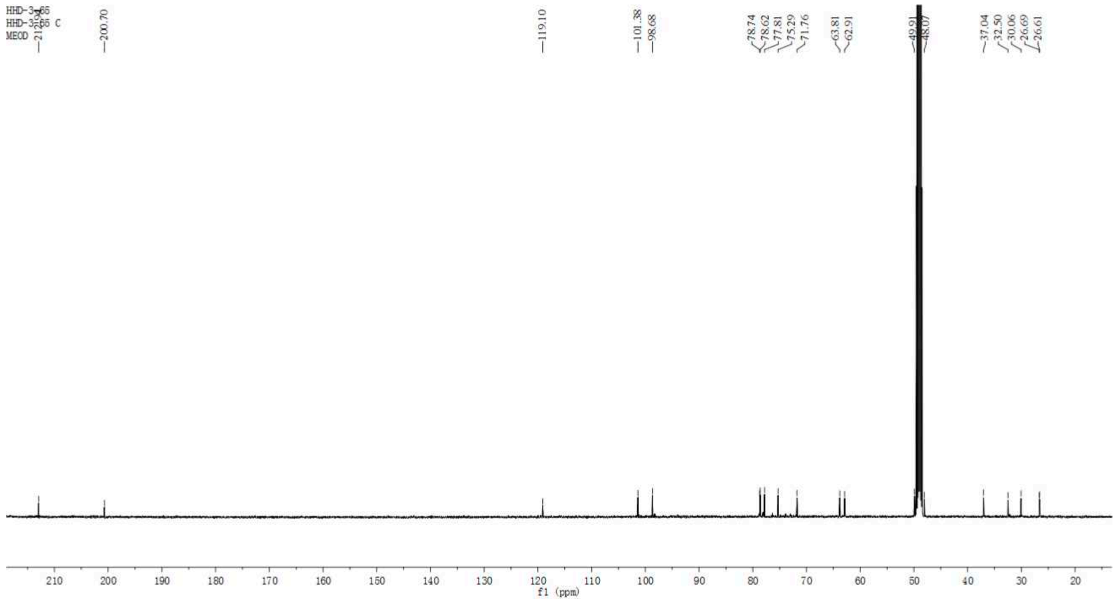

Figure S49. ^{13}C NMR spectrum (125MHz, CD_3OD) of **13**

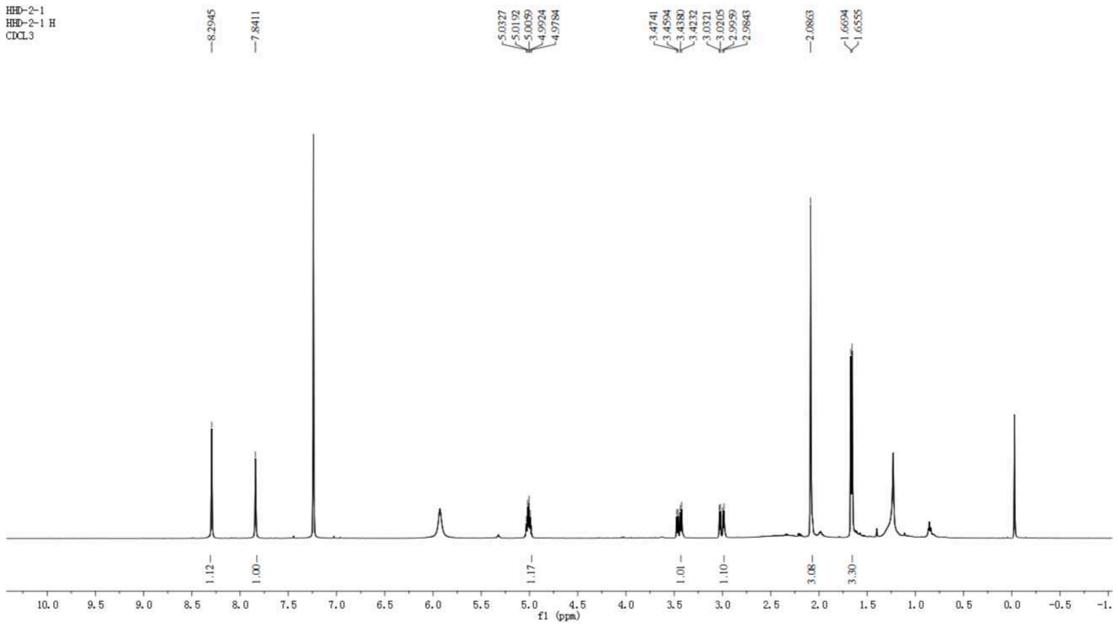

Figure S50. ^{1}H NMR spectrum (500MHz, CD_3OD) of **14**

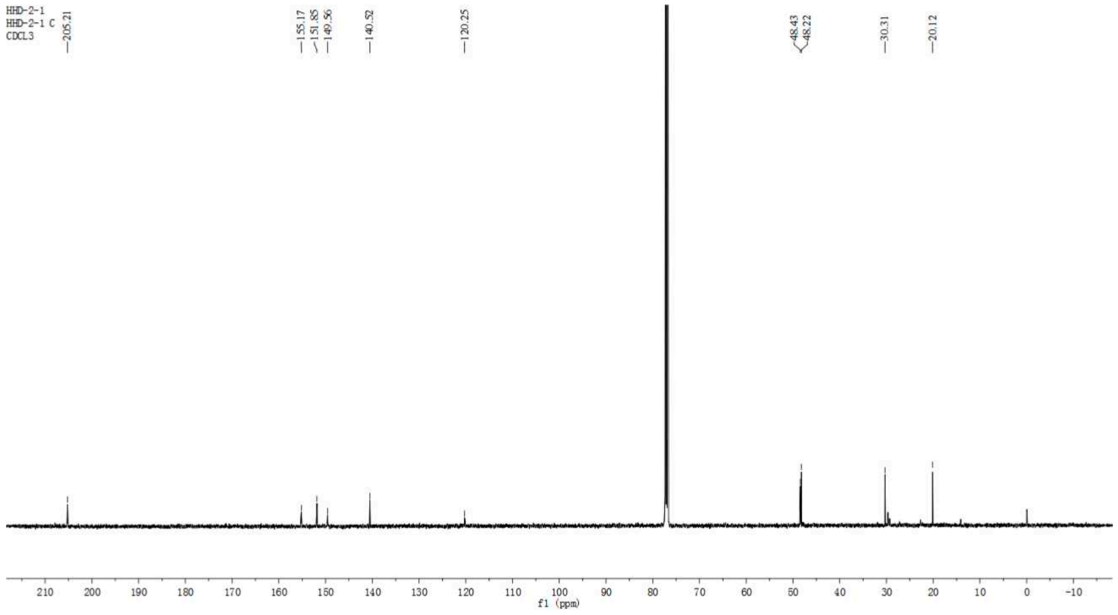

Figure S51. ^{13}C NMR spectrum (125MHz, CD_3OD) of **14**

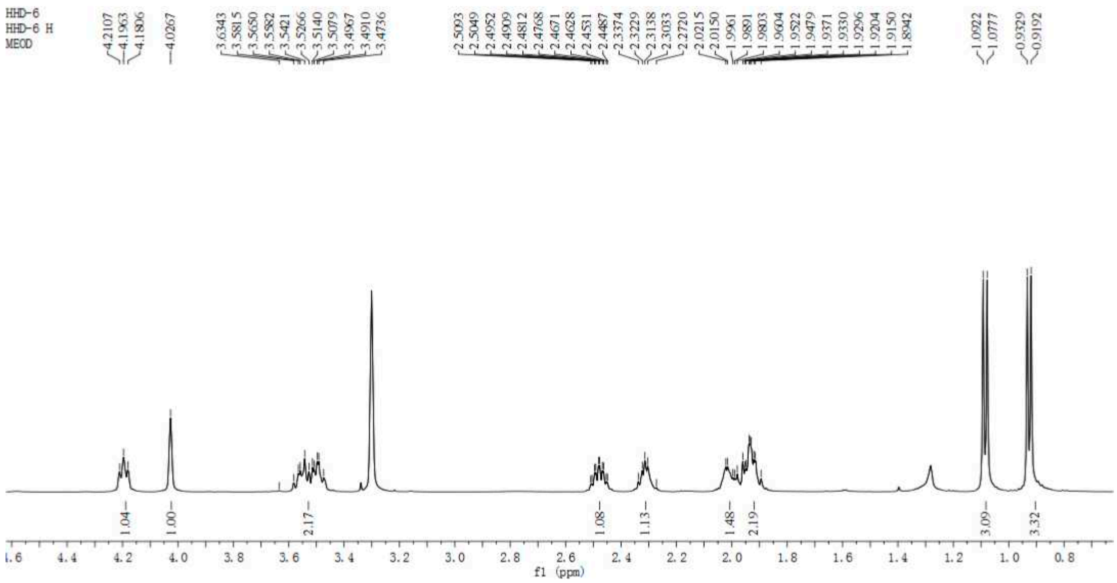

Figure S52. ^{1}H NMR spectrum (500MHz, CD_3OD) of **15**

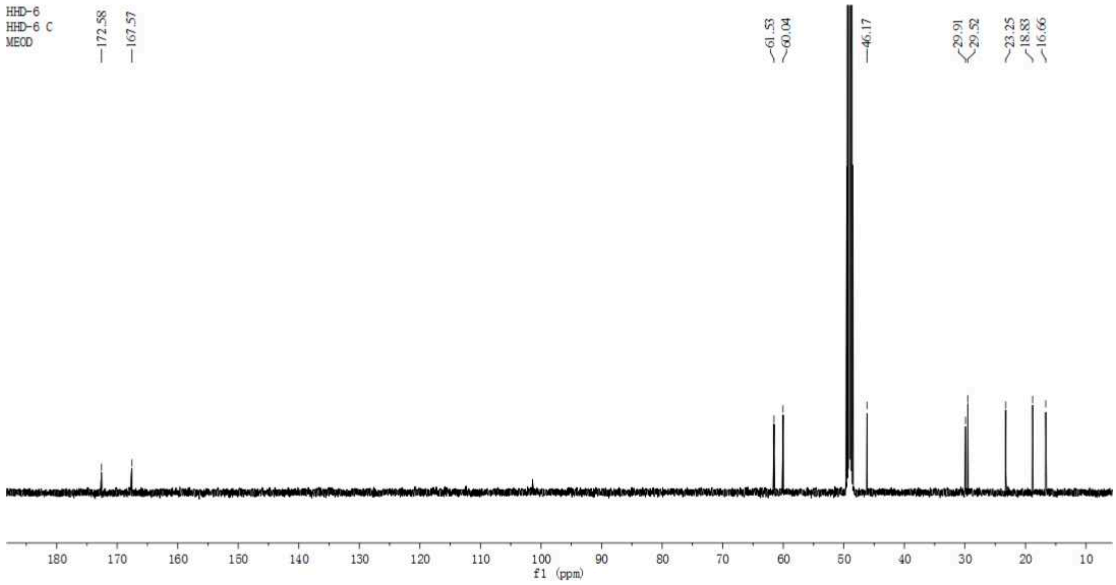

Figure S53. ^{13}C NMR spectrum (125MHz, CD_3OD) of **15**

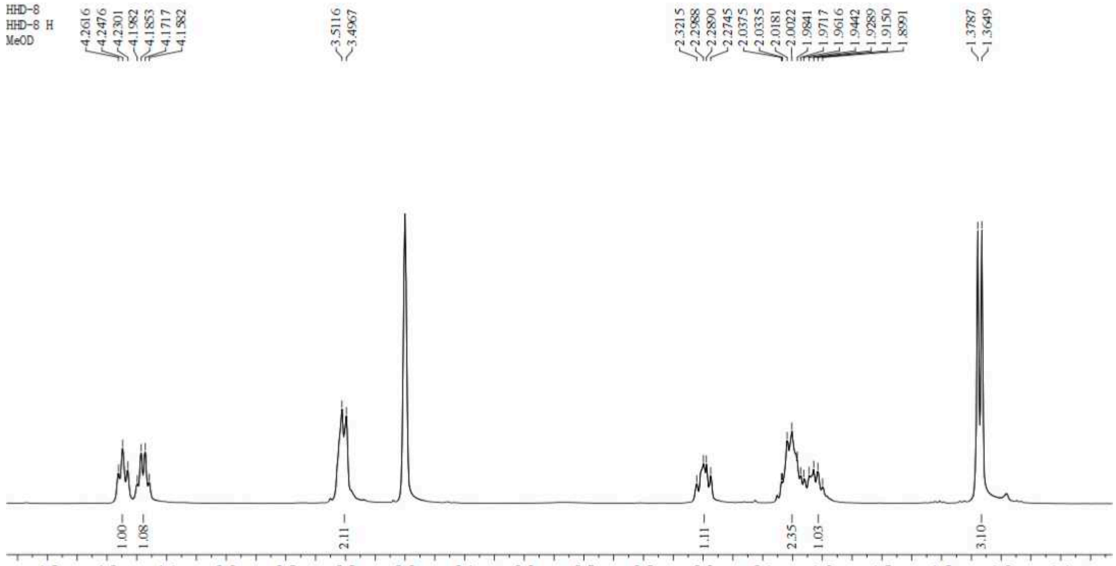

Figure S54. ^{13}C NMR spectrum (125MHz, CD_3OD) of **16**

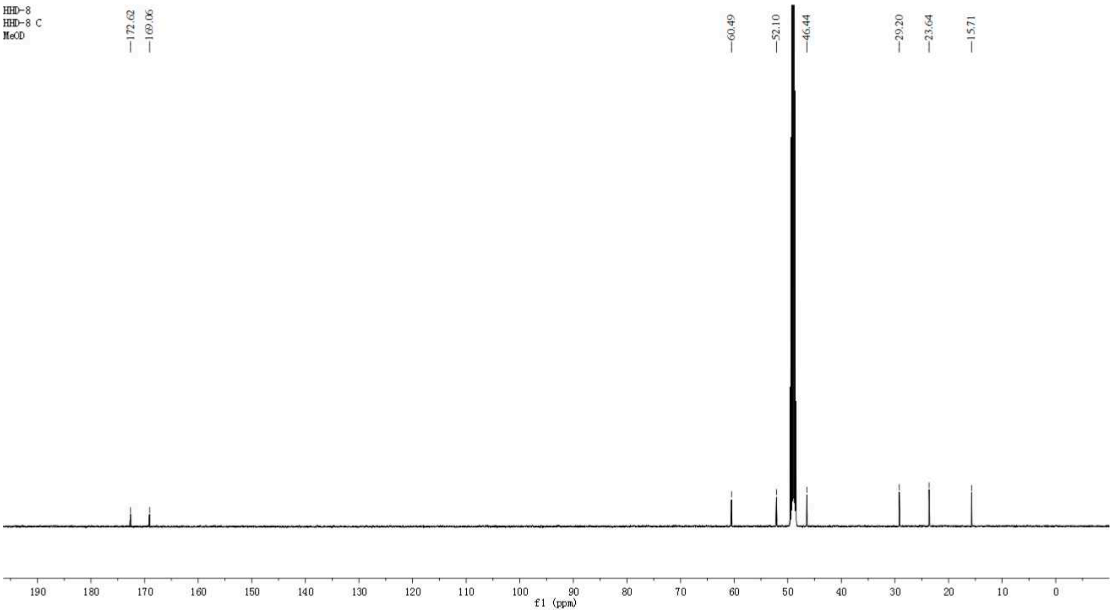

Figure S55. ^{13}C NMR spectrum (125MHz, CD_3OD) of **16**

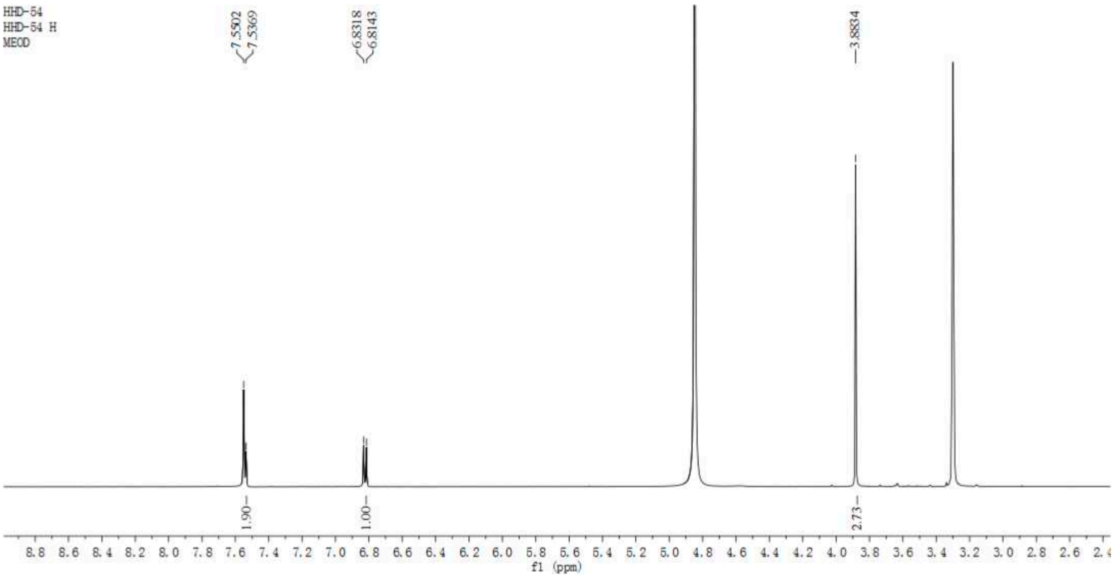

Figure S56. ^{1}H NMR spectrum (500MHz, CD_3OD) of **17**

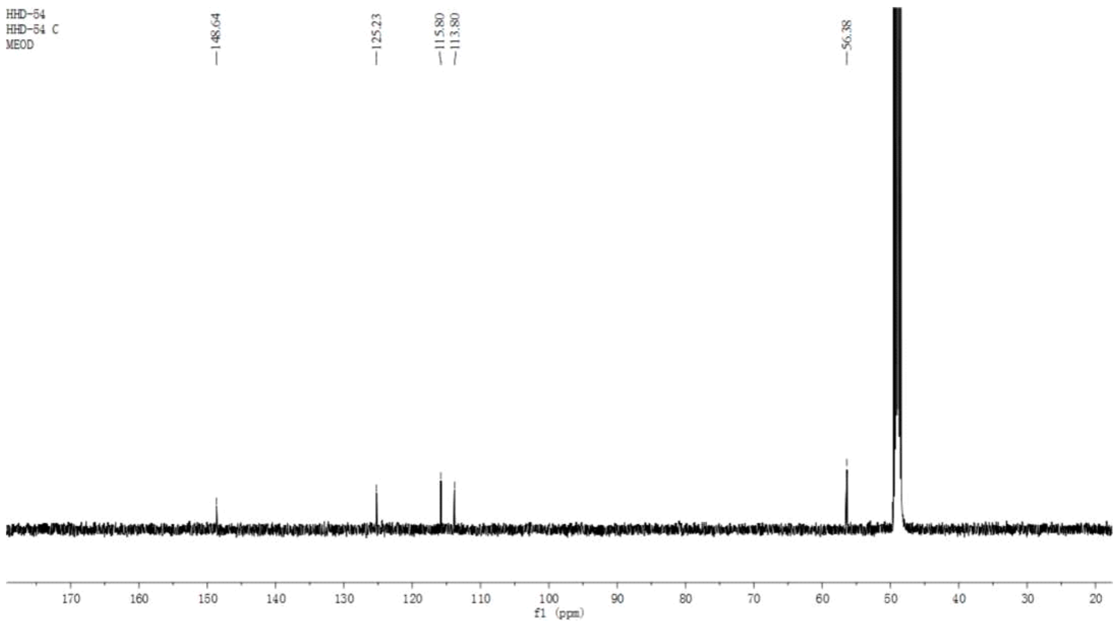

Figure S57. ^{13}C NMR spectrum (125MHz, CD_3OD) of **17**

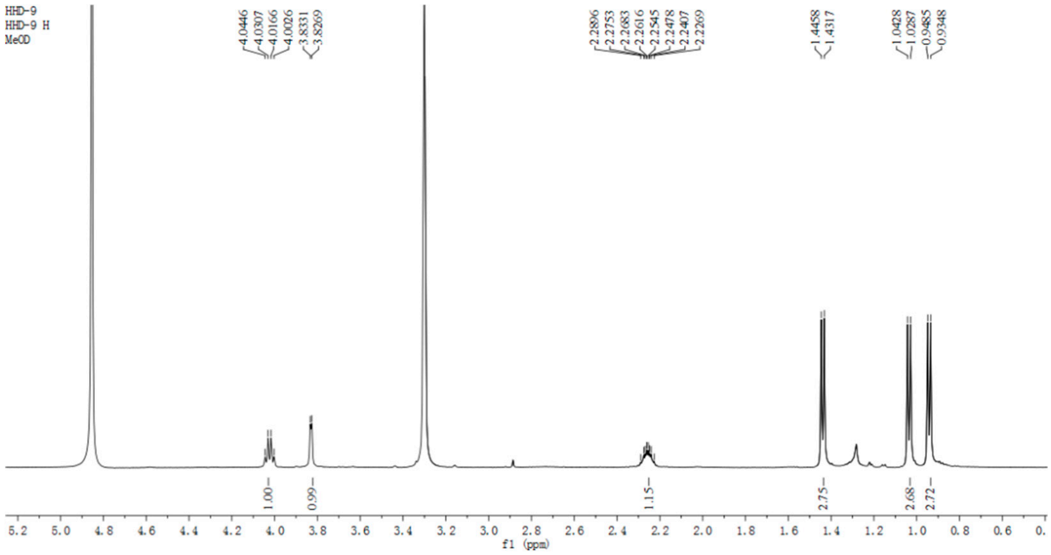

Figure S58. ^{1}H NMR spectrum (500MHz, CD_3OD) of **18**

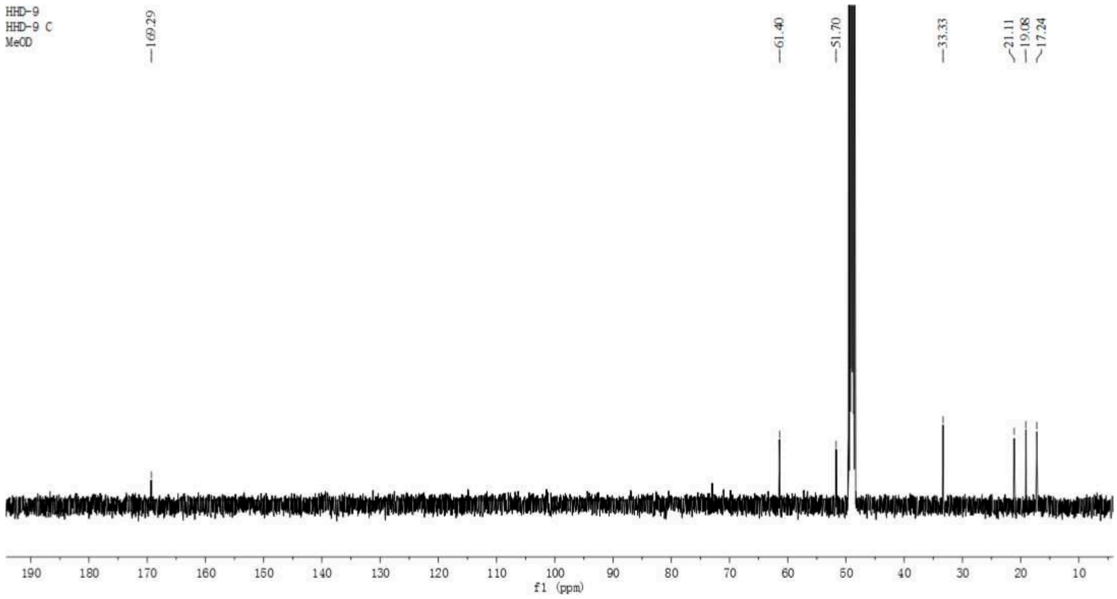

Figure S59. ^{13}C NMR spectrum (125MHz, CD_3OD) of **18**

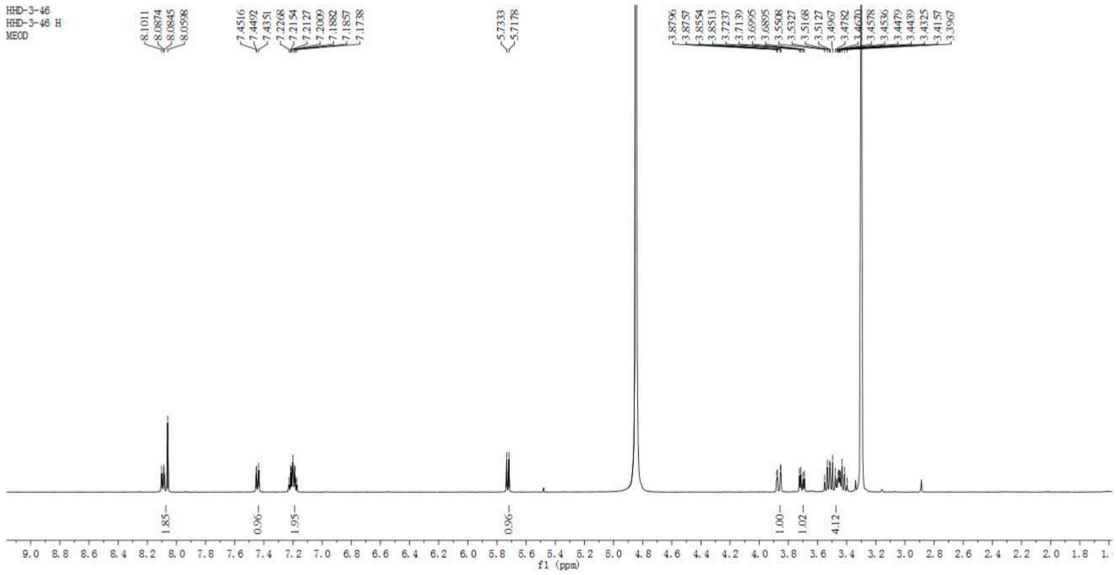

Figure S60. ^{1}H NMR spectrum (500MHz, CD_3OD) of **19**

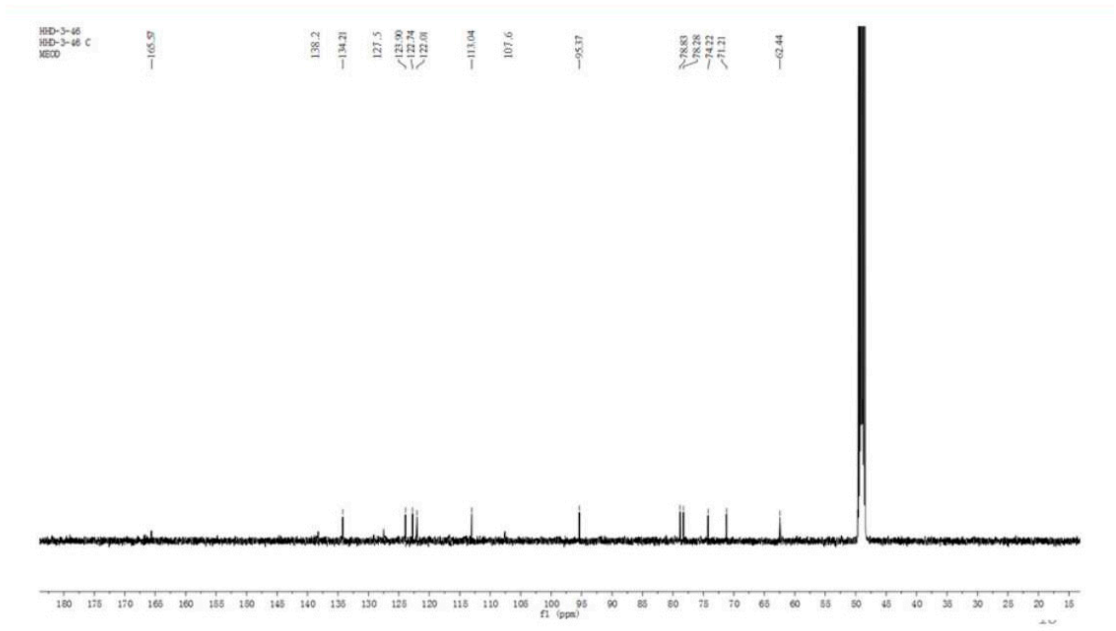

Figure S61. ^{13}C NMR spectrum (125MHz, CD_3OD) of **19**

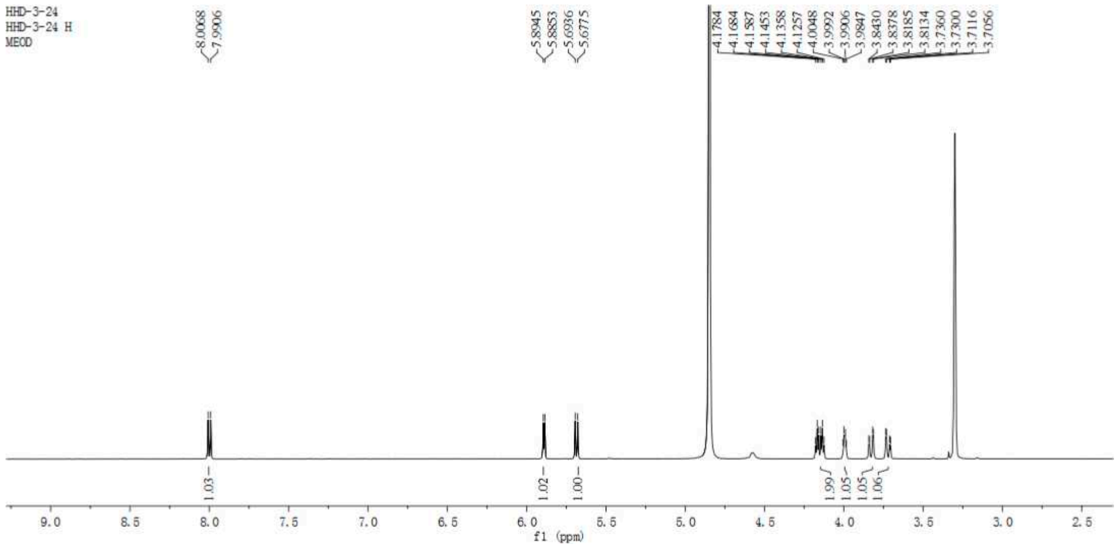

Figure S62. ^{1}H NMR spectrum (500MHz, CD_3OD) of **20**

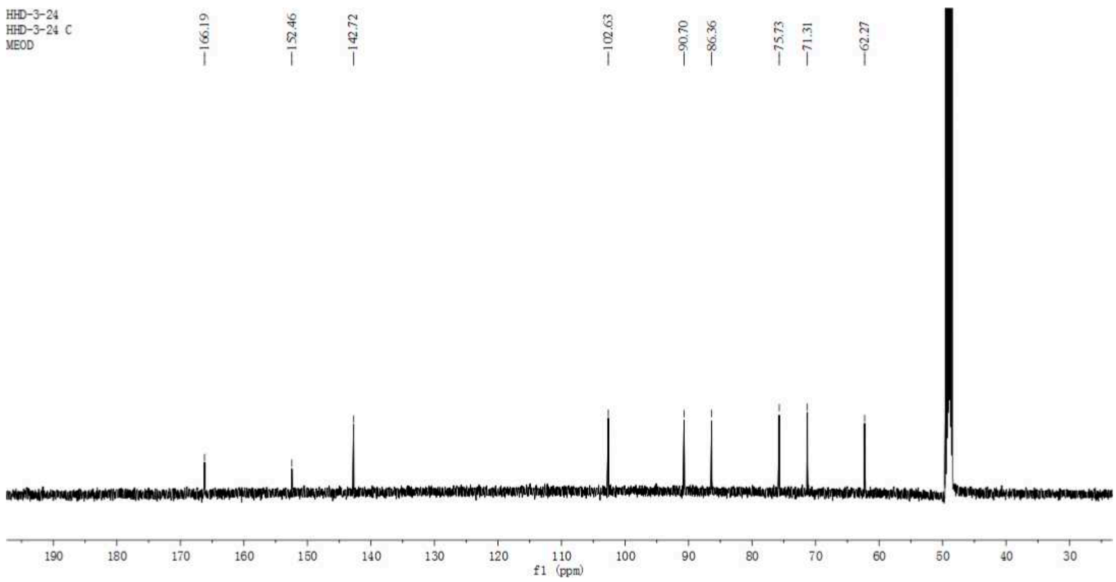

Figure S63. ^{13}C NMR spectrum (125MHz, CD_3OD) of **20**

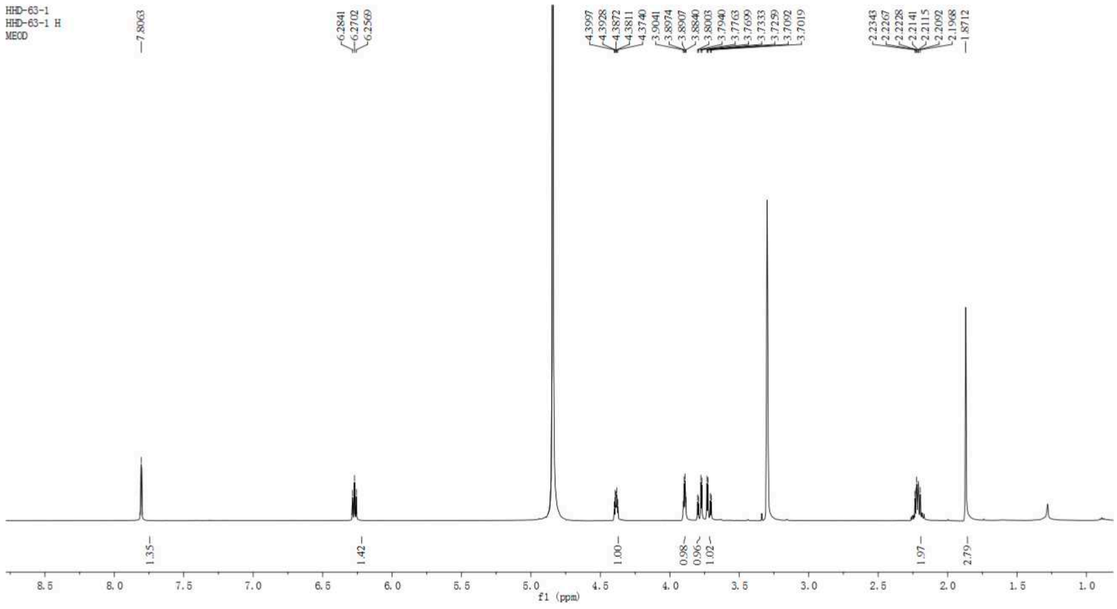

Figure S64. ^{1}H NMR spectrum (500MHz, CD_3OD) of **21**

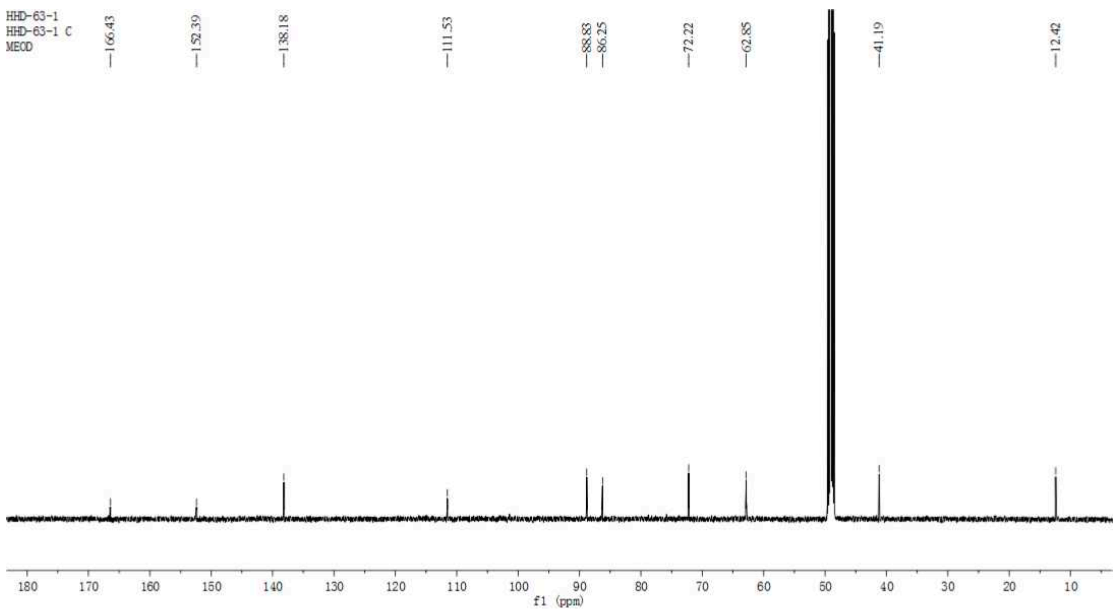

Figure S65. ^{13}C NMR spectrum (125MHz, CD_3OD) of **21**

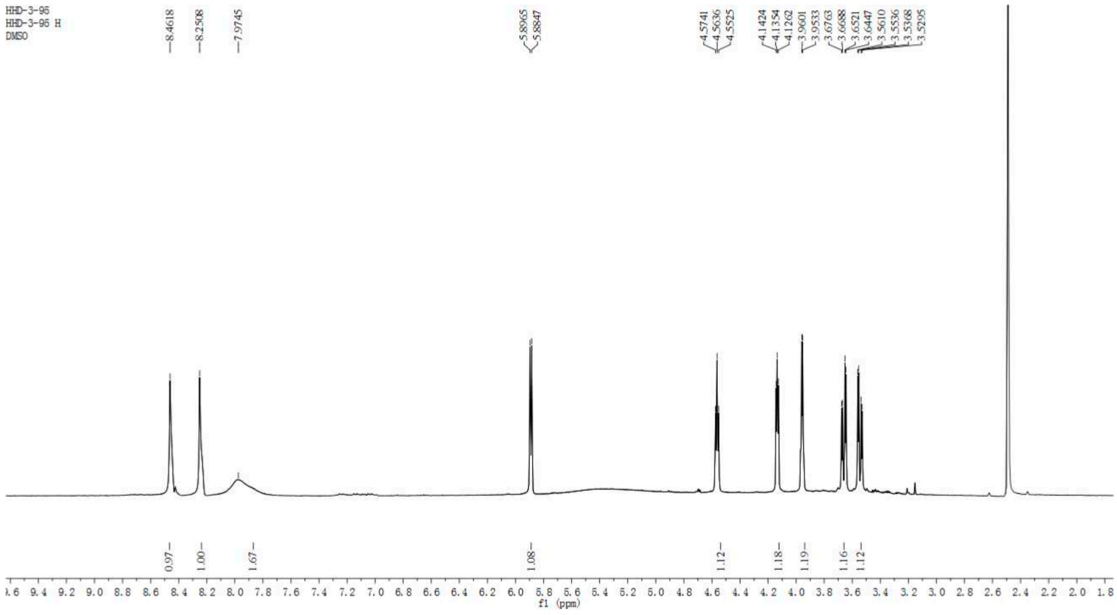

Figure S66. ^{1}H NMR spectrum (500MHz, DMSO) of **22**

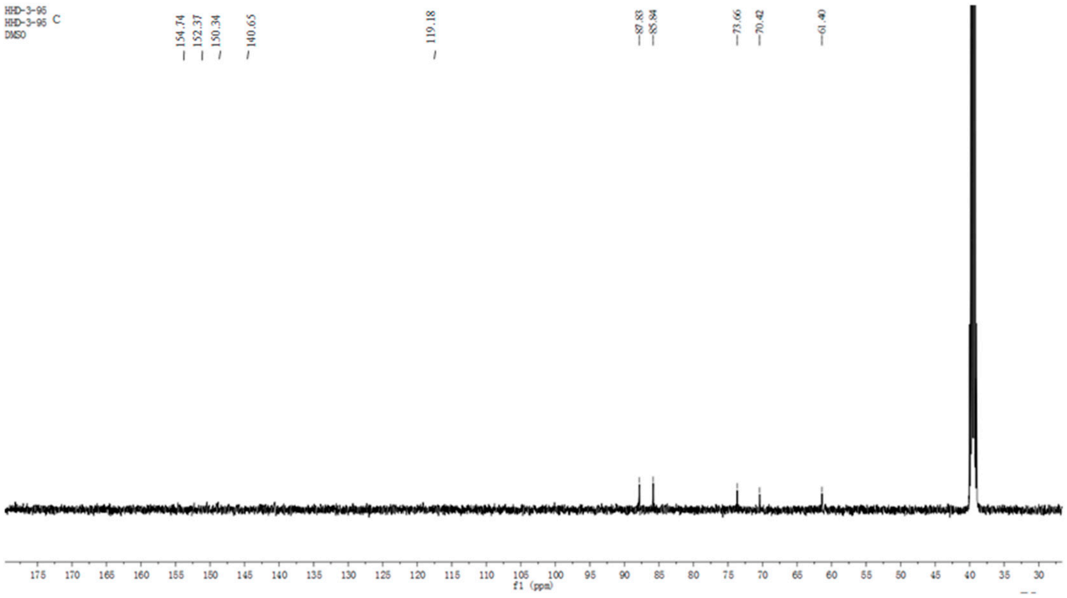

Figure S67. ^{13}C NMR spectrum (125MHz, DMSO) of **22**

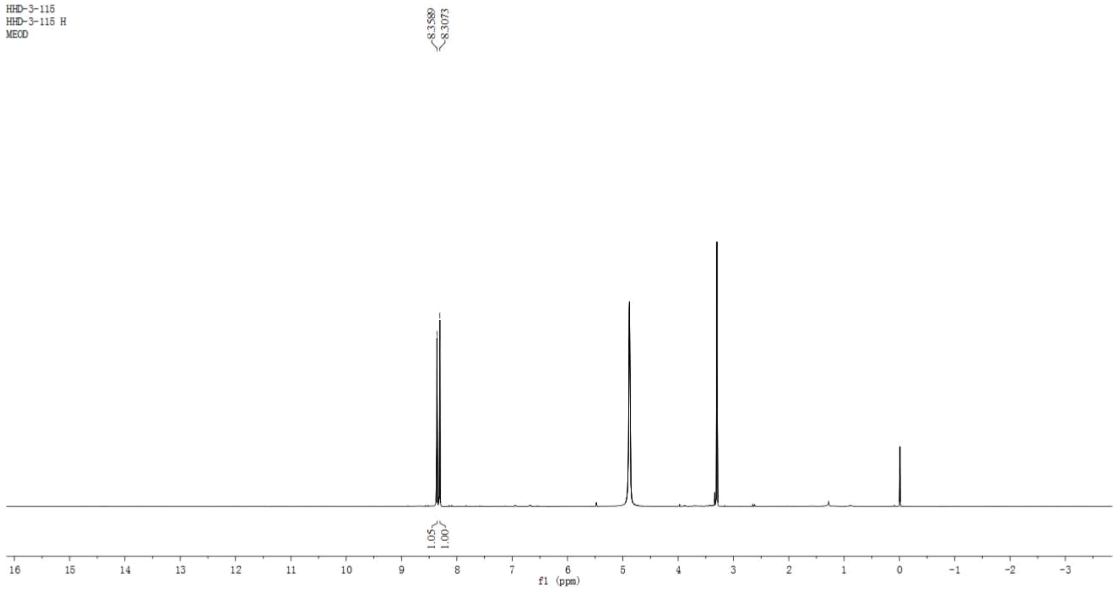

Figure S68. ^{1}H NMR spectrum (500MHz, CD_3OD) of **23**

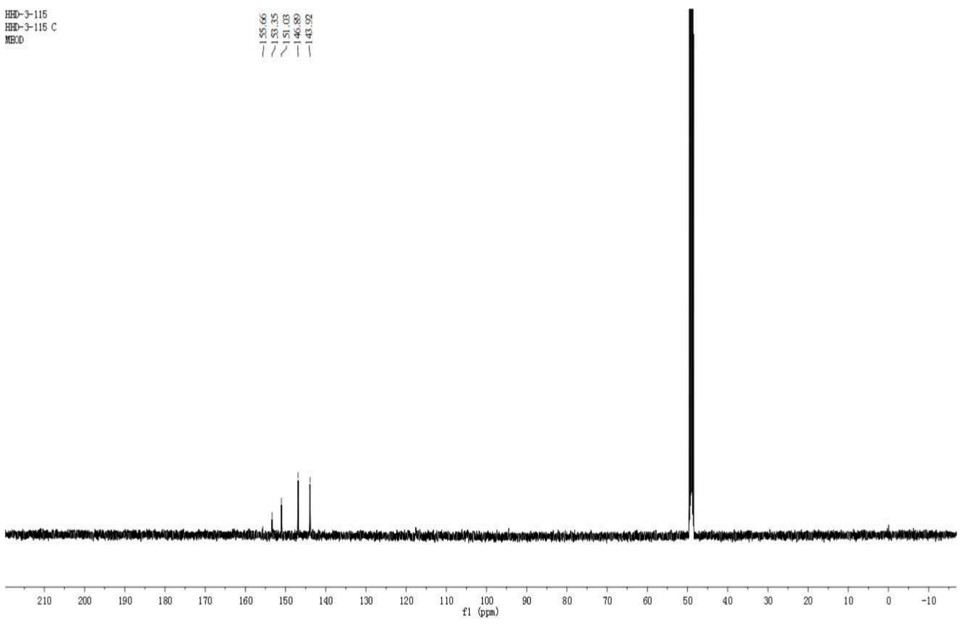

Figure S69. ^{13}C NMR spectrum (125MHz, CD_3OD) of **23**

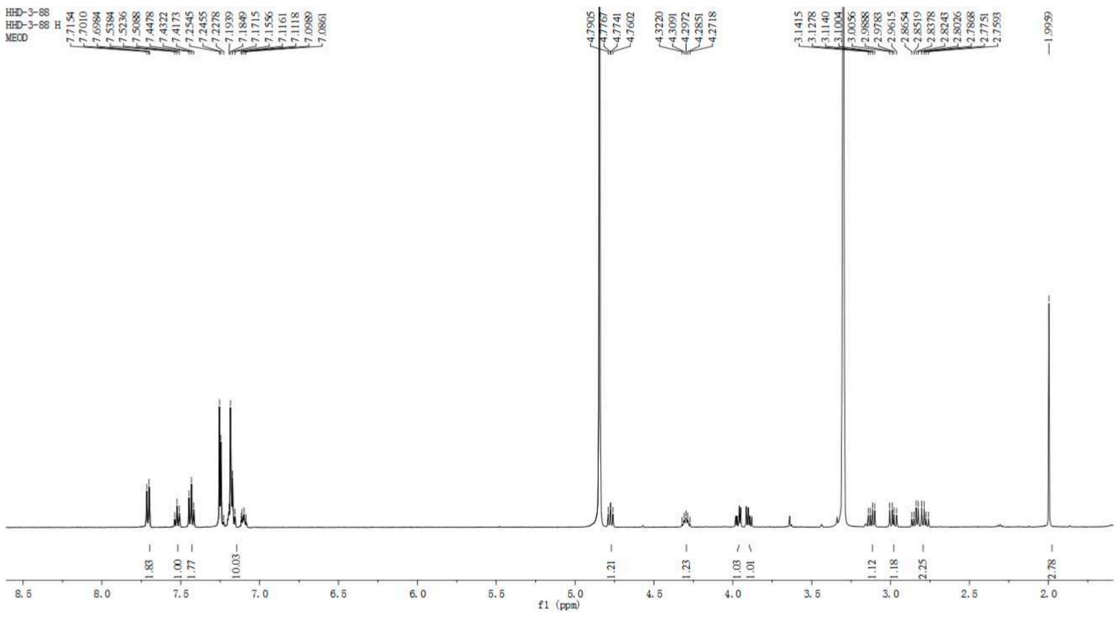

Figure S70. ^{1}H NMR spectrum (500MHz, CD_3OD) of **24**

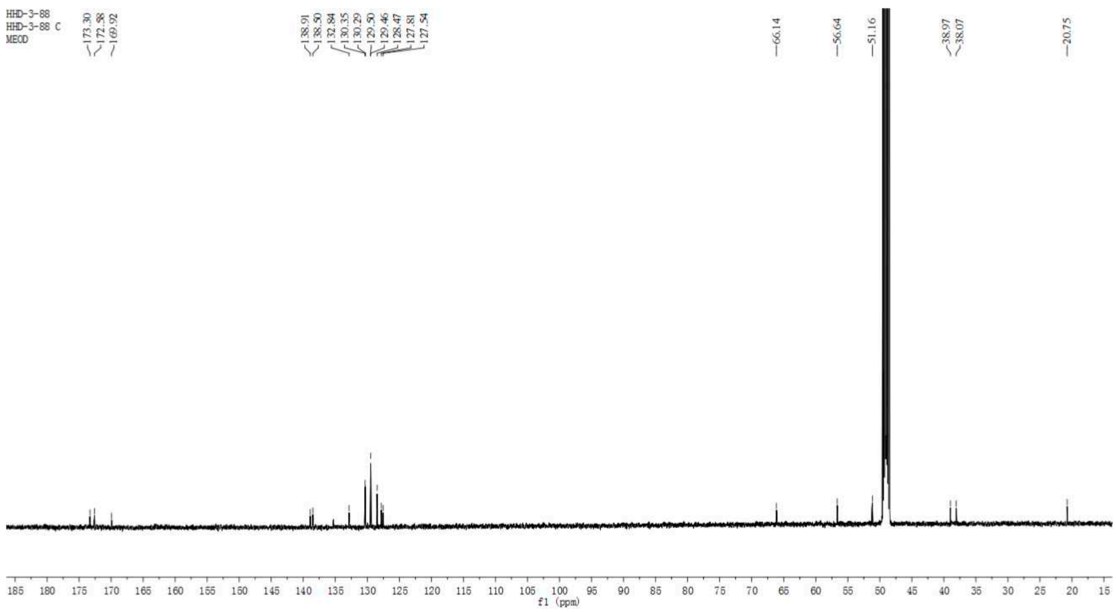

Figure S71. ^{13}C NMR spectrum (125MHz, CD_3OD) of **24**

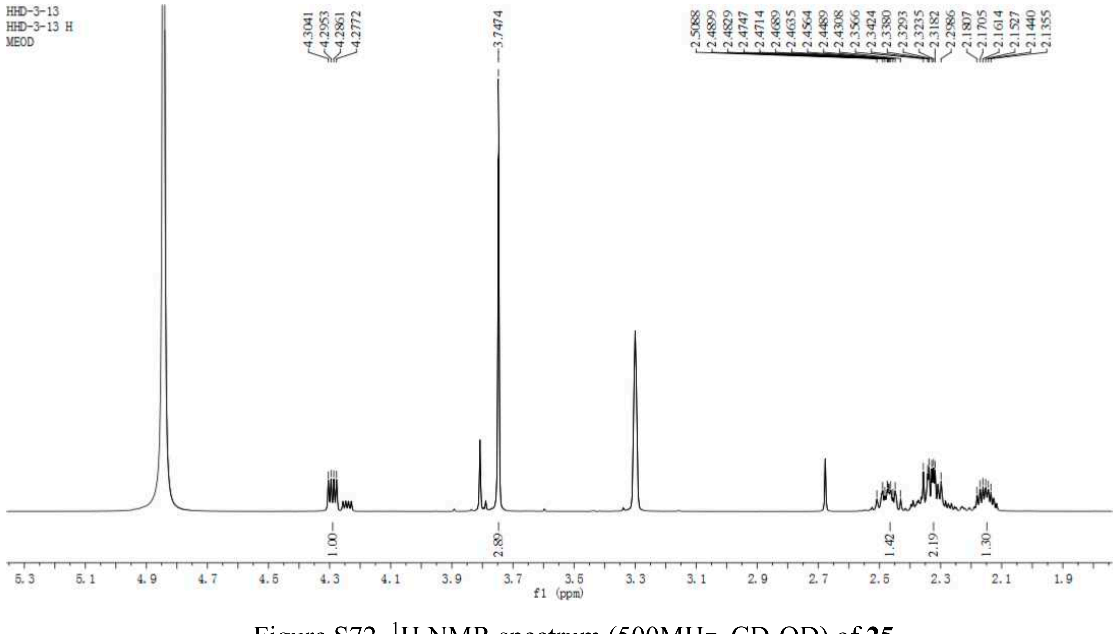

Figure S72. ^{1}H NMR spectrum (500MHz, CD_3OD) of **25**

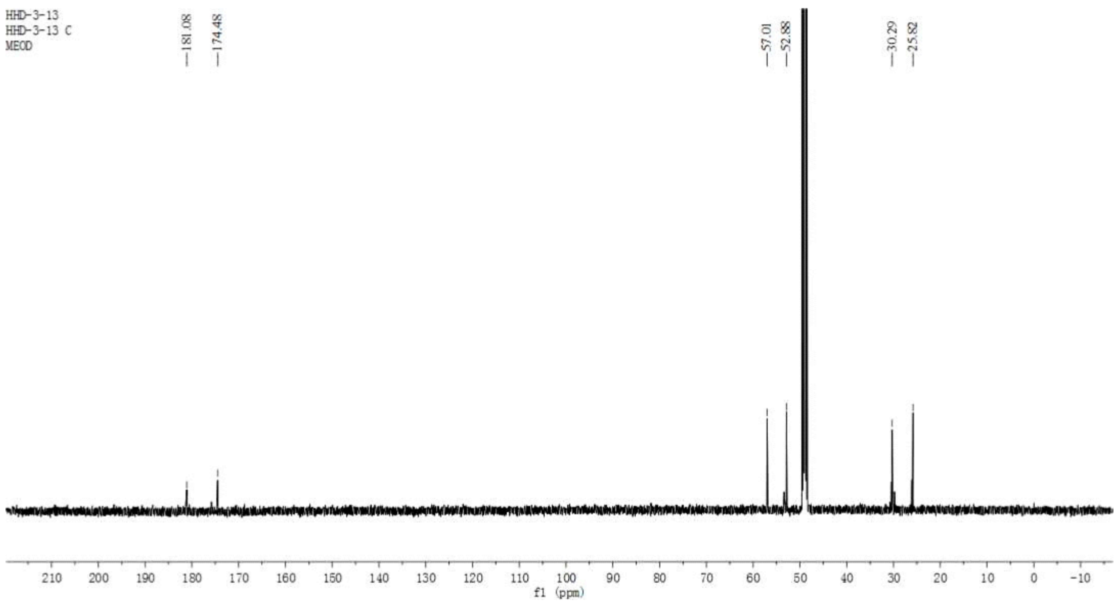

Figure S73. ^{13}C NMR spectrum (125MHz, CD_3OD) of **25**

Functional		Solvent?		Basis Set	
B3LYP		PCM		6-31G(d)	
		DP4+	100.00%	0.00%	-
Nuclei	sp2?	xperimenta	Isomer 1	Isomer 2	Isomer
C		41.9	49.4	38.3	
C		53.7	57.5	45.9	
C	x	202	205.7	183.6	
C	x	129	134.3	116.6	
C	x	159.8	169.2	147.4	
C	x	144.5	154.6	133.5	
C	x	130.3	141.2	121.6	
C		38.7	39.2	33.1	
C		76	79.2	68.3	
C		20	22.9	13.0	
C		25.1	28.7	20.7	
C		28.4	29.28	24.28	
C		28.3	31.81	21.78	
C		102.6	106.40	94.08	
C		75.1	79.75	67.80	
C		78.1	82.66	69.88	
C		72.4	80.26	63.41	
C		77	79.75	68.01	
C		68.8	78.68	56.91	
C		102.4	109.49	93.27	
C		72	77.32	61.97	
C		72.2	77.93	65.63	
C		74	79.12	66.15	
C		69.9	76.06	66.44	
C		18.1	18.75	13.23	
H		2.28	2.05563715	1.72347535	
H		2.28	2.39275267	2.1605873	
H	x	5.88	5.95634936	5.32765763	
H	x	5.96	6.48006578	5.66954786	
H		2.52	2.93998516	2.06331996	
H		2.62	2.49976047	2.65153556	
H		3.93	4.12929116	3.76999751	
H		1.23	1.02887391	0.78317192	
H		1.23	1.30751465	1.12086781	
H		1.23	1.26461466	0.86157543	
H		2.26	2.06244768	2.286052	
H		2.26	2.64621071	1.71005785	
H		2.26	2.11902582	1.82684593	
H		1.18	0.86900564	1.16881973	
H		1.18	0.76683892	0.86306038	
H		1.18	1.62145154	0.72428876	
H		1.17	1.19682135	0.67409498	
H		1.17	1.4270653	0.77823485	
H		1.17	1.0034881	1.30616816	
H		4.32	4.63969719	4.18838759	
H		3.13	3.52624609	2.9393927	
H		3.32	3.53880726	3.30514804	
H		3.64	3.49033169	3.56822597	
H		3.4	3.37107807	3.11337563	
H		3.54	3.87815433	3.58713306	
H		3.98	4.20570381	3.55083506	
H		4.73	4.71466051	4.49987246	
H		3.19	3.32441103	3.08546646	
H		3.79	4.40768629	3.89374524	
H		3.36	3.34782927	2.82572846	
H		3.62	3.83171796	3.48320712	
H		1.25	1.33174371	1.1522271	
H		1.25	0.97771454	0.64589803	
H		1.25	1.50758103	0.99718943	

Figure S74. DP4+ evaluation of theoretical and experimental data of **2**
